# Ionic organic molecular cage-supported Pd nanoparticles for aqueous Suzuki–Miyaura coupling

**DOI:** 10.1039/d6cc03901d

**Published:** 2026-09-21

**Authors:** Brandon Bishop, Xiaoyu Fang, Simon Teat, Zhehao Yuan, Shuyan Jiang, Alec Kolodziejczyk, Qiucheng Xu, Wei Zhang

**Affiliations:** a Department of Chemistry, University of Colorado Boulder Boulder CO 80309 USA wei.zhang@colorado.edu; b School of Physical Sciences, Great Bay University, Songshan Lake Park Dongguan 523000 P.R.China; c Advanced Light Source, Department of Chemistry, Lawrence Berkeley National Laboratory, University of California Berkeley CA 94720 USA

## Abstract

Spiroborate-linked organic molecular cages bearing bipyridine and phenanthroline ligands stabilize palladium nanoparticles whose dimensions are consistent with exocyclic growth. The cage-supported Pd nanoparticles act as efficient, recyclable catalysts for aqueous Suzuki–Miyaura coupling, affording high conversions under mild conditions while maintaining cage integrity, nanoparticle morphology, and activity over multiple cycles.

## Abstract 2

Spiroborate-linked ionic organic molecular cages with N-donor sites stabilize Pd nanoparticles *via* exocyclic binding interactions. The resulting Pd-NP@OMCs act as efficient and recyclable catalysts for aqueous Suzuki–Miyaura coupling.

## Graphical abstract

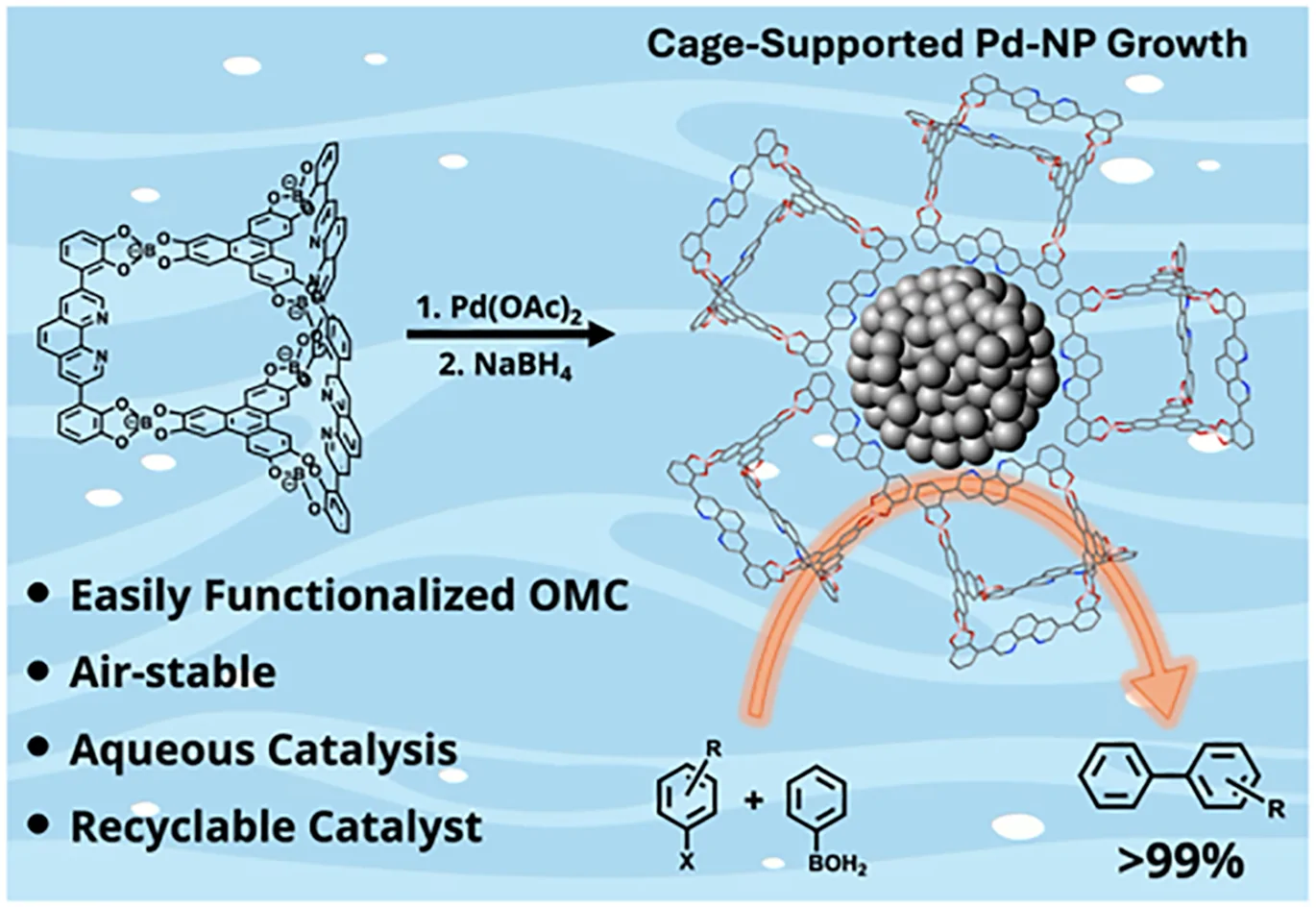

Palladium-catalyzed cross-coupling reactions are among the most widely utilized transformations in synthetic chemistry and enable efficient C–C and C–N bond formation in pharmaceutical, agrochemical, and materials synthesis.^1–6^ However, developing catalytic systems that simultaneously combine high activity, stability, recyclability, and sustainability remains a significant challenge.^7,8^ Palladium nanoparticles (Pd-NPs) have attracted considerable interest because their high surface-area-to-volume ratios increase active-site accessibility while enabling tunable catalytic properties through control of nanoparticle size, morphology, and electronic structure.^9–15^ When effectively stabilized or immobilized, Pd-NPs can also facilitate catalyst recovery and recycling.^16–18^ However, their high surface energy promotes aggregation and loss of catalytic activity, necessitating stabilizing architectures capable of controlling nanoparticle nucleation and growth.^19–22^

Pd-NPs are commonly stabilized using soluble ligands or dendritic architectures,^23–25^ or immobilized on solid supports including metal–organic frameworks (MOFs),^26–29^ covalent organic frameworks (COFs),^30–33^ and porous polymers.^34,35^ While effective, these systems may introduce diffusion limitations, partially obstruct the nanoparticle surface, or require complex synthetic procedures. Discrete organic molecular cages (OMCs) offer an alternative platform with structurally defined and tunable coordination environments that may influence NP nucleation and growth without requiring an extended solid support.^36–44^ Dynamic covalent chemistry (DCvC) is widely used to synthesize OMCs, because reversible bond formation enables error correction during cage assembly.^45–48^ Unlike extended porous solids, many OMCs are soluble or dispersible, allowing them to be incorporated into solution-phase catalytic systems.^49–52^ Their discrete structures, accessible windows, and synthetically tunable binding sites make them attractive platforms for catalyst design.^53–58^ In principle, the open windows of a discrete cage may preserve access to a greater portion of the NP surface than a continuous polymeric support.^59–62^ Despite these advantages, the influence of cage structure and ligand geometry on Pd-NP nucleation, growth, and catalytic performance remains largely unexplored.

Herein, we report spiroborate-linked ionic OMCs functionalized with bipyridine (BPY) and phenanthroline (PHEN) ligands that coordinate Pd precursors and stabilize Pd-NPs for aqueous Suzuki–Miyaura coupling. The OMCs are assembled through one-step dynamic spiroborate exchange and contain multiple nitrogen-donor binding sites. The ionic nature of the spiroborate linkage imparts water dispersibility and stability under basic conditions, allowing the cages to be used directly under aqueous coupling conditions.^63–66^ A *p*-quaterphenyl-functionalized cage lacking nitrogen-donor sites, QP-OMC, was synthesized as a control.

The [3+2] spiroborate-linked OMCs were synthesized in one-pot by reacting two equivalents of the tritopic 2,3,6,7,10,11-hexahydroxytriphenylene (HHTP) panel with three equivalents of the corresponding dicatechol-based pillar (QP-4OH, BPY-4OH, or PHEN-4OH) using boric acid in dimethylformamide (DMF). The tetrahedral spiroborate linkage places the two diol units at an angle of approximately 90°, which is compatible with the formation of a trigonal prismatic cage architecture.^67–70^ The reversibility of spiroborate formation allows error correction during assembly and thermodynamically favored formation of the cage product.^69,70^ All three OMCs were obtained in high yields (90% QP-OMC, 88% BPY-OMC, and 71% PHEN-OMC).

The structures of all three OMCs were characterized by NMR spectroscopy (^1^H, ^13^C, ^11^B), FT-IR, and electrospray ionization high-resolution mass spectrometry (ESI-HRMS), and their thermal behavior was evaluated using capillary melting-point measurements. Single crystals suitable for X-ray diffraction were also obtained for QP-OMC. Single-crystal X-ray diffraction (SCXRD) analysis of QP-OMC confirmed the formation of a trigonal prismatic cage with an internal cavity of approximately 1.5 × 1.3 nm (Fig. 1B and Fig. S1B, C). Suitable single crystals of BPY-OMC and PHEN-OMC were not obtained, so their approximate geometries were evaluated computationally. Energy-minimized models suggested overall dimensions similar to those of QP-OMC.

**Fig. 1:**
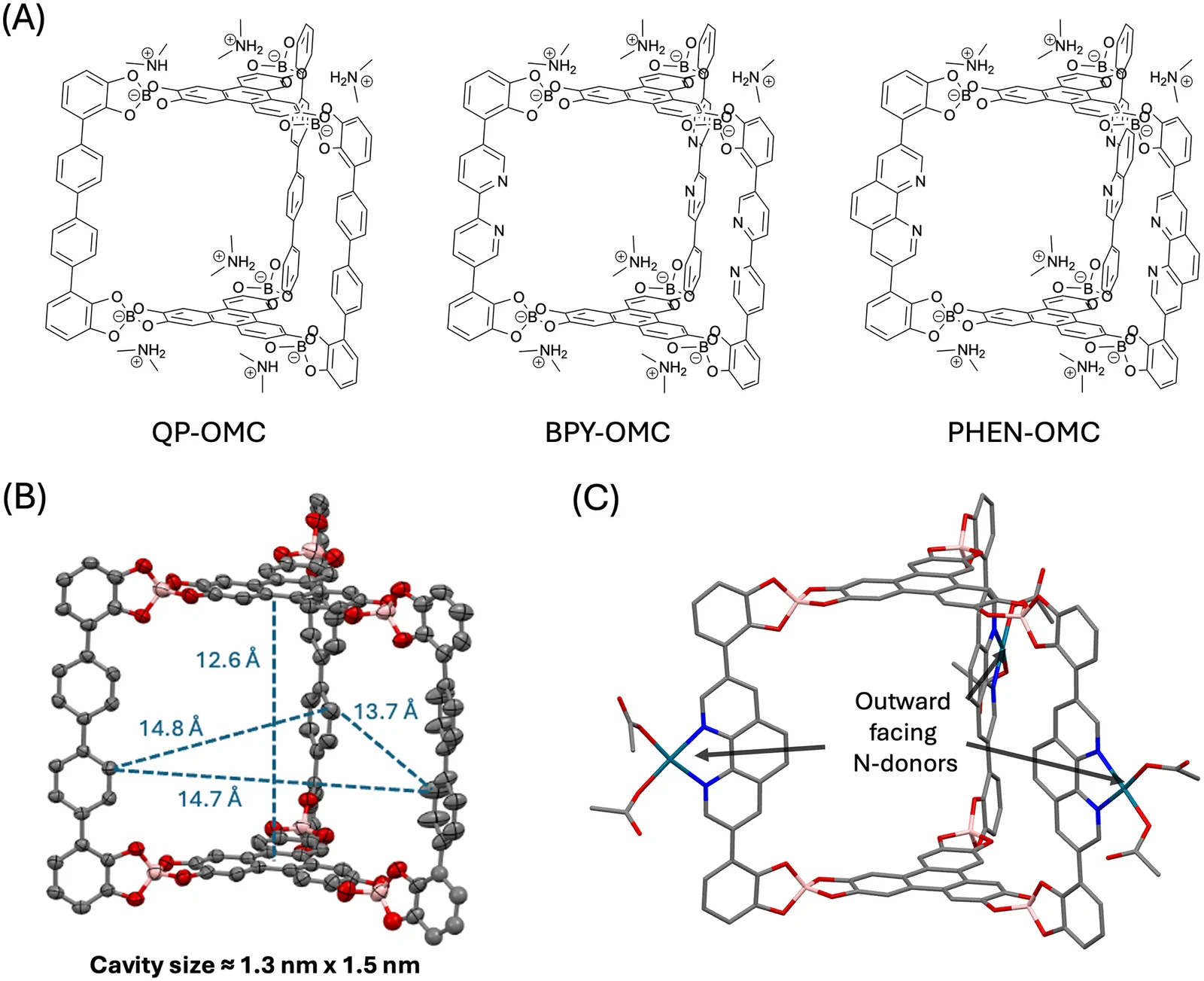
Structural design and characterization of the spiroborate-linked OMCs. (A) Chemical structures of QP-OMC, BPY-OMC, and PHEN-OMC. (B) Single-crystal X-ray structure of QP-OMC showing internal dimensions of approximately 1.3–1.5 nm. Thermal ellipsoids are shown at the 50% probability level. Hydrogen atoms, counterions, and solvent molecules are omitted for clarity. (C) Energy-minimized model of Pd@PHEN-OMC illustrating the outward orientation of the nitrogen-donor sites upon Pd coordination.

The energy-minimized structures suggested different preferred orientations of the nitrogen-donor sites in the two functionalized cages (Table S1). In the model of BPY-OMC, the flexible BPY units can adopt an inward-facing nitrogen orientation in the absence of Pd. In contrast, the more rigid PHEN units favor an outward-facing orientation, likely because of steric constraints. In the Pd-coordinated models, the nitrogen donors of both cages orient toward the cage exterior (Fig. 1C and Fig. S4), indicating that peripheral Pd binding is more geometrically accessible. On this basis, Pd-NP formation is proposed to proceed through initial coordination of Pd(OAc)_2_ to the peripheral BPY or PHEN sites followed by reduction with NaBH_4_ to form cage-supported Pd-NPs (Fig. 1C). The outward-facing nitrogen donors provide potential nucleation and stabilization sites at the cage periphery. The resulting NPs are substantially larger than the cage cavity, as discussed below, making the data more consistent with external cage-NP association than with NP encapsulation. Multiple cages are therefore proposed to interact with and stabilize the nanoparticle surface through their nitrogen-donor sites.^16,61,62^

Cage-supported Pd-NPs were prepared following literature procedures (Fig. S5).^54,62^ Ultraviolet–visible (UV-vis) spectroscopy was used to monitor changes accompanying Pd coordination and subsequent reduction. Upon addition of Pd(OAc)_2_, the cage absorption bands decreased in intensity and new broad features appeared, consistent with Pd coordination to the nitrogen-donor sites (Fig. S6).^60,71,72^ The BPY-OMC system exhibited a lower-energy absorption feature than PHEN-OMC, indicating different electronic interactions between Pd and the two ligand environments. Subsequent reduction caused additional peak broadening and spectral shifts, consistent with a change in the Pd-cage interaction during nanoparticle formation. Changes in the ^1^H NMR spectra provided additional evidence for interaction between the cages and Pd species. Addition of Pd(OAc)_2_ produced substantial peak broadening and chemical-shift changes, consistent with dynamic Pd(ii) coordination and changes in the electronic environment of the nitrogen-donor units. Following reduction, the resonances became sharper but remained shifted relative to the free cages, indicating that the cages remained associated with the Pd species after nanoparticle formation (Fig. S6).^60^

Inductively coupled plasma optical emission spectrometry (ICP-OES) measurements gave Pd loadings of approximately 9–10 wt% (Table S2), consistent with the amount of Pd(OAc)_2_ used during preparation. These loadings are below the approximately 20 wt% threshold associated with increased agglomeration in previously reported supported-Pd systems.^16,73^ Transmission electron microscopy (TEM) revealed uniform Pd-NPs for both cages, with PHEN-OMC producing smaller Pd-NPs and a narrower size distribution (4.7 ± 0.5 nm) compared to BPY-OMC (7.7 ± 0.7 nm) (Fig. 2). Both particle sizes substantially exceed the cage cavity dimensions, ruling out encapsulation of the Pd-NPs within a single cage and supporting an external growth model.^16,54,62^ The smaller Pd-NPs obtained with PHEN-OMC may arise from its more rigid and preorganized nitrogen-donor environment, which could favor more localized Pd coordination and a greater nucleation density (Fig. 2A). The more flexible BPY units may permit greater Pd mobility before stabilization, resulting in larger NPs (Fig. 2C).^74–76^

**Fig. 2:**
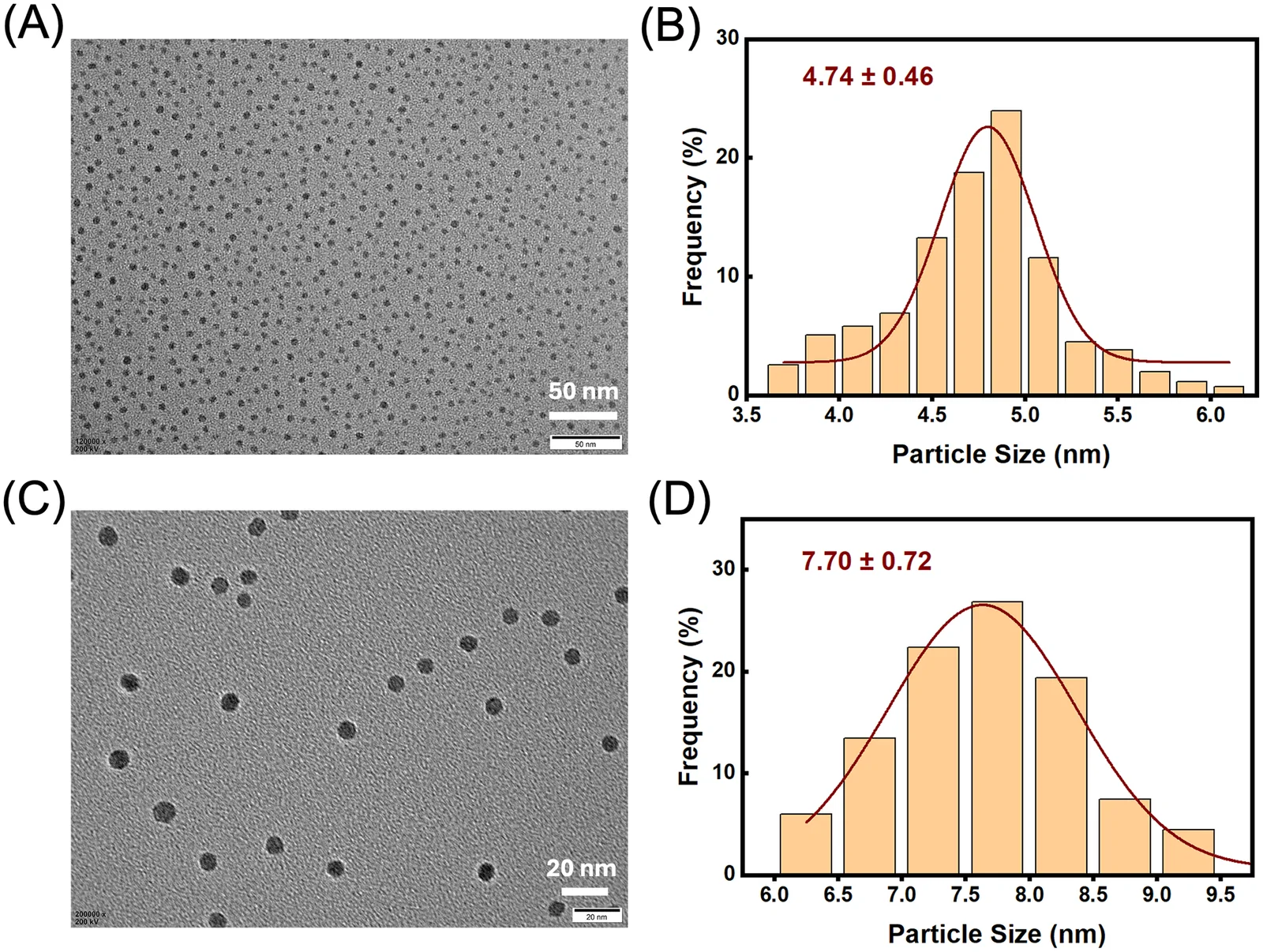
Transmission electron microscopy (TEM) analysis of cage-supported Pd-NPs. (A) TEM image of Pd-NP@PHEN-OMC (scale bar = 50 nm). (B) Particle size distribution of Pd-NP@PHEN-OMC (4.74 ± 0.46 nm). (C) TEM image of Pd-NP@BPY-OMC (scale bar = 20 nm). (D) Particle size distribution of Pd-NP@BPY-OMC (7.70 ± 0.72 nm).

The Pd-NP@OMC materials formed stable DMSO solutions for several days without noticeable precipitation, enabling solution-phase UV-vis and NMR characterization and further supporting effective stabilization of the Pd-NPs by the cages.^54,60^ Control experiments were used to evaluate the contributions of the cage scaffold and nitrogen-donor functionality to Pd-NP stabilization. In the absence of an OMC, reduction of Pd(OAc)_2_ produced large aggregates (Fig. S7A). Similarly, QP-OMC, which lacks nitrogen-donor sites, did not prevent formation and precipitation of larger Pd species (Fig. S7B). The isolated BPY and PHEN pillar compounds likewise failed to prevent aggregation upon reduction (Fig. S7C and D). Together, these controls indicate that both the nitrogen-donor sites and the multidentate cage architecture contribute to nanoparticle stabilization.^61^

The catalytic performance of the Pd-NP@OMC materials as heterogeneous catalysts was evaluated using aqueous Suzuki–Miyaura coupling reactions (Fig. 3A and Table S3). Heterogeneous catalysts are attractive because their facile separation can enable catalyst recovery and reuse.^16–18^ Aqueous catalysis also reduces reliance on organic solvents and aligns with green chemistry principles. Under the optimized conditions (0.5 mol% Pd, Na_2_CO_3_, tetrabutylammonium bromide (TBAB)), both catalysts afforded high to quantitative conversions for most substrates after 3 h at 90 °C. TBAB was used as a phase-transfer agent to facilitate contact between the hydrophobic aryl halides and the aqueous base and boronic acid components.^54^ A range of aryl iodides and bromides bearing electron-donating and electron-withdrawing substituents were successfully coupled with multiple phenylboronic acid derivatives, demonstrating broad substrate scope and consistently high conversions. The slightly higher activity of Pd-NP@PHEN-OMC observed in some cases may be associated with the smaller NP size and the more rigid coordination environment provided by the PHEN ligand.^12,16,61^ Microwave-assisted reactions were also performed to evaluate whether the coupling time could be reduced. Under microwave heating at 90 °C, the reaction time was reduced from 3 h to 30 min while maintaining high conversions for most substrates.

**Fig. 3:**
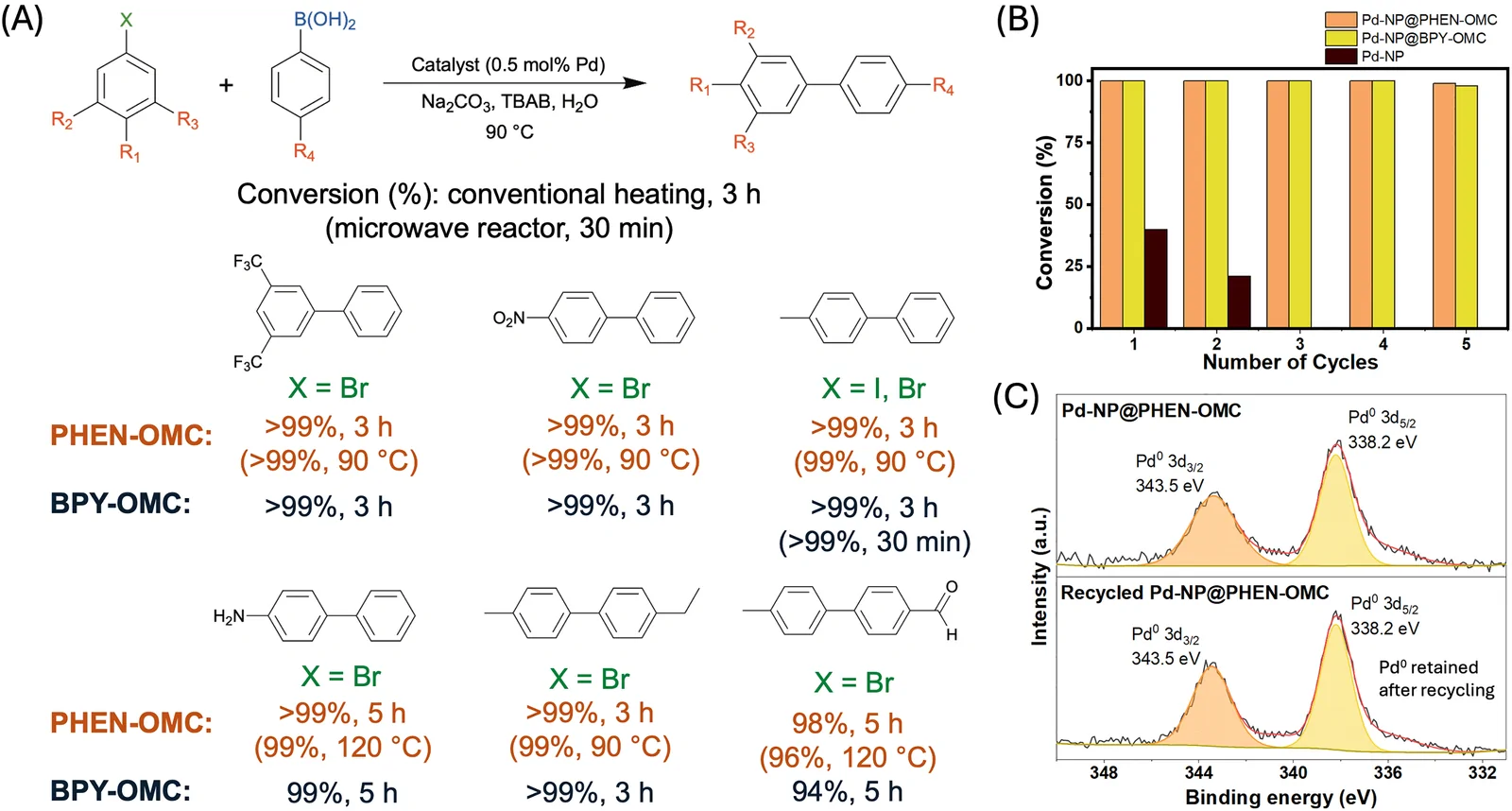
Catalytic performance and stability of Pd-NP@OMC systems. (A) Representative substrate scope for aqueous Suzuki–Miyaura coupling under conventional heating with 0.5 mol% Pd loading at 90 °C for 3 h, unless otherwise indicated; values in parentheses correspond to microwave-assisted reactions performed for 30 min at 90 °C unless otherwise indicated. Conversions were determined by ^1^H NMR analysis after extraction. (B) Recycling performance of Pd-NP@PHEN-OMC and Pd-NP@BPY-OMC over five cycles compared to unsupported Pd-NPs. (C) X-ray photoelectron spectroscopy (XPS) spectra of Pd-NP@PHEN-OMC before and after recycling, showing retention of Pd(0).

Control reactions were performed using 4-iodotoluene and 4-bromotoluene as representative substrates under otherwise identical conditions to evaluate the contributions of the cage scaffold and nitrogen-donor functionality (Table S3). No detectable product was observed in the absence of catalyst or with the metal-free BPY-OMC and PHEN-OMC cages. Pd(OAc)_2_ afforded 66% and 64% conversion for the representative aryl iodide and aryl bromide substrates, respectively, consistent with previous reports.^77,78^ Unsupported Pd-NPs afforded 40% and 26% conversion, while Pd-NP@QP-OMC afforded 45% and 30% conversion, respectively. In contrast, both Pd-NP@BPY-OMC and Pd-NP@PHEN-OMC afforded >99% conversion for both substrates. These results indicate that the combination of nitrogen-donor sites and the cage scaffold substantially improves catalytic performance under the aqueous reaction conditions.

The heterogeneity and stability of the Pd-NP@OMC catalysts were evaluated by hot filtration and recycling studies. Hot filtration experiments were performed by heating the Pd-NP@OMC catalysts under the reaction conditions in the absence of substrate, followed by removal of the catalyst and addition of the coupling substrates to the filtrate. Negligible conversion was observed, indicating minimal Pd leaching under these conditions and supporting a heterogeneous catalytic mechanism (Fig. S8). Catalyst recyclability was assessed over five reaction cycles (Fig. 3B). The first two cycles were conducted consecutively within the same day following catalyst drying, whereas the remaining cycles were performed after the catalysts had been stored in air for 10–14 days. Both OMC-supported catalysts maintained high conversions without a significant loss in activity, whereas unsupported Pd-NPs showed rapid deactivation. Although these Pd-NPs are not expected to exhibit meaningful recyclability, the residual activity likely arises from partial stabilization by TBAB present in the reaction mixture.^54^ After storage in air for 10–14 days, the cage-free Pd-NPs exhibited essentially no catalytic activity (Fig. 3B). In contrast, both OMC-supported catalysts retained their activity after 10–14 days of storage in air, demonstrating the stabilizing effect of the cage support. This stability contrasts with many commercial Pd(0) catalysts, such as Pd(PPh_3_)_4_, which typically require storage under an inert atmosphere.^60^^1^H NMR analysis of the recycled catalysts indicated the cage frameworks remained intact under catalytic conditions (Fig. S9). Hot filtration tests on the recycled material indicated minimal Pd leaching into the solution phase, further supporting the stability of the cage-supported Pd-NPs (Fig. S8). X-ray photoelectron spectroscopy (XPS) analysis of the recycled materials revealed no significant change in oxidation state (Fig. 3C and Fig. S6E, F), consistent with stabilization of Pd(0) by the cage support under catalytic conditions and during storage in air. TEM analysis showed retention of the overall NP morphology after recycling, with some increase in average particle size, particularly for Pd-NP@BPY-OMC, but without extensive aggregation, further demonstrating the stabilizing effect of the cage support (Fig. S10).

In summary, spiroborate-linked OMCs functionalized with BPY and PHEN donor sites coordinate Pd precursors and stabilize Pd-NPs at the cage periphery. The more rigid PHEN-OMC produced smaller Pd-NPs with a narrower size distribution than the BPY-OMC, illustrating how cage-bound ligand geometry influences NP formation. The Pd-NP@OMC materials retained high catalytic activity over five reaction cycles after storage in air, demonstrating dynamically assembled ionic OMCs as tunable supports for aqueous nanoparticle catalysis.

## Conflicts of interest

There are no conflicts to declare.

## Supplementary Material

CC-062-D6CC03901D-s001

CC-062-D6CC03901D-s002

## Data Availability

All data needed to support the conclusions in the paper are present in the paper and the supplementary information (SI). Supplementary information: materials and general methods, synthetic procedures, computational modeling of the OMCs, preparation of cage-supported nanoparticles, ICP analysis, hot filtration tests, TEM of recycled catalysts, FT-IR spectra, melting-point/decomposition observations, NMR spectra of cage precursors and OMC compounds, and NMR spectra of extracted catalysis products. See DOI: https://doi.org/10.1039/d6cc03901d. CCDC 2565457 contains the supplementary crystallographic data for this paper.^79^
