# Supplementary material for: Ionic organic molecular cage-supported Pd nanoparticles for aqueous Suzuki–Miyaura coupling
Source: Chem Commun (Camb). 2026 Sep 21;62(82):20665–9. doi: 10.1039/d6cc03901d (PMC13613970; doi:10.1039/d6cc03901d)
Supplement: Supplementary file 1 [file CC-062-D6CC03901D-s001.pdf]

Supplementary Information (SI) for ChemComm.
This journal is © The Royal Society of Chemistry 2026

Supporting Information

Ionic organic molecular cage-supported Pd nanoparticles for aqueous Suzuki-Miyaura coupling

Brandon Bishop[a], Xiaoyu Fang[a], Simon Teat[c], Zhehao Yuan[a], Shuyan Jiang[a], Alec Kolodziejczyk[a], Qiucheng Xu[a], and Wei Zhang[*a,b]

[a] *Department of Chemistry, University of Colorado Boulder, Boulder, CO 80309, USA*

[b] *Physical Sciences School, Great Bay University, Songshan Lake Park, Dongguan 523000, P.R.China*

[c] *Advanced Light Source, Department of Chemistry, Lawrence Berkeley National Laboratory, University of California, Berkeley, CA 94720, USA.*

[*] *Corresponding author: wei.zhang@colorado.edu*

Table of Contents

1.	Materials and General Methods	3
2.	Synthetic Procedures	3
3.	Structural Characterization of the OMCs	6
4.	Computational Modeling of the OMCs	7
5.	Preparation of Cage-Supported Nanoparticles	7
6.	ICP Analysis	10
7.	Substrate Table	11
8.	Hot Filtration Tests	11
9.	^{1}H NMR Spectra of Recycled Pd-NP@OMC Catalysts	12
10.	TEM of Recycled Catalyst	13
11.	NMR Spectra of Cage Precursors and OMC Compounds	13
12.	NMR Spectra of Extracted Catalysis Products	23
13.	References	28

1. Materials and General Methods

Reagents and solvents were purchased from commercial suppliers and used without further purification, unless otherwise indicated. Dimethylformamide (DMF) was purified by the MBRAUN solvent purification system. Solvents were evaporated using a rotary evaporator after workup. Unless otherwise specified, the purity of the compounds was ≥ 95% based on ^{1}H NMR spectral integration. Flash column chromatography was performed by using a 100–150 times weight excess of flash silica gel 32–63 µm from Dynamic Absorbents Inc. Fractions were analyzed by TLC using TLC silica gel F254 250 µm precoated-plates from Dynamic Absorbents Inc.

NMR spectra were recorded on Bruker 300 MHz, INOVA 400 and 500 MHz spectrometers. $CHCl_3$ (7.26 ppm) was used as an internal reference for ^{1}H NMR, and $CDCl_3$ (77.16 ppm) was used for ^{13}C NMR. ^{1}H NMR data were reported in the following order: chemical shift, multiplicity (s, singlet; d, doublet; t, triplet; q, quartet; m, multiplet), coupling constants (*J*, Hz), number of protons. The single-crystal X-ray diffraction data were collected at Beamline 12.2.1 of the Advanced Light Source (ALS) at Lawrence Berkeley National Laboratory (LBNL) using radiation with λ = 0.72880 Å at 100 K. The high-resolution mass spectra were obtained on Waters SYNAPT G2 High-Definition Mass Spectrometry System. Analyte molecules were diluted into methanol, chloroform, or acetonitrile/water mixture for final concentrations of 10 ppm or lower. The solution was injected into the electrospray ionization (ESI) source at a rate of 5 µL/min. Either ESI^{+} or ESI^{-} mode was used based on the molecular properties. Accurate mass analysis was performed by using the Lock Mass calibration feature of the instrument. The Fourier-transform infrared (FT-IR) spectra were obtained from an Agilent Technologies Cary 630 FT-IR spectrometer. Ultraviolet–visible spectroscopy (UV-vis) was measured on Agilent Cary 5000 UV-vis-NIR. Melting points and decomposition behavior were evaluated using a DigiMelt SRS MPA160 melting-point apparatus and are uncorrected. Samples that showed no melting or visible decomposition below the instrument limit are reported as >260 °C.

All electronic structure calculations were carried out using the CP2K program package (version 2023.1) employing the Gaussian and plane wave (GPW) method.[1,2] Density functional theory (DFT) calculations were performed using the Perdew–Burke–Ernzerhof (PBE) exchange–correlation functional within the generalized gradient approximation (GGA).[3] Long-range dispersion interactions were accounted for using the DFT-D3 correction with Becke–Johnson damping (D3(BJ)).[4]

Core electrons were described using Goedecker–Teter–Hutter (GTH) pseudopotentials,[5] and the valence electronic wave functions were expanded in DZVP-MOLOPT-SR-GTH Gaussian basis sets.[6] An auxiliary plane-wave basis with a cutoff energy of 300 Ry and a relative cutoff of 40 Ry was employed for the electronic density.

All calculations were performed under nonperiodic boundary conditions using a cubic simulation cell of $60 \times 60 \times 60$ Å^3, which is sufficiently large to avoid spurious interactions between periodic images. The orbital transformation (OT) method was used to achieve self-consistent field (SCF) convergence, with a convergence threshold of 3.0×10^{-5} Ha for the electronic energy.

The Pd contents in the catalysts were quantified using a Thermo Scientific iCAP PRO X inductively coupled plasma optical emission spectrometer (ICP-OES). The instrument was run with a pump rate of 100 r/min and a nebulizer flow of 28.0 psi. A split-range integration strategy was used with 5 seconds for low wavelengths (Low WL) and 10 seconds for high wavelengths (High WL) to optimize sensitivity, reduce detector noise, and manage spectral interferences across the UV-vis spectrum. The auxiliary argon gas flow was 0.5 L/min. The sample was flushed for 20 s and the radiofrequency power was 1150 W.

X-ray photoelectron spectroscopy (XPS) analysis was conducted on an ESCALAB 250Xi spectrometer equipped with a monochromatized Al Kα excitation source (Thermo Fisher). XPS spectra were acquired using 10 scans. XPS peak fitting was performed using the Advantage software (Thermo Scientific) and all the acquired XPS data were calibrated according to the C1s neutral carbon peak at 284.8 eV. The bright-field transmission electron microscopy (TEM) images were captured by a JEM-F200 instrument (JEOL Ltd., Japan), operating at an accelerating voltage of 200 kV.

2. Synthetic Procedures

2,3,6,7,10,11-hexahydroxytriphenylene (HHTP), 2,3-dimethoxyphenyl boronic acid, and the quaterphenyl-based sidearm (2,3,2′′′,3′′′-tetrahydroxy-[1,1′:4′,1′′:4′′,1′′′-quaterphenyl], (QP-4OH)) were synthesized following previously reported procedures without modification.[7-9]

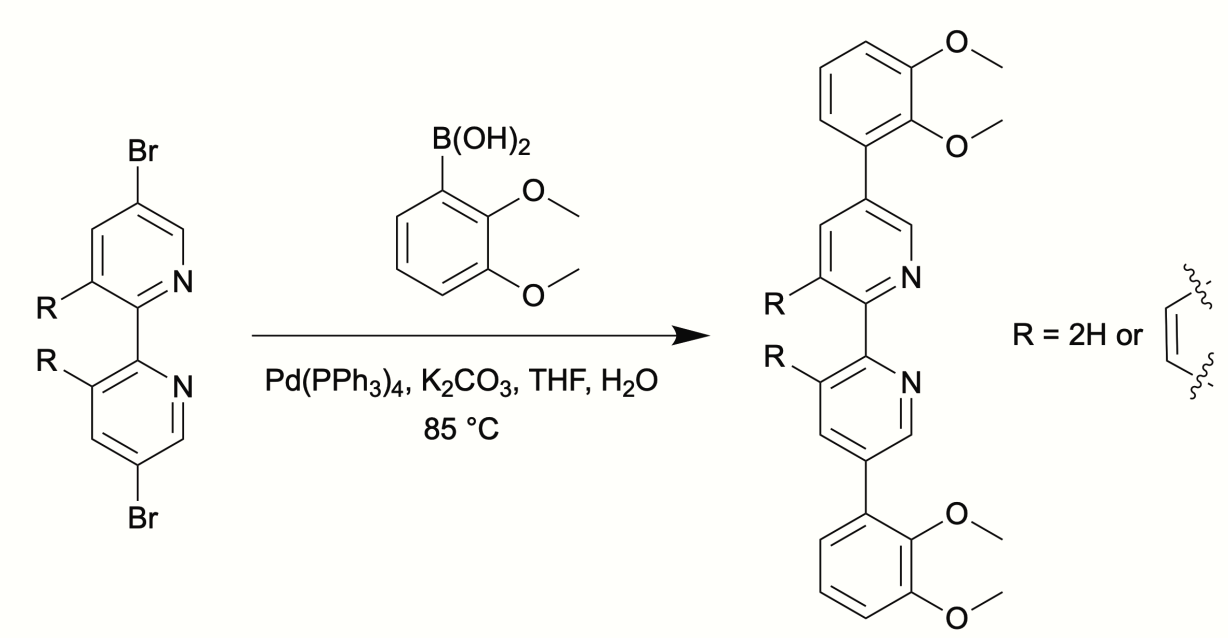

Synthesis of 5,5′-bis(2,3-dimethoxyphenyl)-2,2′-bipyridine (BPY-4OMe) and 3,8-bis(2,3-dimethoxyphenyl)-1,10-phenanthroline (PHEN-4OMe): 5,5′-dibromo-2,2′-bipyridine (706 mg, 2.25 mmol) or 3,8-dibromo-1,10-phenanthroline (760.03 mg, 2.25 mmol) were added to a Schlenk tube. 2,3-dimethoxyphenyl boronic acid (896 mg, 4.95 mmol) was then added, followed by potassium carbonate (K_2CO_3; 931 mg, 6.75 mmol) and tetrakis(triphenylphosphine)palladium(0) ($Pd(PPh_3)_4$; 260 mg, 0.225 mmol). A solvent mixture of THF:H_2O (5:1, 20 mL) was bubbled with argon (Ar) for 5-10 min and then added to the Schlenk tube. The reaction mixture was allowed to stir at 85 °C for 18 h under an Ar atmosphere. After removing the solvent *in vacuo*, water and dichloromethane (CH_2Cl_2) were added to extract the product. The organic layer was dried over anhydrous Na_2SO_4. The solvent was removed *in vacuo* and the residue was purified by flash chromatography on silica gel (BPY-4OMe: gradient, pure CH_2Cl_2, then 2:1 ethyl acetate: hexanes; PHEN-4OMe: gradient, ethyl acetate: hexanes 1:2, then pure ethyl acetate). The products were obtained as white powders (BPY-4OMe: 819 mg, 85%; PHEN-4OMe: 849 mg, 83%). BPY-4OMe: ^1H NMR (400 MHz, $CDCl_3$) δ 8.91 (dd, *J* = 2.3, 0.9 Hz, 2H), 8.49 (dd, *J* = 8.2, 0.9 Hz, 2H), 8.06 (dd, *J* = 8.2, 2.3 Hz, 2H), 7.18 (t, *J* = 8.0 Hz, 2H), 7.01 (ddd, *J* = 17.6, 8.0, 1.5 Hz, 4H), 3.93 (s, 6H), 3.65 (s, 6H). ^{13}C NMR (75 MHz, $CDCl_3$) δ 154.71, 153.42, 149.63, 146.92, 137.73, 133.93, 132.41, 124.63, 122.27, 120.58, 112.53, 60.87, 56.09. HR-ESI (*m/z*): $[M+H]^+$ calcd. for $C_{26}H_{24}N_2O_4$, 429.1786; found: 429.1816. PHEN-4OMe: ^1H NMR (300 MHz, $CDCl_3$) δ 9.43, 9.42, 8.45, 8.44, 7.87, 7.24, 7.23, 7.21, 7.20, 7.16, 7.16, 7.14, 7.13, 7.06, 7.05, 7.03, 3.97, 3.65, 3.65, 3.64. ^{13}C NMR (75 MHz, $CDCl_3$) δ 153.49, 151.83, 151.36, 137.60, 135.96, 133.80, 129.82, 128.42, 125.66, 124.83, 122.56, 119.88, 112.90, 61.01, 56.13. HR-ESI (*m/z*): $[M+H]^+$ calcd. for $C_{28}H_{24}N_2O_4$, 453.1766; found: 453.1764.

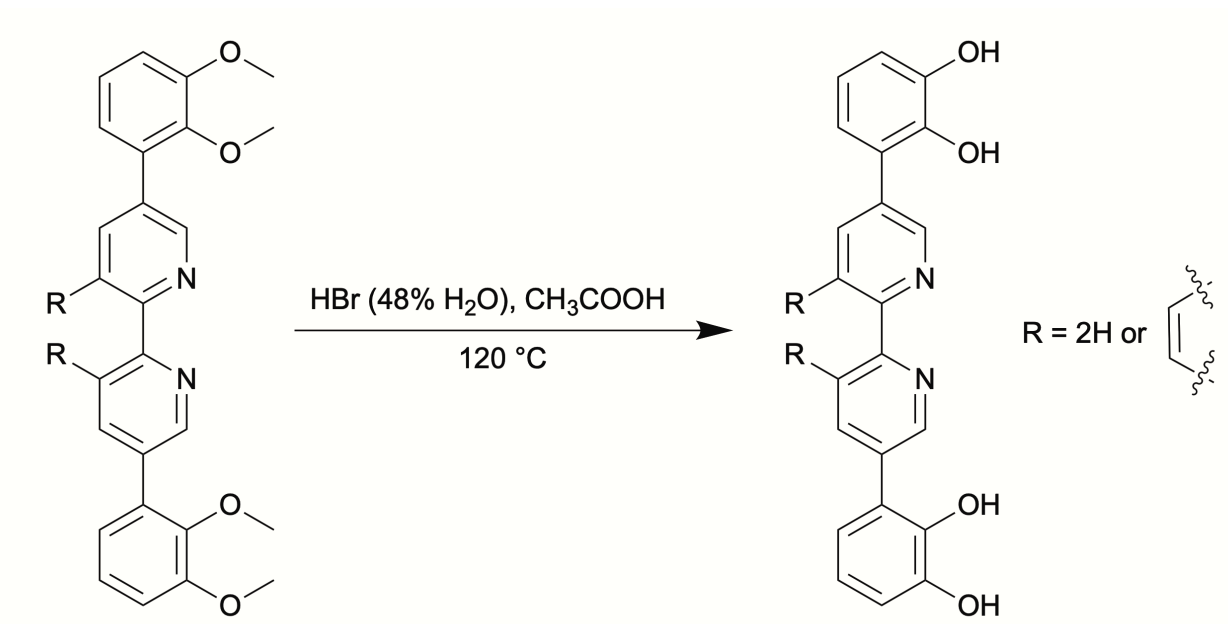

Synthesis of 5,5′-bis(2,3-dihydroxyphenyl)-2,2′-bipyridine (BPY-4OH) and 3,8-bis(2,3-dihydroxyphenyl)-1,10-phenanthroline (PHEN-4OH): BPY-4OMe (819 mg, 1.91 mmol) or PHEN-4OMe (849 mg, 1.88 mmol) was added to a dry three-neck round-bottom flask under an Ar atmosphere. It was then suspended in 1:1 solution of HBr (48% in water) and acetic acid (50 mL total). The suspension was refluxed for 24 h. Then it was cooled, and the product was filtered off as yellow (BPY-4OH) or red-brown (PHEN-4OH) solid that was washed with an excess amount of water and dried under vacuum. (BPY-4OH: 669 mg, 94%; PHEN-4OH: 693 mg, 93%) BPY-4OH: ^1H NMR (500 MHz, DMSO) δ 9.02 (s, 1H), 8.66 (d, *J* = 8.5 Hz, 1H), 8.49 (d, *J* = 8.5 Hz, 1H), 6.96 (d, *J* = 7.8 Hz, 2H), 6.80 (t, *J* = 7.8 Hz, 1H). ^{13}C NMR (75 MHz, DMSO) δ 146.55, 146.32, 145.96, 143.37, 141.14, 136.68, 122.64, 122.56, 120.31, 119.92, 116.32. HR-ESI (*m/z*): $[M+H]^+$ calcd. for $C_{22}H_{16}N_2O_4$, 373.1163; found: 373.1188. FT-IR (ATR, cm^{-1}): 3384, 3082, 1591, 1546, 1464, 1271, 1159, 771, 719. mp 226 °C (dec.). PHEN-4OH: ^1H NMR (500 MHz, DMSO) δ 9.86 (s, 2H), 9.33 (s, 2H), 8.92 (s, 2H), 8.14 (s, 2H), 6.99 (d, *J* = 7.6 Hz, 4H), 6.83 (t, *J* = 7.8 Hz, 2H). ^{13}C NMR (75 MHz, DMSO) δ 148.53, 146.00, 143.33, 138.73, 137.52, 135.09, 128.88, 127.48, 123.13, 120.75, 119.99, 116.27. HR-ESI (*m/z*): $[M+H]^+$ calcd. for $C_{24}H_{16}N_2O_4$, 397.1161; found: 397.1189. FT-IR (ATR, cm^{-1}): 3364, 3046, 1584, 1546, 1468, 1271, 1159, 775, 715. mp 237 °C (dec.).

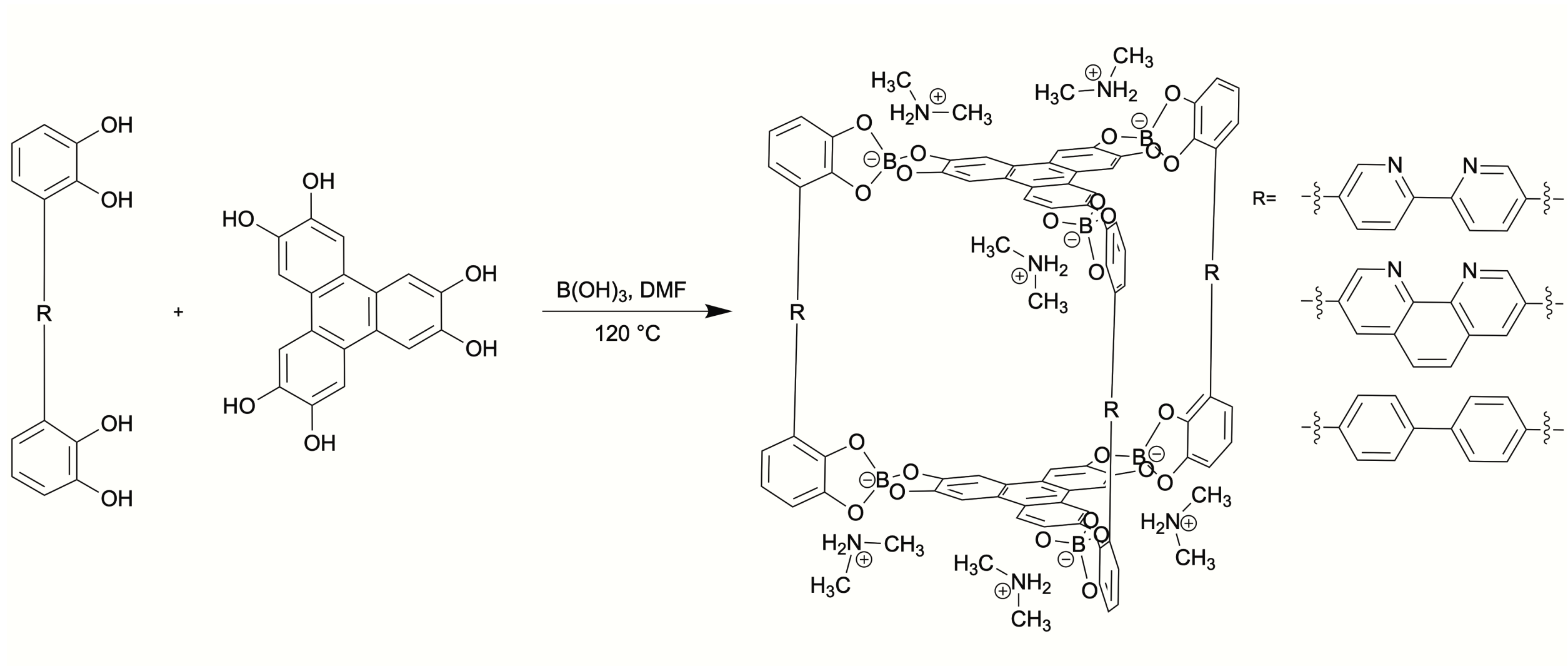

Synthesis of Control and Functionalized Spiroborate-Based Organic Molecular Cages (QP-OMC and BPY- and PHEN-OMCs): An ampule was charged with one of the three dicatechol-functionalized pillars (0.09 mmol; 33.5 mg BPY-4OH, 35.7 mg PHEN-4OH, 33.3 mg QP-4OH), HHTP (19.5 mg, 0.06 mmol), boric acid (11.12 mg, 0.18 mmol), and DMF (2 mL). The ampule was flame-sealed and heated at 120 °C for 18 h without any stirring or disturbance. After cooling to room temperature, the resulting OMC was precipitated and washed with diethyl ether. The centrifuged material was dried under high vacuum for 16 h to obtain the cage products as a grey powder (QP-OMC), a yellow-orange powder (BPY-OMC), or a dark red powder (PHEN-OMC). The recovered OMCs were further purified through vapor-diffusion recrystallization from a concentrated DMF solution using methoxycyclopentane. Single crystals were obtained for the QP-OMC. (QP-OMC: 56 mg, 90%, BPY-OMC: 55 mg, 88%; PHEN-OMC: 46 mg, 71%) QP-OMC: ^{1}H NMR (500 MHz, DMSO) δ 8.09 – 8.00 (m, 2H), 7.75 – 7.71 (m, 2H), 7.48 (s, 2H), 6.97 (dd, *J* = 8.3, 1.4 Hz, 1H), 6.65 (t, *J* = 7.8 Hz, 1H), 6.56 (dd, *J* = 7.4, 1.3 Hz, 1H), 2.45 (s, 8H). ^{13}C NMR (75 MHz, DMSO) δ 152.27, 151.08, 149.36, 137.08, 136.87, 128.05, 127.84, 126.17, 125.91, 121.88, 119.22, 117.92, 115.96, 107.27, 99.74. ^{11}B NMR (96 MHz, DMSO) δ 14.13. HR-ESI (*m/z*): $[M-4H]^{4-}$ calcd. for $C_{108}H_{50}B_6O_{24}^{4-}$, 449.0899; found: 449.1011. FT-IR (ATR, cm^{-1}): 3034, 2784, 1073, 1051, 946, 851, 793. mp >260 °C. BPY-OMC: ^{1}H NMR (500 MHz, DMSO) δ 9.26 (s, 1H), 8.48 (s, 2H), 8.13 (s, 3H), 7.49 (s, 2H), 7.04 (d, *J* = 8.2 Hz, 1H), 6.71 (t, *J* = 7.8 Hz, 1H), 6.63 (d, *J* = 7.4 Hz, 1H), 2.48 (t, *J* = 5.7 Hz, 10H). ^{13}C NMR (75 MHz, DMSO) δ 152.26, 151.01, 149.73, 147.74, 135.60, 133.81, 121.91, 120.00, 118.36, 116.24, 115.90, 108.08, 99.83, 34.29. ^{11}B NMR (96 MHz, DMSO) δ 14.04. HR-ESI (*m/z*): $[M-3H]^{3-}$ calcd. for $C_{102}H_{51}B_6N_6O_{24}^{3-}$, 603.1150; found: 603.1193. FT-IR (ATR, cm^{-1}): 3026, 2765, 1610, 1490, 1444, 1364, 1237, 1151, 1039, 935, 838. mp >260 °C. PHEN-OMC: ^{1}H NMR (300 MHz, DMSO) δ 9.69 (s, 1H), 8.97 (s, 1H), 8.41 (s, 2H), 8.07 (s, 1H), 7.55 (s, 2H), 7.19 (d, *J* = 8.0 Hz, 1H), 6.80 (t, *J* = 7.8 Hz, 1H), 6.71 (d, *J* = 7.4 Hz, 1H), 2.50 (s, 2H). ^{13}C NMR (75 MHz, DMSO) δ 152.35, 151.05, 149.94, 149.32, 142.46, 134.09, 133.29, 128.38, 127.27, 121.92, 118.51, 116.20, 108.35, 99.93, 34.35. ^{11}B NMR (96 MHz, DMSO) δ 14.29. HR-ESI (*m/z*): $[M-4H]^{4-}$ calcd. for $C_{108}H_{50}B_6N_6O_{24}^{4-}$, 469.5828; found: 469.5836. FT-IR (ATR, cm^{-1}): 3019, 2761, 1606, 1490, 1453, 1367, 1244, 1151, 1039, 902, 831. mp >260 °C.

General procedure for Suzuki–Miyaura Coupling Reaction: To a 10 mL Schlenk tube was added: aryl halide (0.5 mmol), phenylboronic acid (0.55 mmol), TBAB (0.25 mmol), Na_2CO_3 (0.75 mmol), and Pd catalyst corresponding to 0.5 mol% Pd. H_2O (1.5 mL) was added in air and the reaction was heated at 90 °C or 120 °C for 3-5 h. The progress of the reaction was monitored by TLC analysis. Afterwards, the reaction mixture was diluted with water (4 mL), centrifuged and washed with water and hexanes (3 x 4 mL) to recover the catalyst. The supernatants were combined, extracted and separated in a separatory funnel. The organic layer was collected and dried over Na_2SO_4, and the solvent was evaporated by using a rotary evaporator. The recovered catalyst was reused after washing with CH_2Cl_2 and methanol followed by drying under high vacuum for 12 h. The extracted catalysis product was analyzed by ^{1}H NMR.

Microwave procedure for aqueous Suzuki-Miyaura coupling: To a 10 mL microwave synthesizer reaction vessel was added: aryl halide (0.5 mmol), phenylboronic acid (0.55 mmol), TBAB (0.25 mmol), Na_2CO_3 (0.75 mmol), and Pd catalyst corresponding to 0.5 mol% Pd. H_2O (1.5 mL) was added. The vessel was sealed using a silicone cap with a septum and further secured using a piece of tightly wrapped electrical tape. The reaction mixture was heated in a microwave reactor using the following conditions: 30 min, 90 °C or 120 °C, 130 PSI, 150 W. The reaction mixture was diluted with water (4 mL), centrifuged and washed with water and

hexanes (3 x 4 mL) to recover the catalyst. The supernatants were combined, extracted and separated in a separatory funnel. The organic layer was collected and dried over Na_2SO_4, and the solvent was evaporated by using a rotary evaporator. The recovered catalyst was reused after washing with CH_2Cl_2 and methanol followed by drying under high vacuum for 12 h. The extracted catalysis product was analyzed by ^1H NMR.

3. Structural Characterization of the OMCs

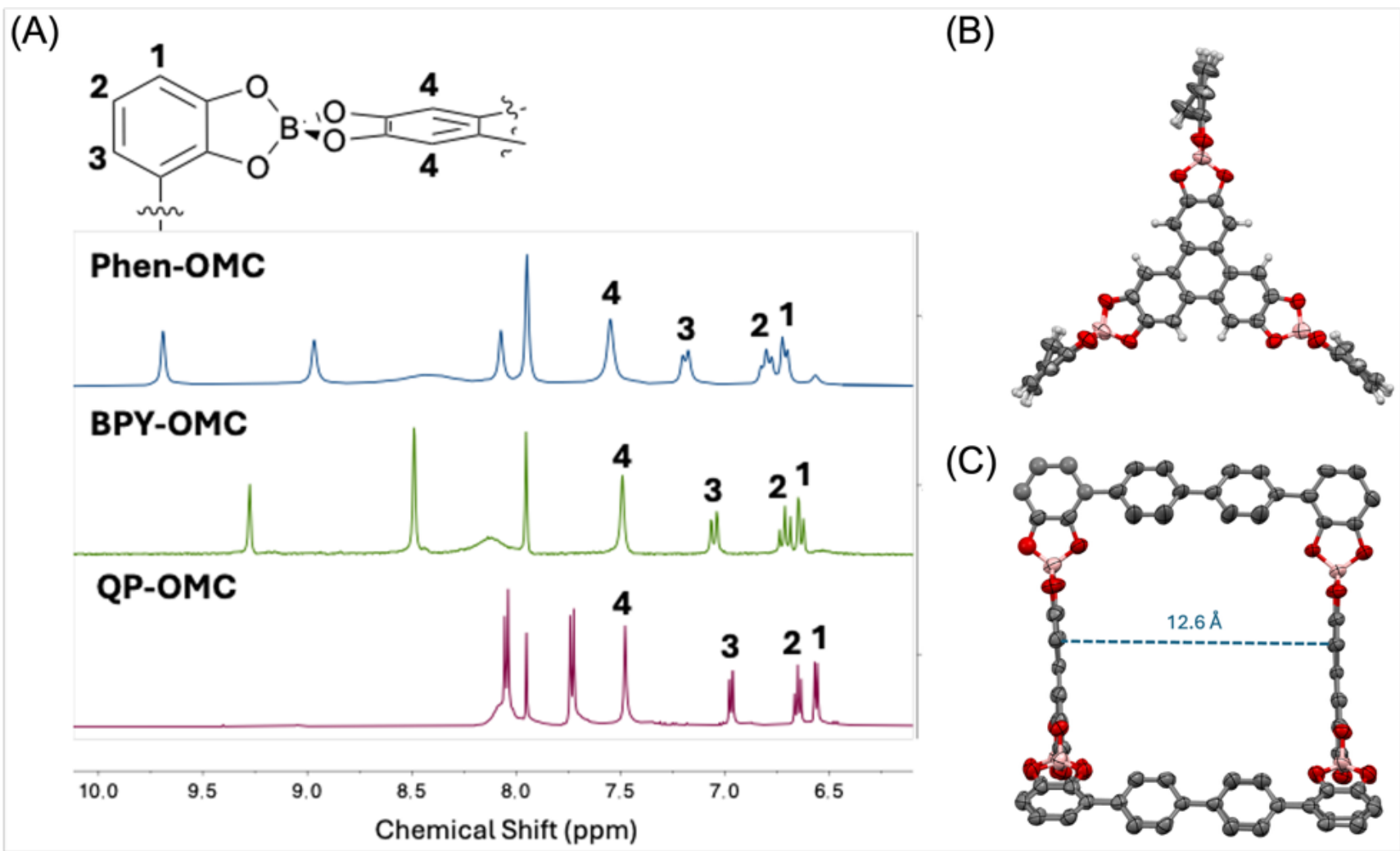

Fig. S1 Structural characterization of spiroborate-linked OMCs. (A) Stacked ^1H NMR spectra of QP-OMC, BPY-OMC, and PHEN-OMC with representative proton assignments. The characteristic doublet–triplet–doublet pattern corresponding to the cage corners and the singlet associated with the HHTP panels are labeled. The spectra differ primarily in the resonances corresponding to the central aromatic units of the sidearms. (B) Top view of the single-crystal X-ray structure of QP-OMC. (C) Side view of the single-crystal X-ray structure of QP-OMC showing an internal distance of 12.6 Å between the two HHTP panels. Thermal ellipsoids are shown at the 50% probability level. Hydrogen atoms, counterions, and solvent molecules are omitted for clarity.

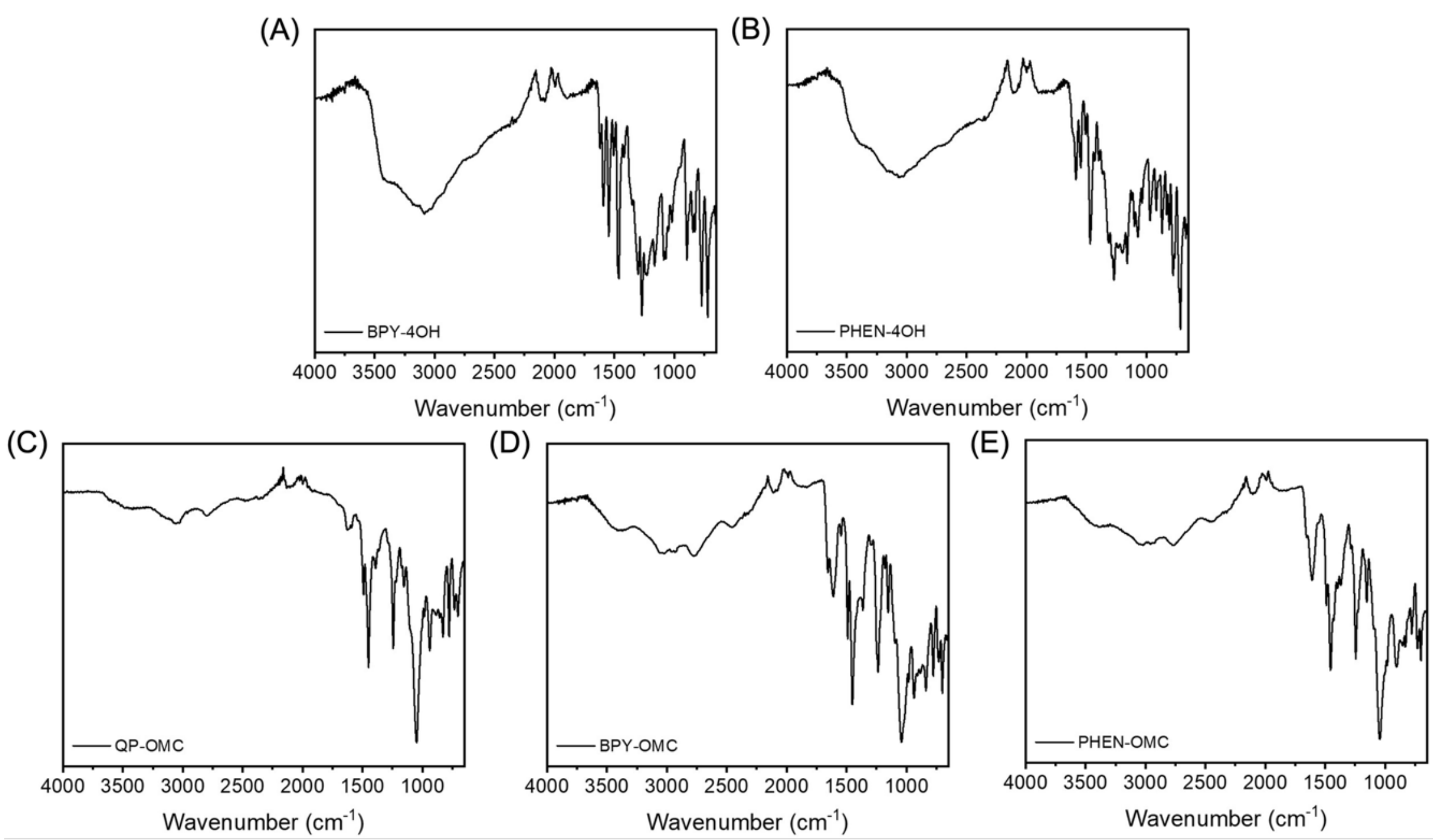

Fig. S2 FT-IR spectra of the newly synthesized tetrahydroxy cage precursors and spiroborate-linked OMCs. (A) BPY-4OH, (B) PHEN-4OH, (C) QP-OMC, (D) BPY-OMC, and (E) PHEN-OMC.

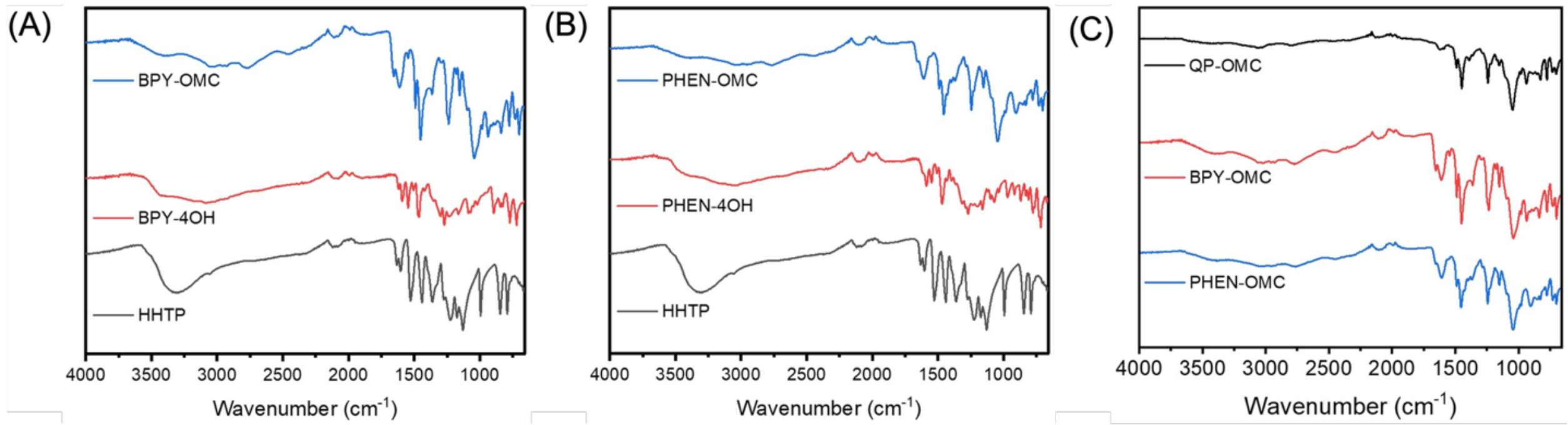

Fig. S3 Comparative FT-IR spectra of the cage precursors and corresponding spiroborate-linked OMCs. (A) BPY-4OH, HHTP, and BPY-OMC; (B) PHEN-4OH, HHTP, and PHEN-OMC; and (C) QP-OMC, BPY-OMC, and PHEN-OMC. Spectra were normalized and overlaid for comparison.

4. Computational Modeling of the OMCs

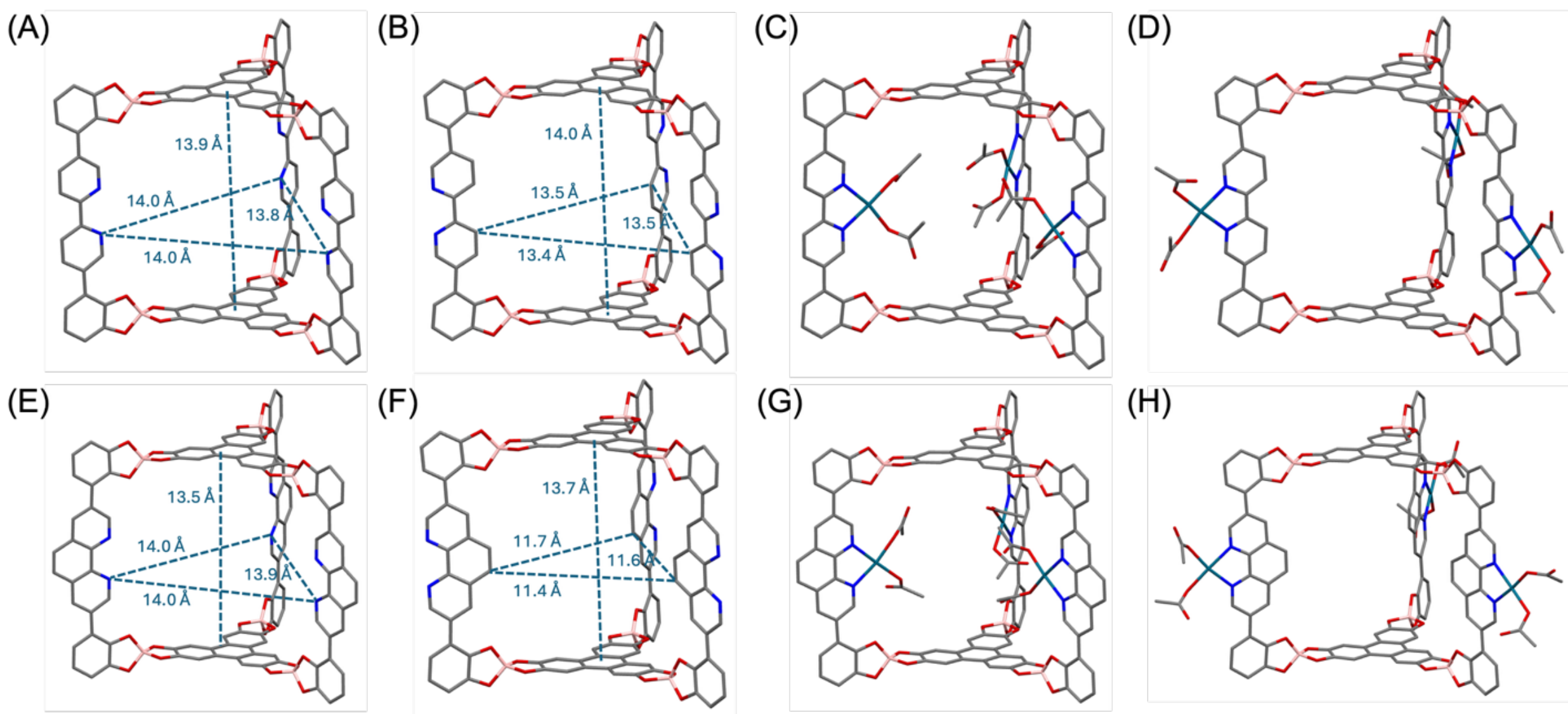

Fig. S4 CP2K computational modeling of the energy-minimized structures of the BPY-OMC and the PHEN-OMC with and without Pd coordination, analyzing the thermodynamically favored nitrogen orientation. (A) BPY-OMC with nitrogens oriented inward toward the cage cavity. (B) BPY-OMC with nitrogens oriented outward toward the cage periphery. (C) Pd@BPY-OMC with nitrogens oriented inward toward the cage cavity. (D) Pd@BPY-OMC with nitrogens oriented outward toward the cage periphery. (E) PHEN-OMC with nitrogens oriented inward toward the cage cavity. (F) PHEN-OMC with nitrogens oriented outward toward the cage periphery. (G) Pd@PHEN-OMC with nitrogens oriented inward toward the cage cavity. (H) Pd@PHEN-OMC with nitrogens oriented outward toward the cage periphery.

Nitrogen Orientation (energy (a.u.))	BPY-OMC	PHEN-OMC	Pd@BPY-OMC	Pd@PHEN-OMC
In	-1227.393127	-1261.545650	-1880.534209	-1914.682929
Out	-1227.375212	-1261.599512	−1880.569829	-1914.771418

Table S1 Minimized energy values obtained from the CP2K computational modeling of the BPY-OMC and PHEN-OMC based on inward (in) or outward (out) orientation of the nitrogens in the bipyridine or phenanthroline motifs with or without Pd coordination.

5. Preparation of Cage-Supported Nanoparticles

General procedure for the growth of cage-supported Pd-NPs: BPY-OMC (10 mg, 4.81 μmol) or PHEN-OMC (10 mg, 4.64 μmol) was dissolved in dimethylacetamide (DMAc)(1 mL). A solution of $Pd(OAc)_2$ (3 eq) in DMAc (0.5 mL) was added and stirred overnight at 25 °C. A noticeable color change occurred indicating coordination. Then NaBH4 (6.0 equivalents to $Pd(OAc)_2$) in methanol (0.75 mL) was added dropwise into the reaction mixture at 25 °C. After stirring for 3 h, the Pd-NP@BPY-OMC or Pd-NP@PHEN-OMC material was precipitated using diethyl ether. The Pd-NP@OMC materials were dried under vacuum at 25 °C overnight.

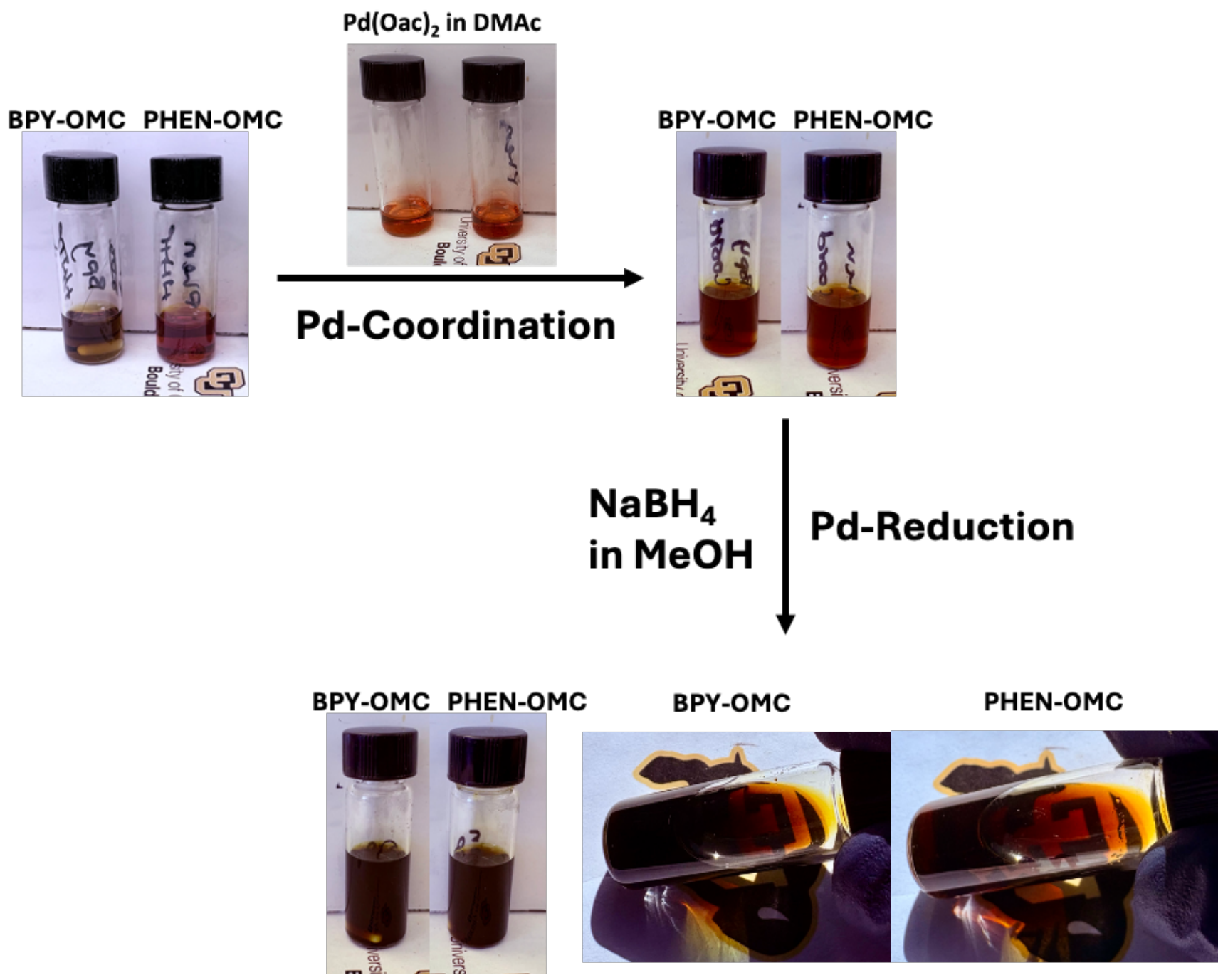

Fig. S5 Real-time photographs of solutions containing BPY-OMC and PHEN-OMC during Pd-NP growth, showing color changes associated with Pd coordination, reduction, and stabilization by BPY-OMC or PHEN-OMC.

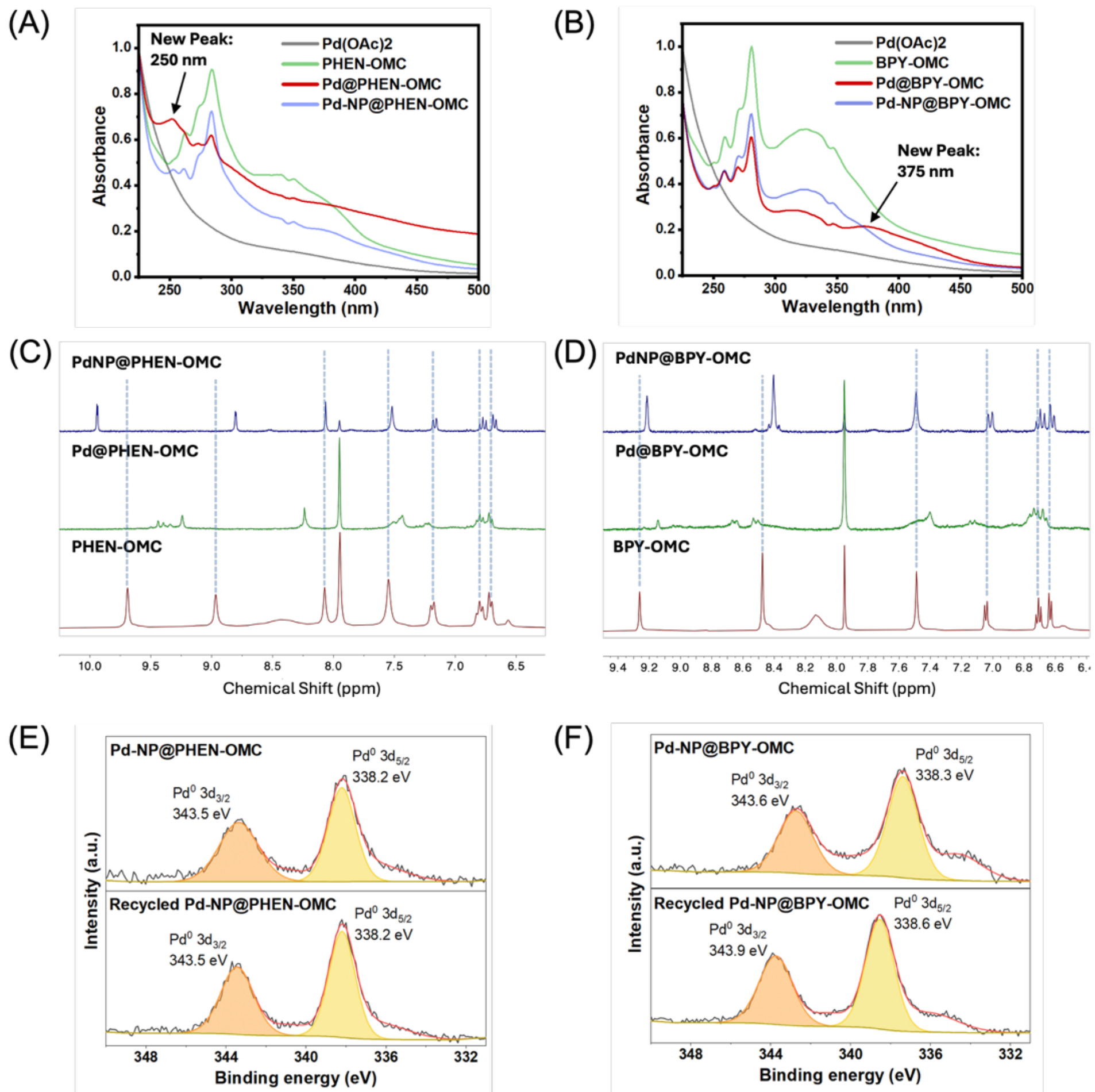

Fig. S6 UV-vis analysis, ^{1}H NMR analysis, and XPS analysis of Pd-NP@OMC materials. (A-B) UV-vis analysis of Pd-NP growth using PHEN-OMC and BPY-OMC as supports. (A) UV-vis analysis of Pd-NP growth using PHEN-OMC. (black curve: $Pd(OAc)_2$, green curve: PHEN-OMC, red curve: Pd@PHEN-OMC, blue curve: Pd-NP@PHEN-OMC) (B) UV-vis analysis of Pd-NP growth using BPY-OMC. (black curve: $Pd(OAc)_2$, green curve: BPY-OMC, red curve: Pd@BPY-OMC, blue curve: Pd-NP@BPY-OMC) (C-D) ^{1}H NMR spectra of the original BPY-OMC and PHEN-OMC compared to the Pd-coordinated OMCs and Pd-NP@OMCs. (C) BPY-OMC (bottom spectrum), Pd@BPY-OMC (middle spectrum), and Pd-NP@BPY-OMC (top spectrum). (D) PHEN-OMC (bottom spectrum), Pd@PHEN-OMC (middle spectrum), and Pd-NP@PHEN-OMC (top spectrum). (E-F) XPS analysis of original and recycled Pd-NP@PHEN-OMC and Pd-NP@BPY-OMC catalysts. (E) XPS analysis of original Pd-NP@PHEN-OMC (top curve) and recycled Pd-NP@PHEN-OMC (bottom curve). (F) XPS analysis of original Pd-NP@BPY-OMC (top curve) and recycled Pd-NP@BPY-OMC (bottom curve).

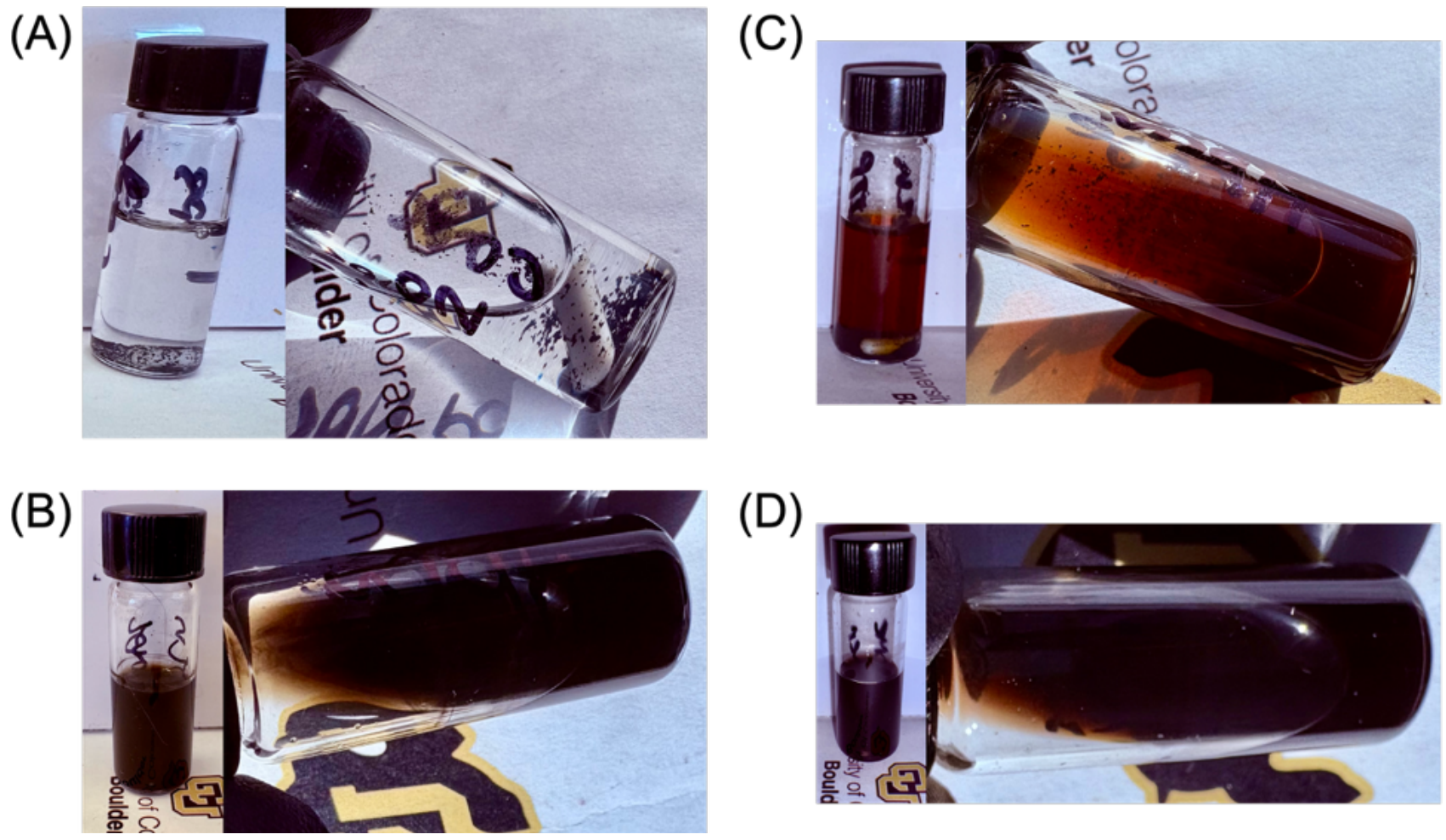

Fig. S7 Images depicting Pd-NP solutions from control experiments obtained after following the procedure for Pd-NP growth. Aggregation and precipitation can be seen in all images. (A) $Pd(OAc)_2$ alone. (B) QP-OMC. (C) BPY-4OH sidearm. (D) PHEN-4OH sidearm.

6. ICP Analysis

Sample ID	Sample mass m_0 (g)	Final volume V_0 (mL)	Element	C_o (mg/L)	f	C_1 (mg/L)	C_x (mg/kg)	Element content W (%)
PHEN-HHTP-CAGE-1	0.0205	100	PD	20.871	1	20.871	101809.8	10.18%
PHEN-HHTP-CAGE-2	0.0191	100	PD	20.735	1	20.735	108560.2	10.86%
RECYCLED PHEN-HHTP-CAGE-1	0.0207	100	PD	20.371	1	20.371	98410.6	9.84%
RECYCLED PHEN-HHTP-CAGE-2	0.0203	100	PD	21.075	1	21.075	103817.7	10.38%
BPY-HHTP-CAGE-1	0.0184	100	PD	18.381	1	18.381	99896.7	9.99%
BPY-HHTP-CAGE-2	0.0189	100	PD	17.637	1	17.637	93317.5	9.33%
RECYCLED-BPY-HHTP-CAGE-1	0.0186	100	PD	17.888	1	17.888	96172.0	9.62%
RECYCLED-BPY-HHTP-CAGE-2	0.0112	100	PD	10.258	1	10.258	91589.3	9.16%

Table S2 ICP-OES analysis of Pd content in Pd-NP@PHEN-OMC and Pd-NP@BPY-OMC. Multiple trials are included to demonstrate the reproducibility of the measured Pd content. The difference in Pd content reflects the different molecular weights of the two cages.

7. Substrate Table

Table S3 Suzuki–Miyaura coupling of various aryl halides and phenylboronic acid derivatives using Pd-NP@BPY-OMC and Pd-NP@PHEN-OMC, with Pd-NP@QP-OMC, unsupported Pd-NPs, $Pd(OAc)_2$, metal-free OMCs, and no-catalyst controls.[a, b]

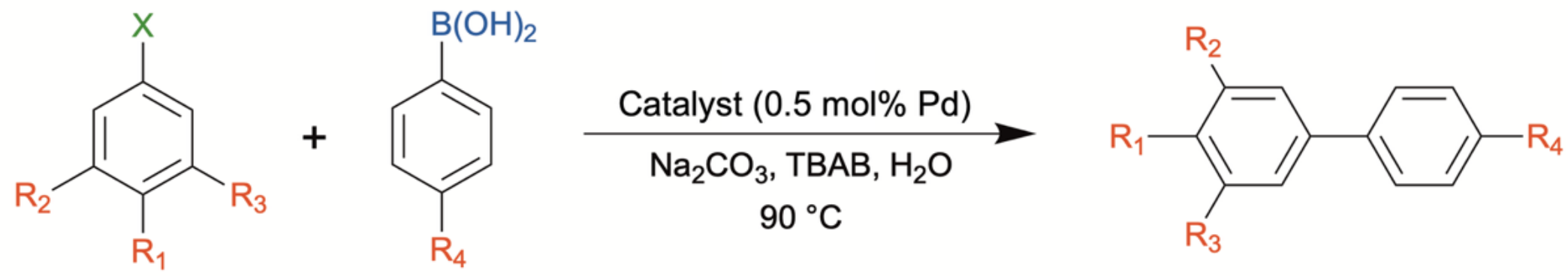

			Conversion (%)							
			Spiroborate-Linked OMCs			Controls				
Entry	Halobenzene (X, R_1-R_3)	Boronic Acid (R_4)	Pd-NP@ BPY-OMC	Pd-NP@ PHEN-OMC	Pd-NP@ QP-OMC	Pd-NP	$Pd(OAc)_2$	BPY-OMC	PHEN-OMC	No Catalyst
1	X= I, R_1= CH_3, R_2= H, R_3 = H	R_4 = H	>99% (>99%)	>99% (>99%)	45%	40%	66%	ND	ND	ND
2	X= I, R_1= H, R_2= H, R_3 = CN	R_4 = H	>99%	>99% (>99%)	-	-	-	-	-	-
3	X= Br, R_1= CH_3, R_2= H, R_3 = H	R_4 = H	>99% (>99%)	>99% (>99%)	30%	26%	64%	ND	ND	ND
4	X= Br, R_1= NO_2, R_2= H, R_3 = H	R_4 = H	>99%	>99% (>99%)	-	-	-	-	-	-
5	X= Br, R_1= H, R_2= CF_3, R_3 = CF_3	R_4 = H	>99%	>99% (>99%)	-	-	-	-	-	-
6	X= Br, R_1= CHO, R_2= H, R_3 = H	R_4 = H	>99%	>99% (>99%)	-	-	-	-	-	-
7	X= Br, R_1= OCH_3, R_2= H, R_3 = H	R_4 = H	93%*	>99%* (99%)#	-	-	-	-	-	-
8	X= Br, R_1= NH_2, R_2= H, R_3 = H	R_4 = H	99%*	>99%* (99%)#	-	-	-	-	-	-
9	X= Br, R_1= CH_3, R_2= H, R_3 = H	R_4 = CH_2CH_3	>99%	>99% (99%)	-	-	-	-	-	-
10	X= Br, R_1= CH_3, R_2= H, R_3 = H	R_4 = OCH_3	>99%	>99% (>99%)	-	-	-	-	-	-
11	X= Br, R_1= CH_3, R_2= H, R_3 = H	R_4 = CHO	94%*	98%* (96%)#	-	-	-	-	-	-

ND = no detectable product.
[a] Reaction conditions: halobenzene (0.5 mmol), boronic acid (0.55 mmol), Na_2CO_3 (0.75 mmol), tetrabutylammonium bromide (TBAB, 0.25 mmol), catalyst (0.5 mol % Pd for all Pd-containing entries), and H_2O (1.5 mL) in air at 90 °C for 3 h.
[b] Conversions were determined by ^1H NMR analysis after hexane extraction.
() Values in parentheses correspond to microwave-assisted reactions performed at 90 °C for 30 min at 150 W; conversions were determined by ^1H NMR analysis after hexane extraction.
* Reaction time = 5 h.
# Microwave reaction run at 120 °C
- = Not tested

8. Hot Filtration Tests

General procedure for hot filtration tests: To a 10 mL Schlenk tube was added: TBAB (0.25 mmol), Na_2CO_3 (0.75 mmol), and Pd catalyst corresponding to 0.5 mol% Pd. H_2O (1.5 mL) was added in air and the reaction was heated at 90 °C for 3 h. Afterwards,

the reaction was removed from the oil bath. The catalyst was removed by filtration using a syringe filter. The filtrate containing TBAB and Na_2CO_3 was added to a new Schlenk tube and the aryl halide (0.5 mmol) and phenylboronic acid (0.55 mmol) were added to the reaction mixture. The filtered reaction was then heated to 90 °C for an additional 3 h. Afterwards, the reaction mixture was diluted with water (4 mL) and extracted with hexanes (3 x 4 mL) in a separatory funnel. The organic layer was collected and dried over Na_2SO_4, and the solvent was evaporated by using a rotary evaporator. The extracted catalysis product was analyzed by ^1H NMR.

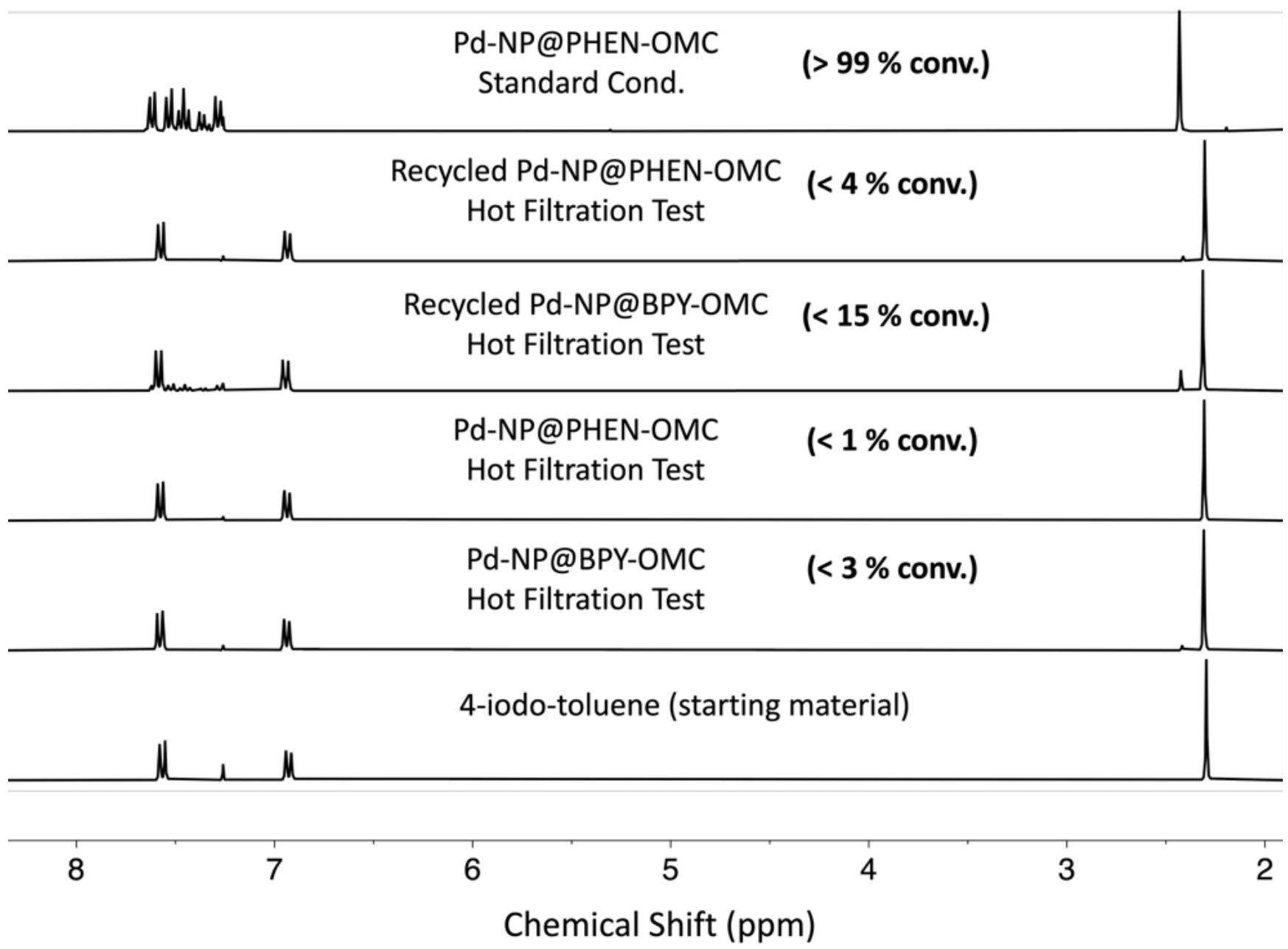

Fig. S8 ^1H NMR spectra obtained from the hot filtration tests for the reaction between 4-iodotoluene and phenylboronic acid performed in the presence of either Pd-NP@BPY-OMC or Pd-NP@PHEN-OMC. Both the original and recycled catalysts were examined.

9. ^1H NMR Spectra of Recycled Pd-NP@OMC Catalysts

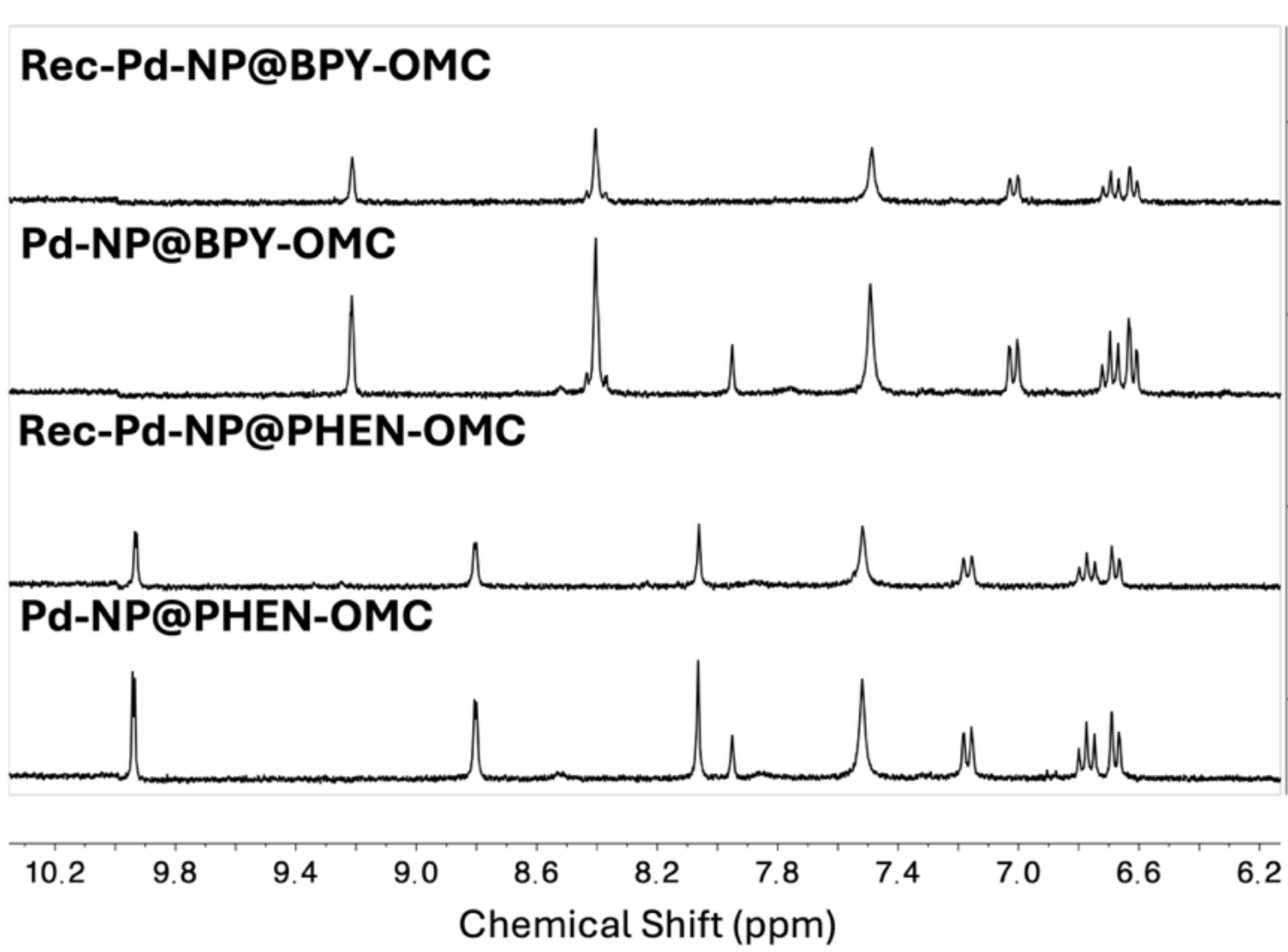

Fig. S9 Analysis of BPY-OMC and PHEN-OMC stability to catalytic conditions. ^1H NMR spectra of original and recycled BPY-OMC and PHEN-OMC demonstrating cage stability under catalytic conditions.

10. TEM of Recycled Catalyst

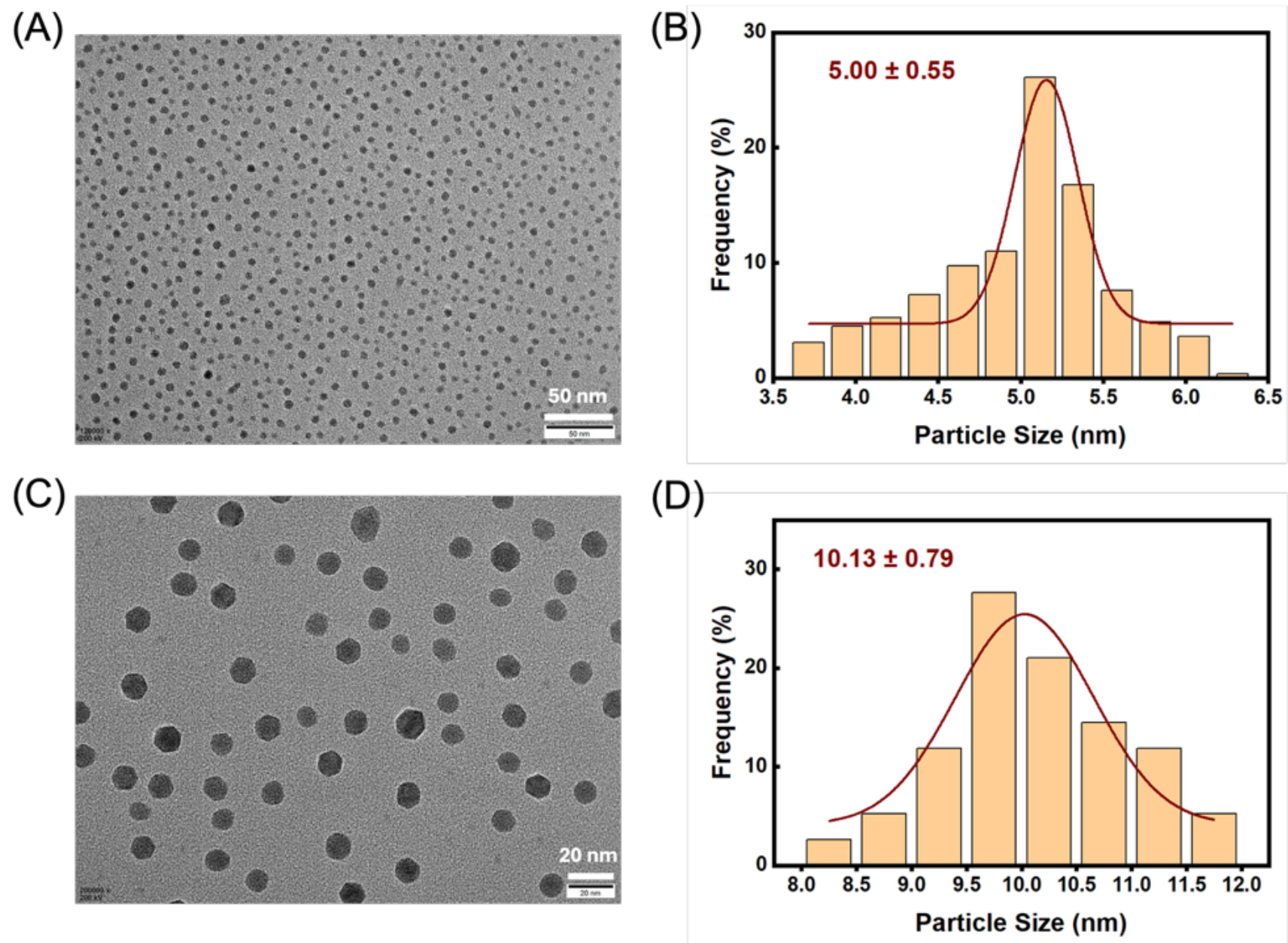

Fig. S10 TEM images of the recycled Pd-NP@OMC catalysts. (A) TEM image of recycled Pd-NP@PHEN-OMC, scale bar = 50 nm. (B) Size distribution of the recycled Pd-NP@PHEN-OMC. (C) TEM image of recycled Pd-NP@BPY-OMC, scale bar = 20 nm. (D) Size distribution of the recycled Pd-NP@BPY-OMC.

11. NMR Spectra of Cage Precursors and OMC Compounds

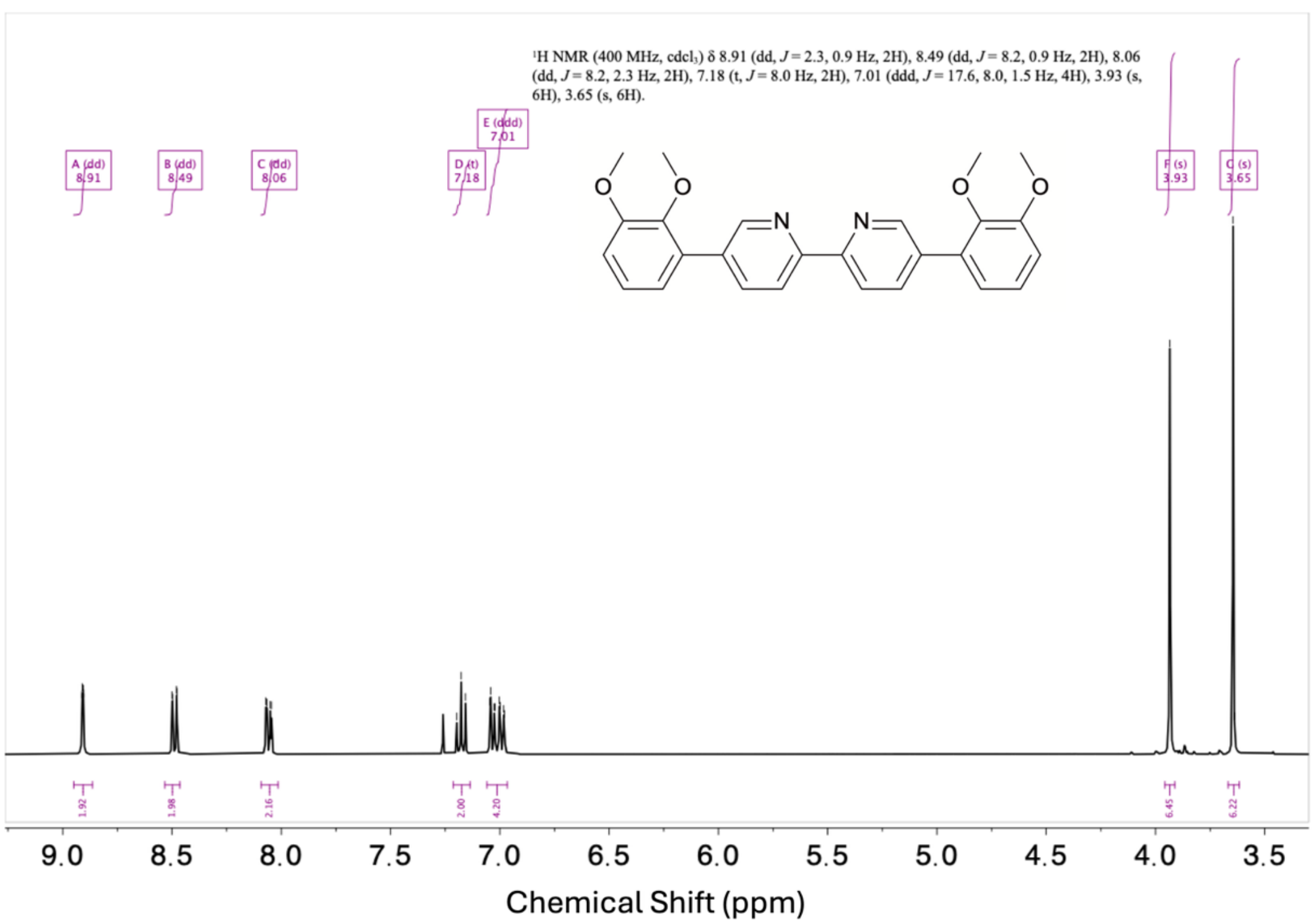

Fig. S11 ^{1}H NMR spectrum of 5,5'-bis(2,3-dimethoxyphenyl)-2,2'-bipyridine (BPY-4OMe) in deuterated chloroform.

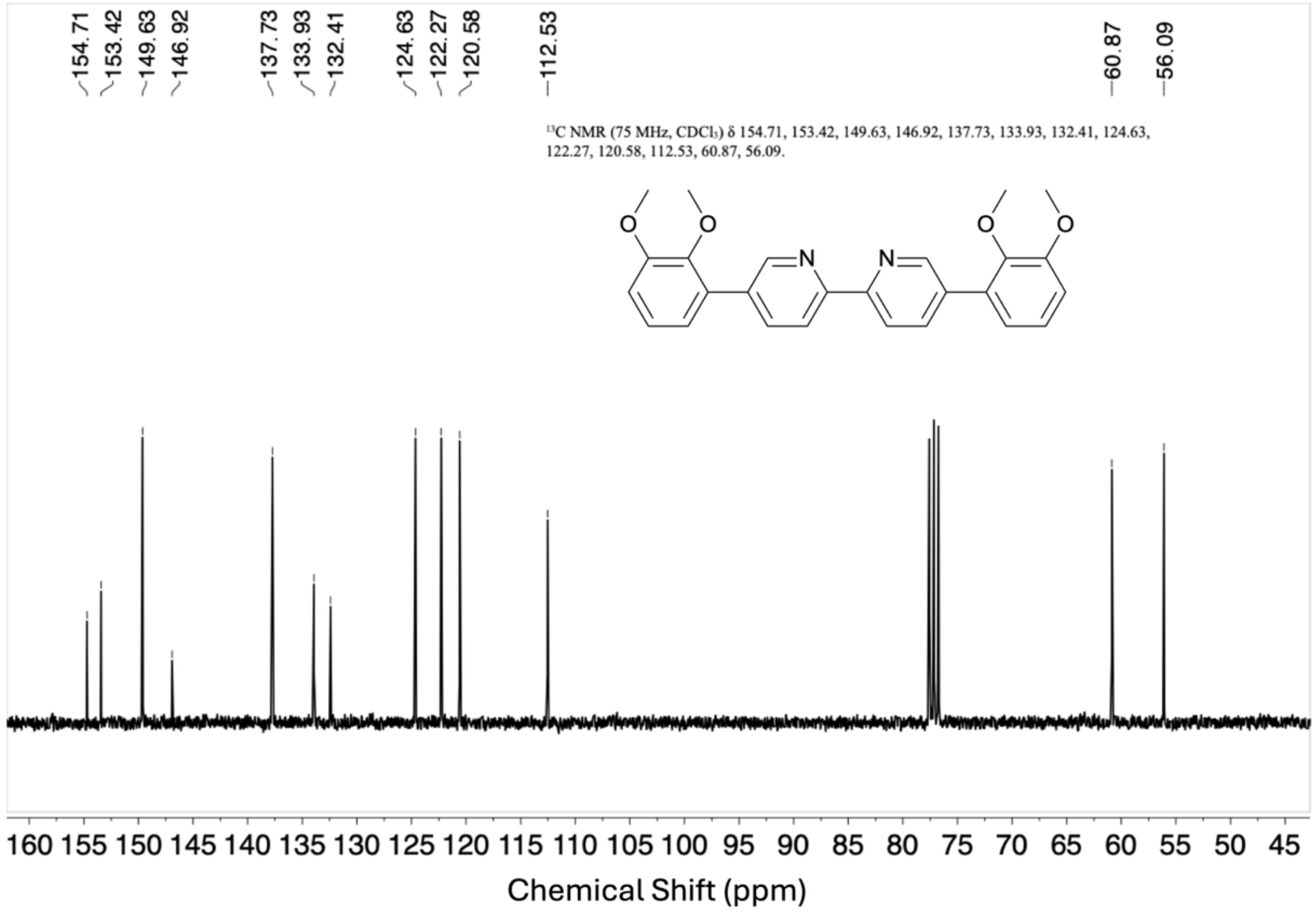

Fig. S12 ^{13}C NMR spectrum of BPY-4OMe in deuterated chloroform.

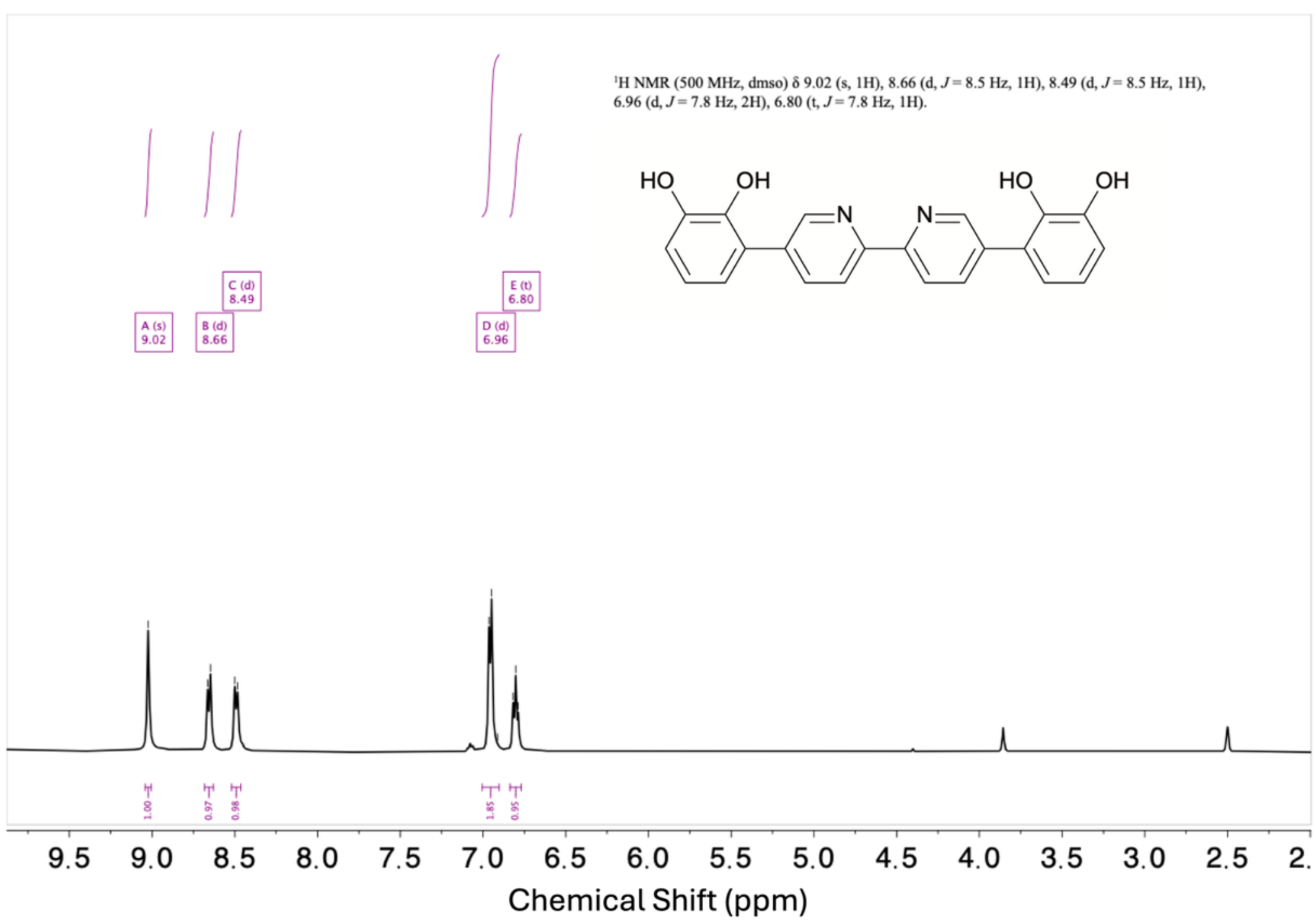

Fig. S13 ^{1}H NMR spectrum of 5,5'-bis(2,3-dihydroxyphenyl)-2,2'-bipyridine (BPY-4OH) in deuterated DMSO.

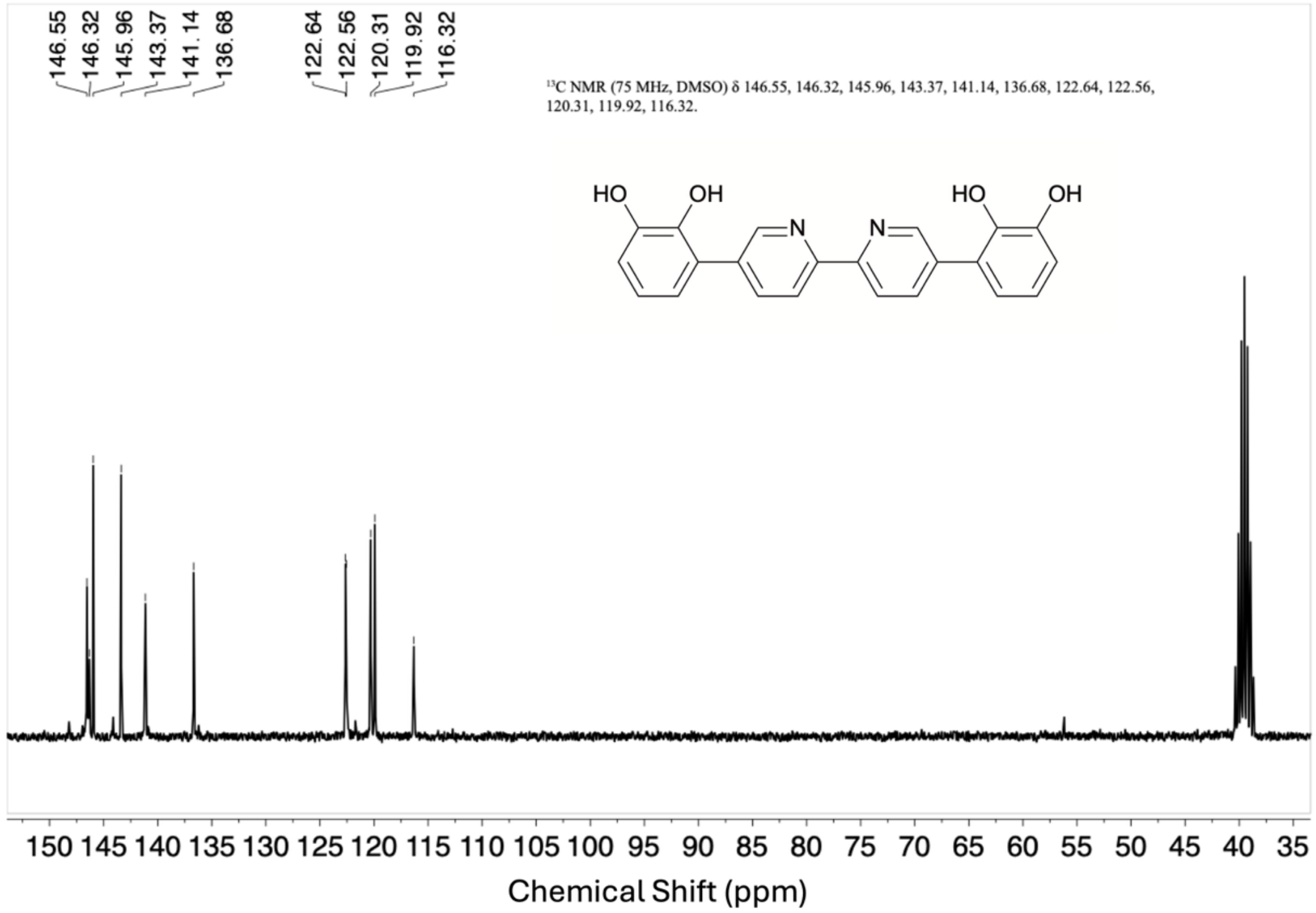

Fig. S14 ^{13}C NMR spectrum of BPY-4OH in deuterated DMSO.

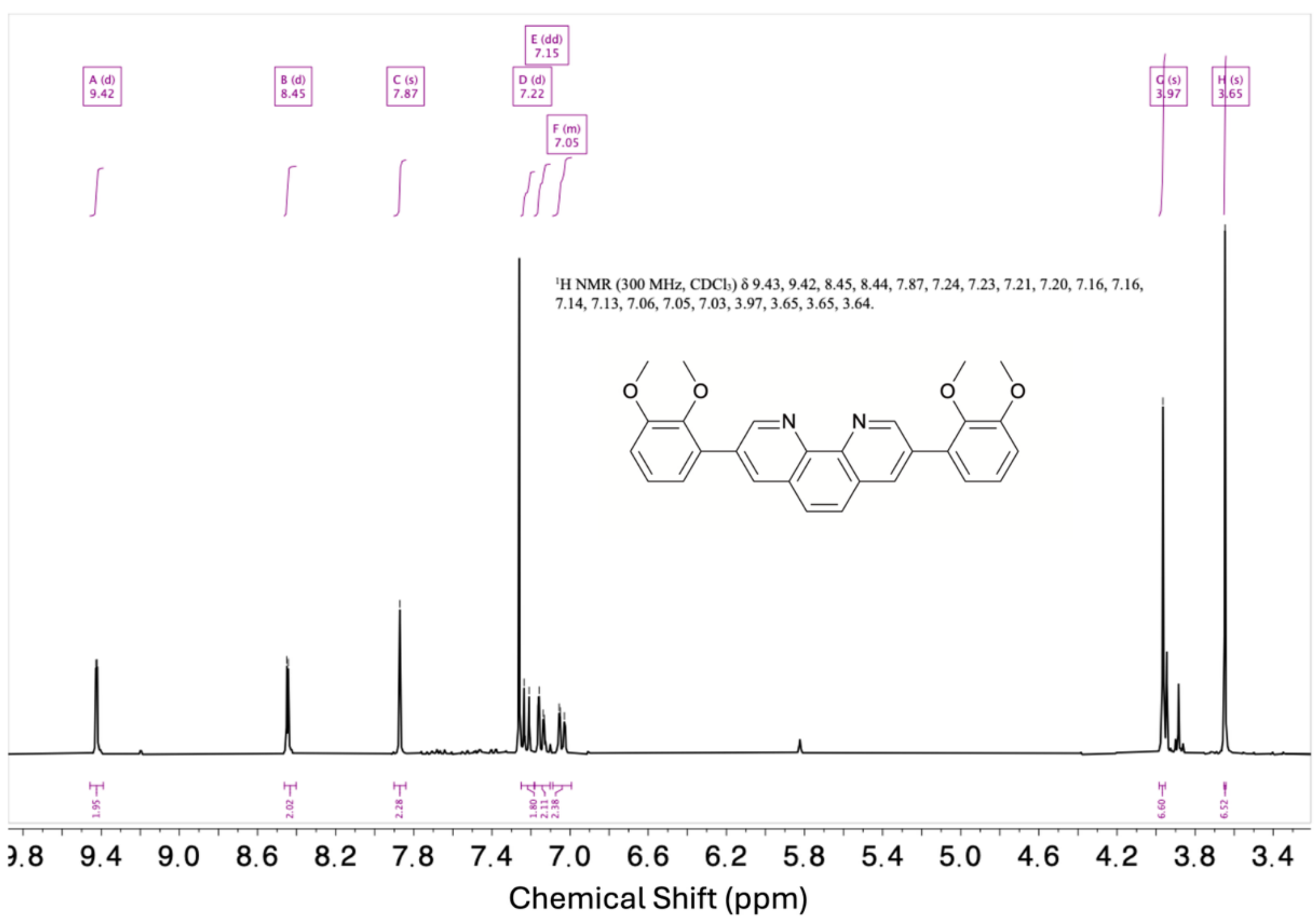

Fig. S15 ^{1}H NMR spectrum of 3,8-bis(2,3-dimethoxyphenyl)-1,10-phenanthroline (PHEN-4OMe) in deuterated chloroform.

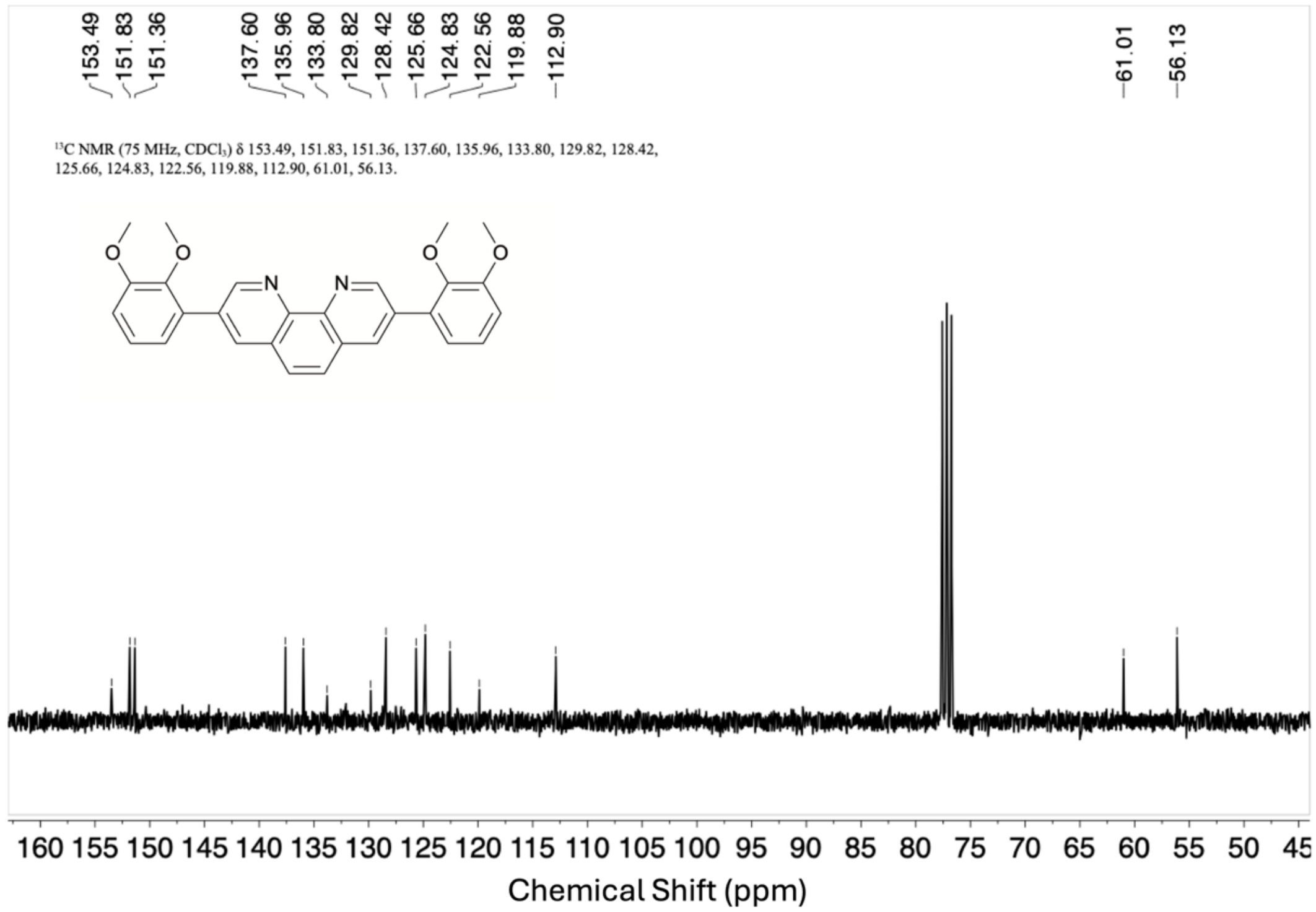

Fig. S16 ¹³C NMR spectrum of PHEN-4OMe in deuterated chloroform.

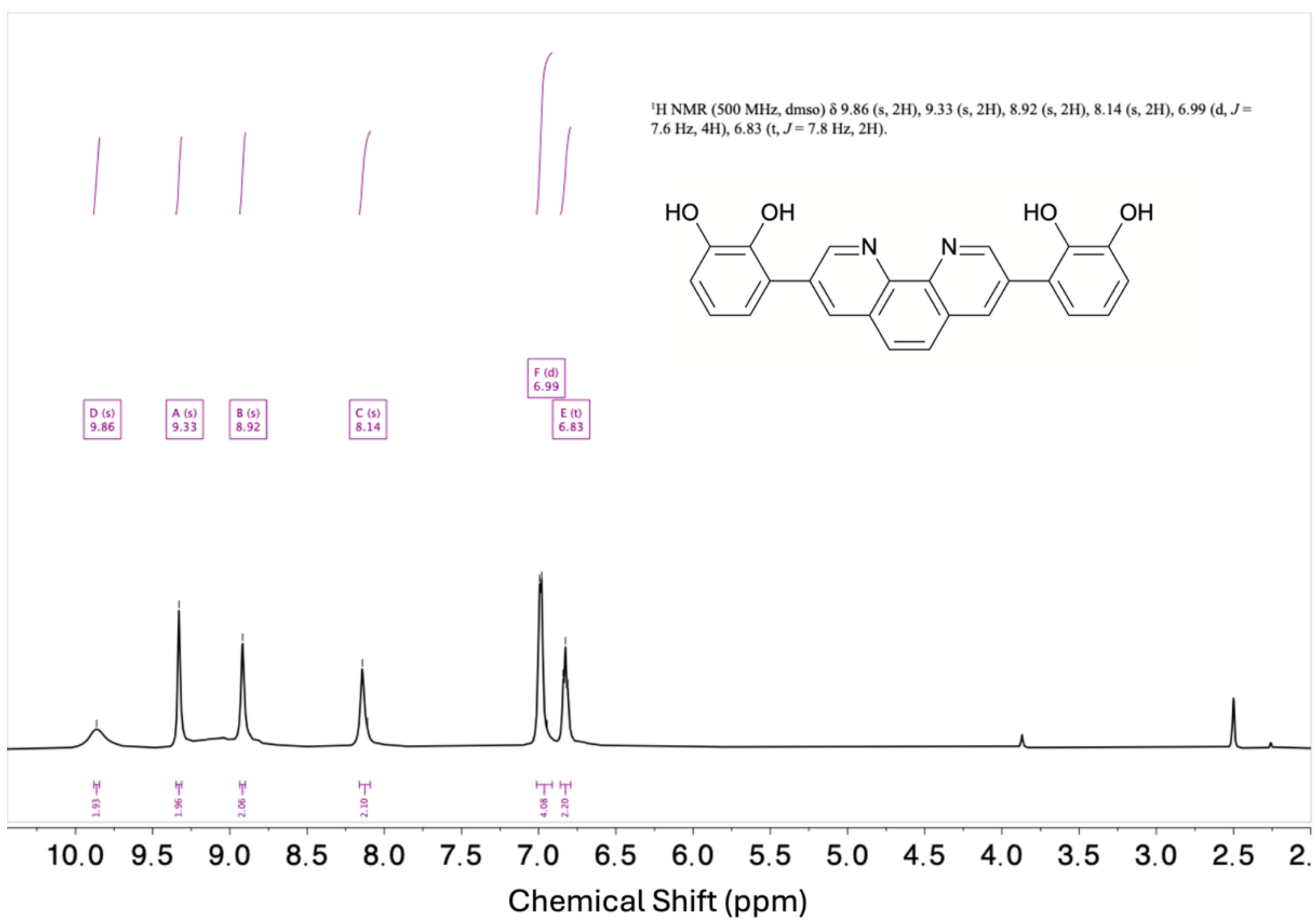

Fig. S17 ¹H NMR spectrum of 3,8-bis(2,3-dihydroxyphenyl)-1,10-phenanthroline (PHEN-4OH) in deuterated DMSO.

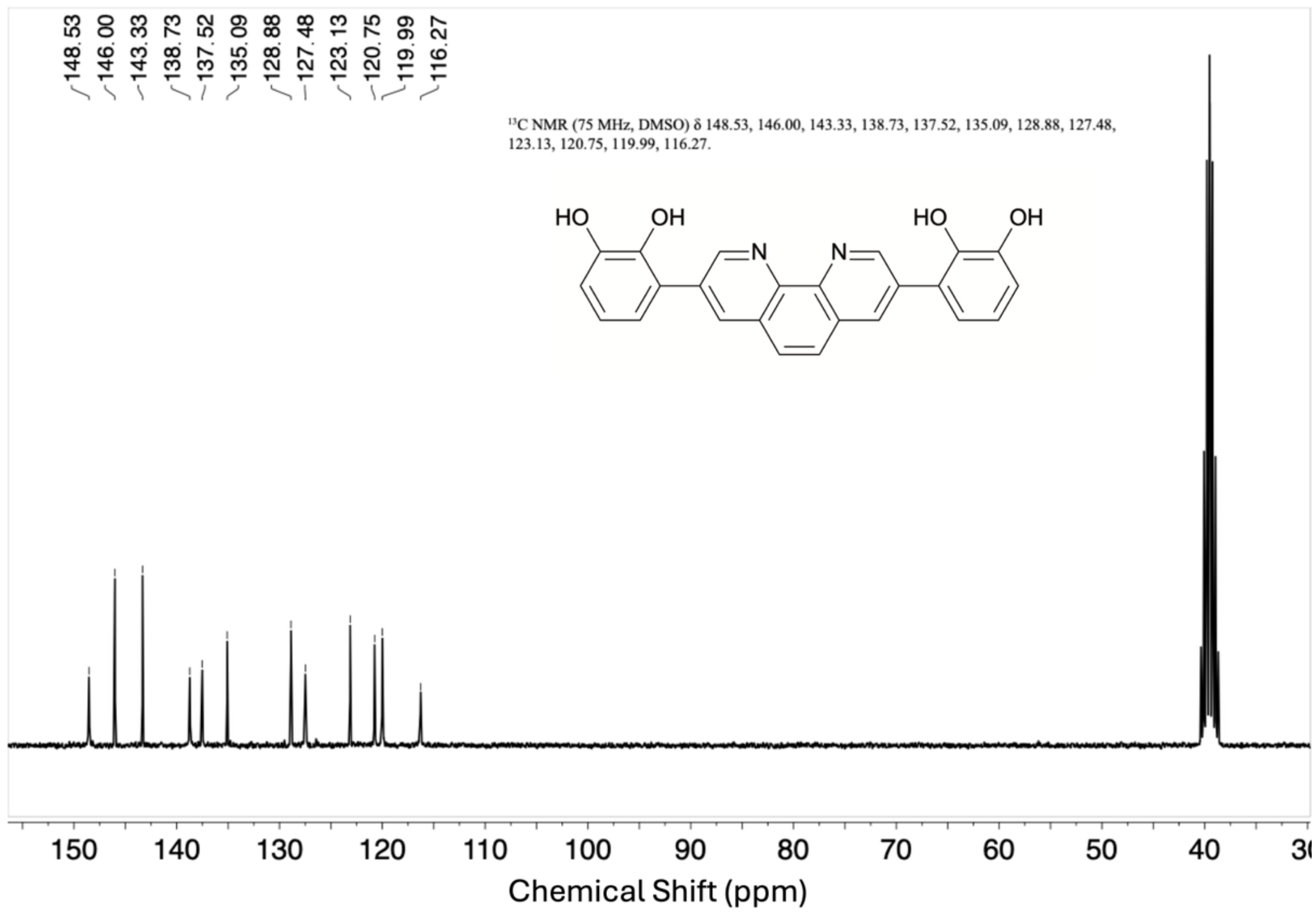

Fig. S18 ^{13}C NMR spectrum of PHEN-4OH in deuterated DMSO.

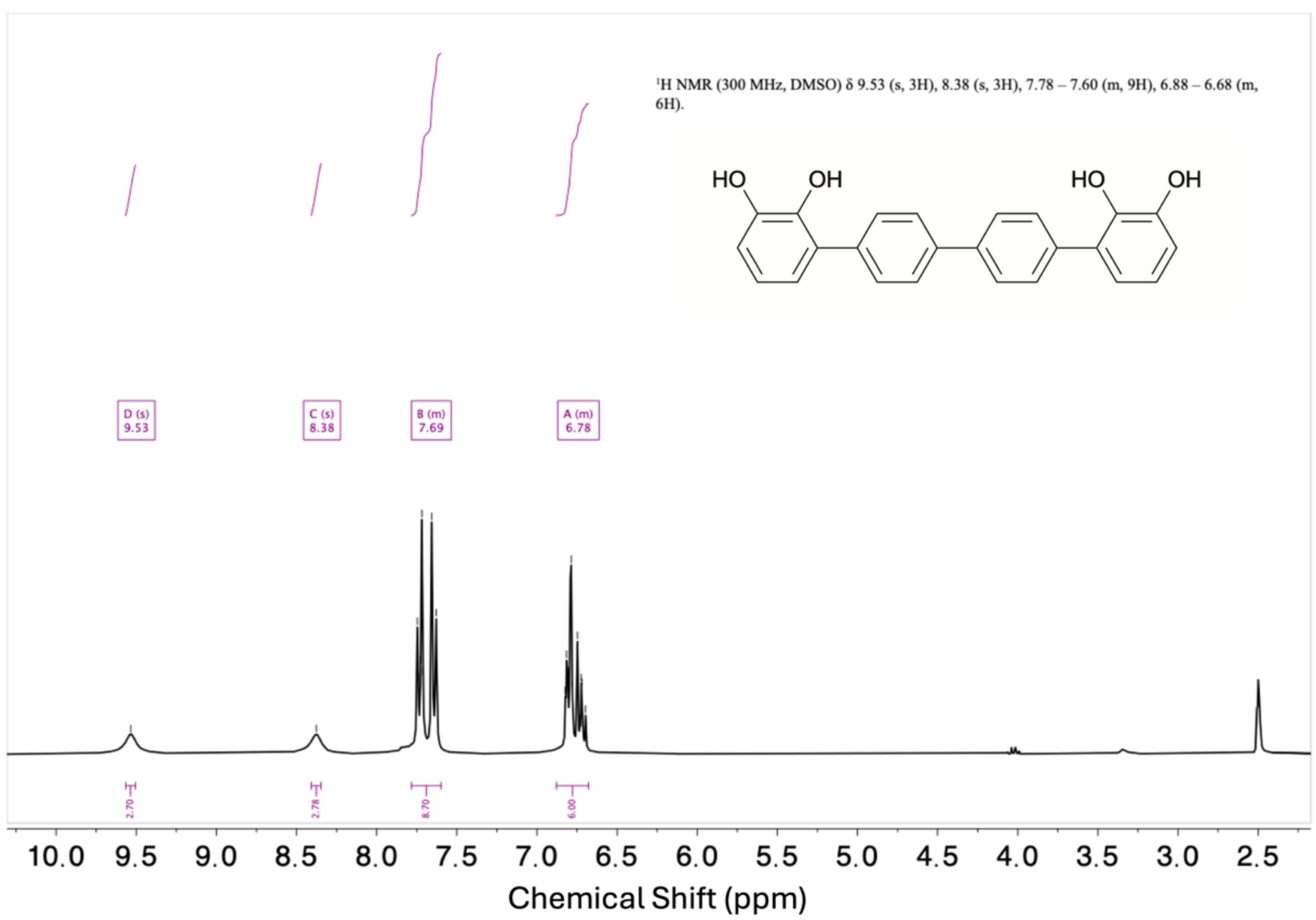

Fig. S19 ^{1}H NMR spectrum of 2,3,2''',3'''-tetrahydroxy-[1,1':4',1'':4'',1'''-quaterphenyl] (QP-4OH) in deuterated DMSO.

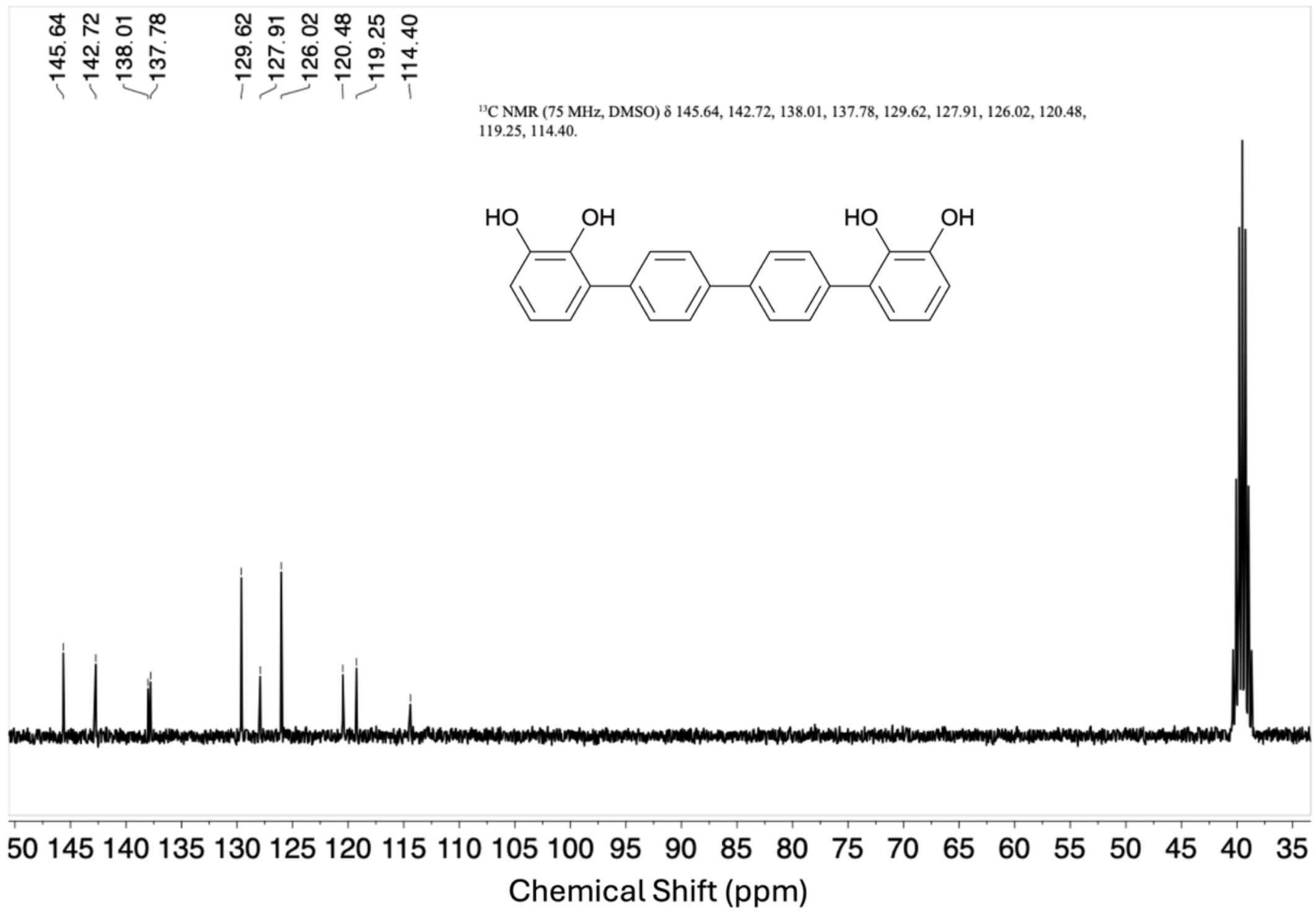

Fig. S20 ^{13}C NMR spectrum of QP-4OH in deuterated DMSO.

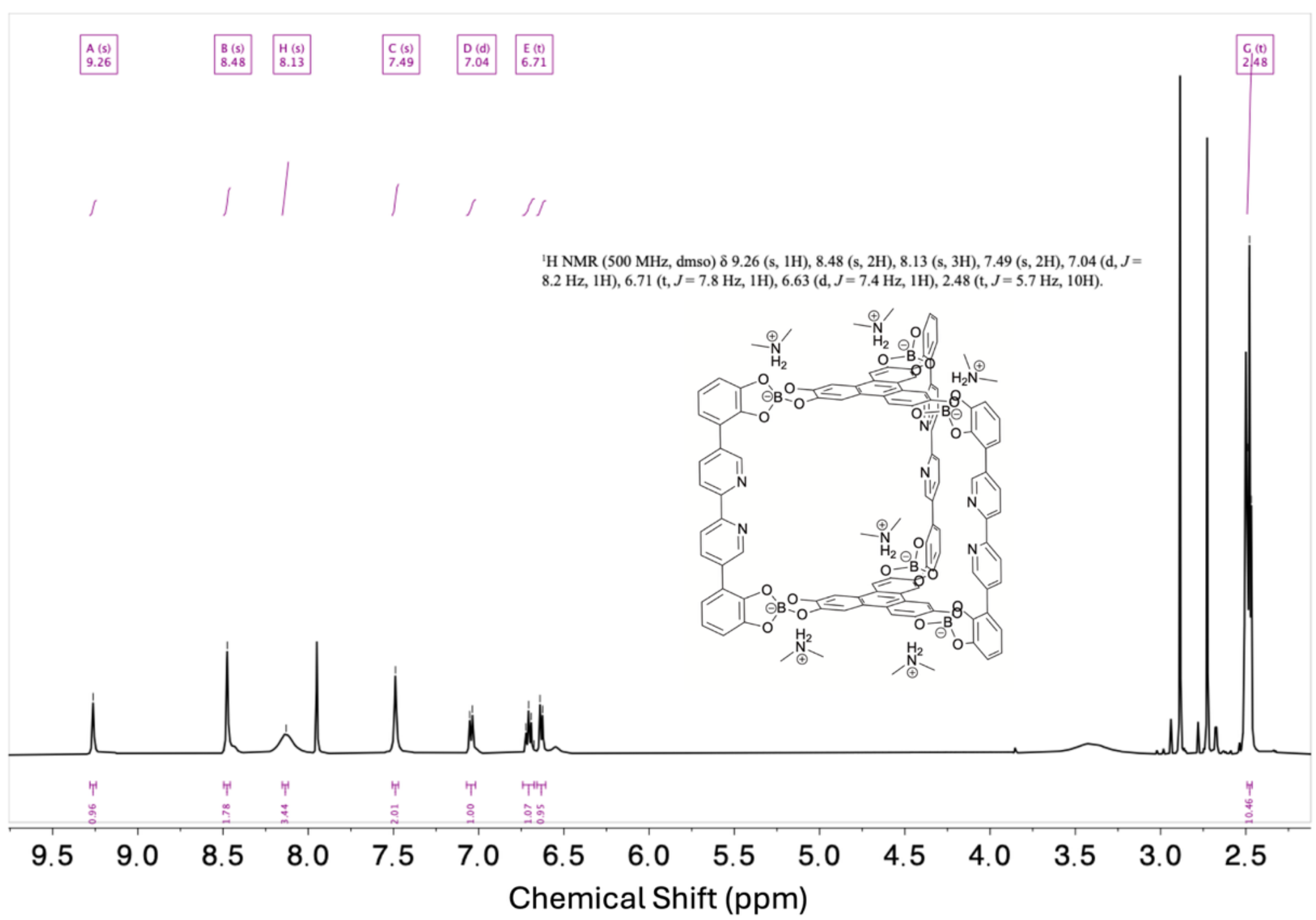

Fig. S21 ^{1}H NMR spectrum of BPY-OMC in deuterated DMSO.

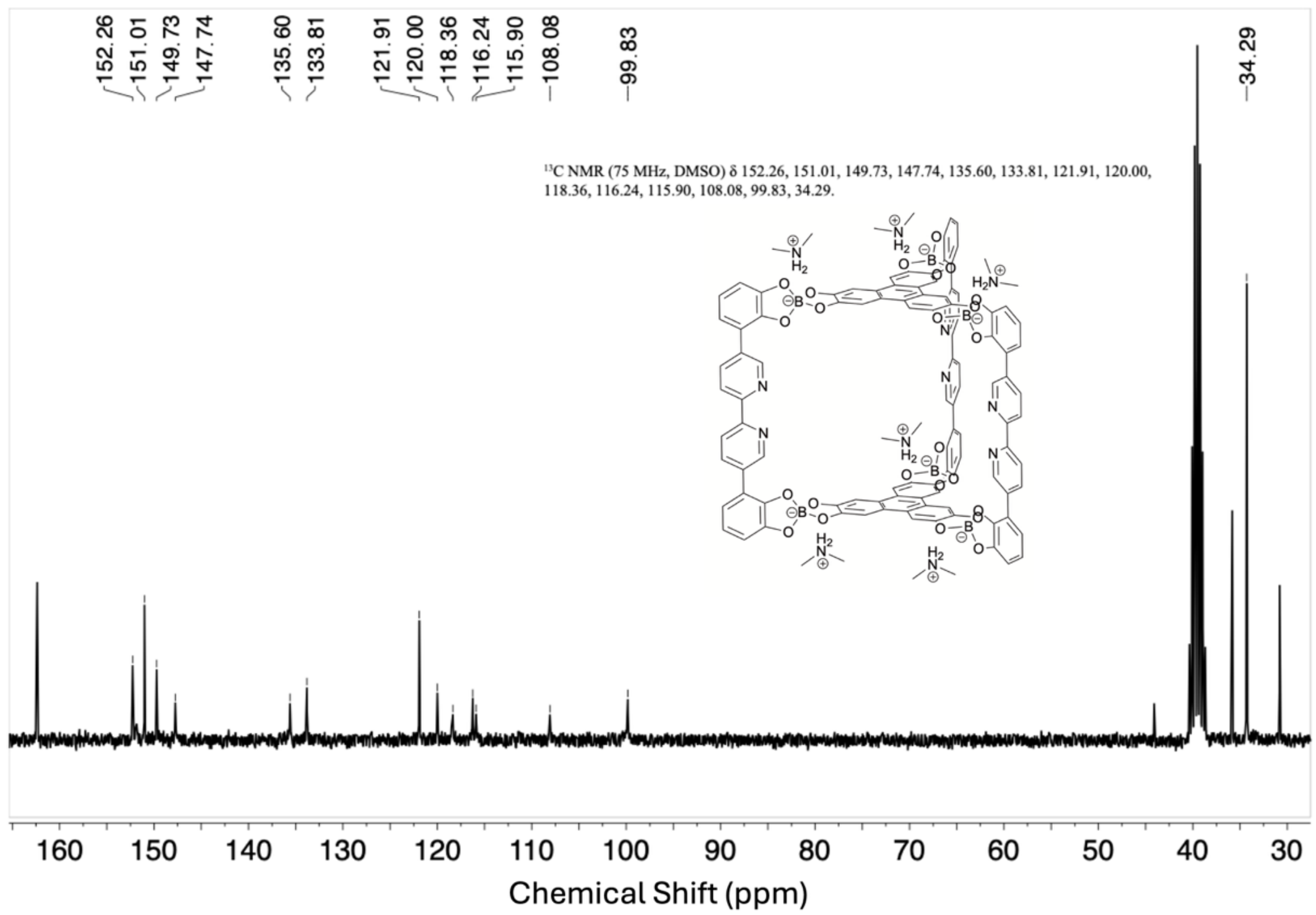

Fig. S22 13C NMR spectrum of BPY-OMC in deuterated DMSO.

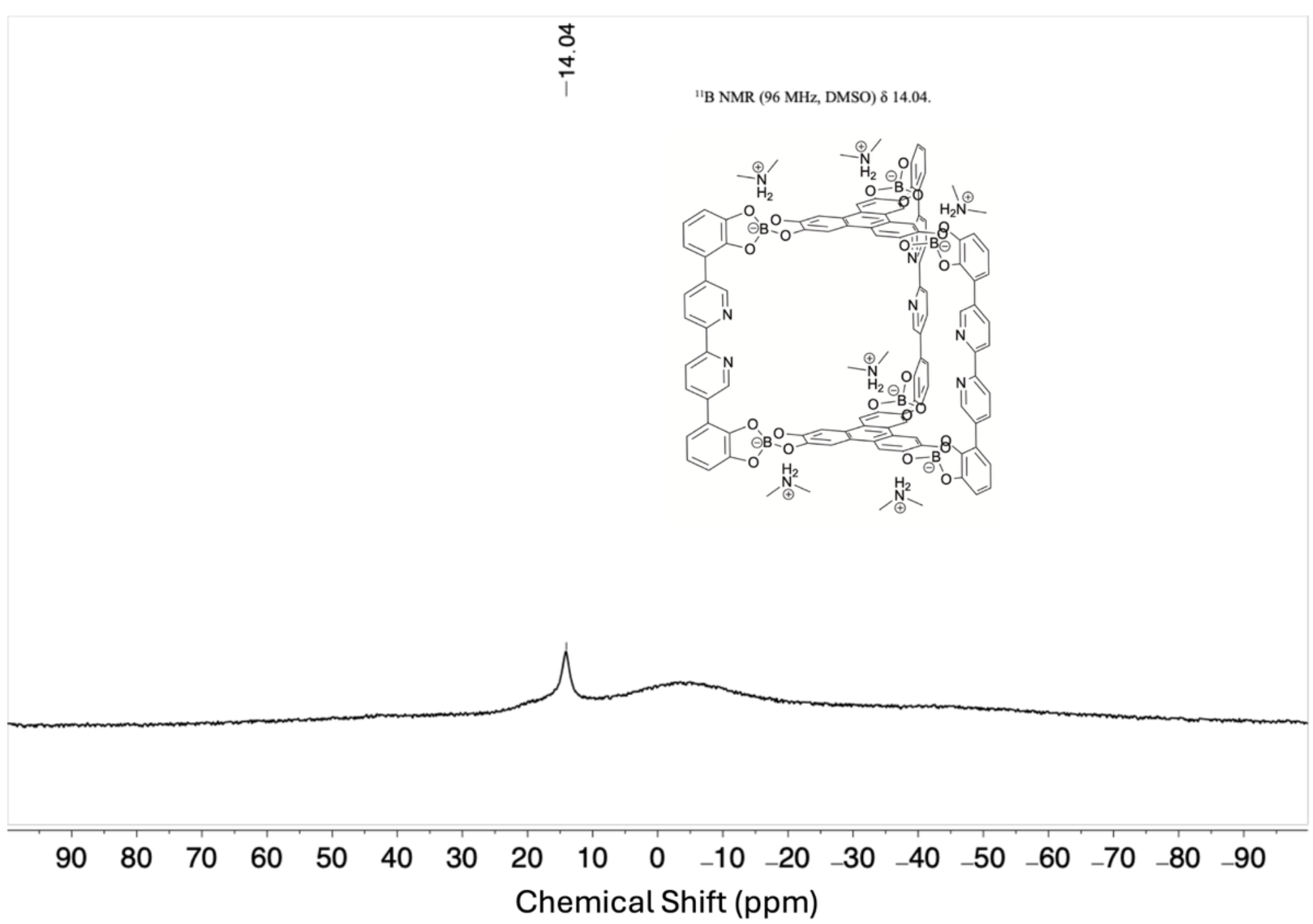

Fig. S23 11B NMR spectrum of BPY-OMC in deuterated DMSO.

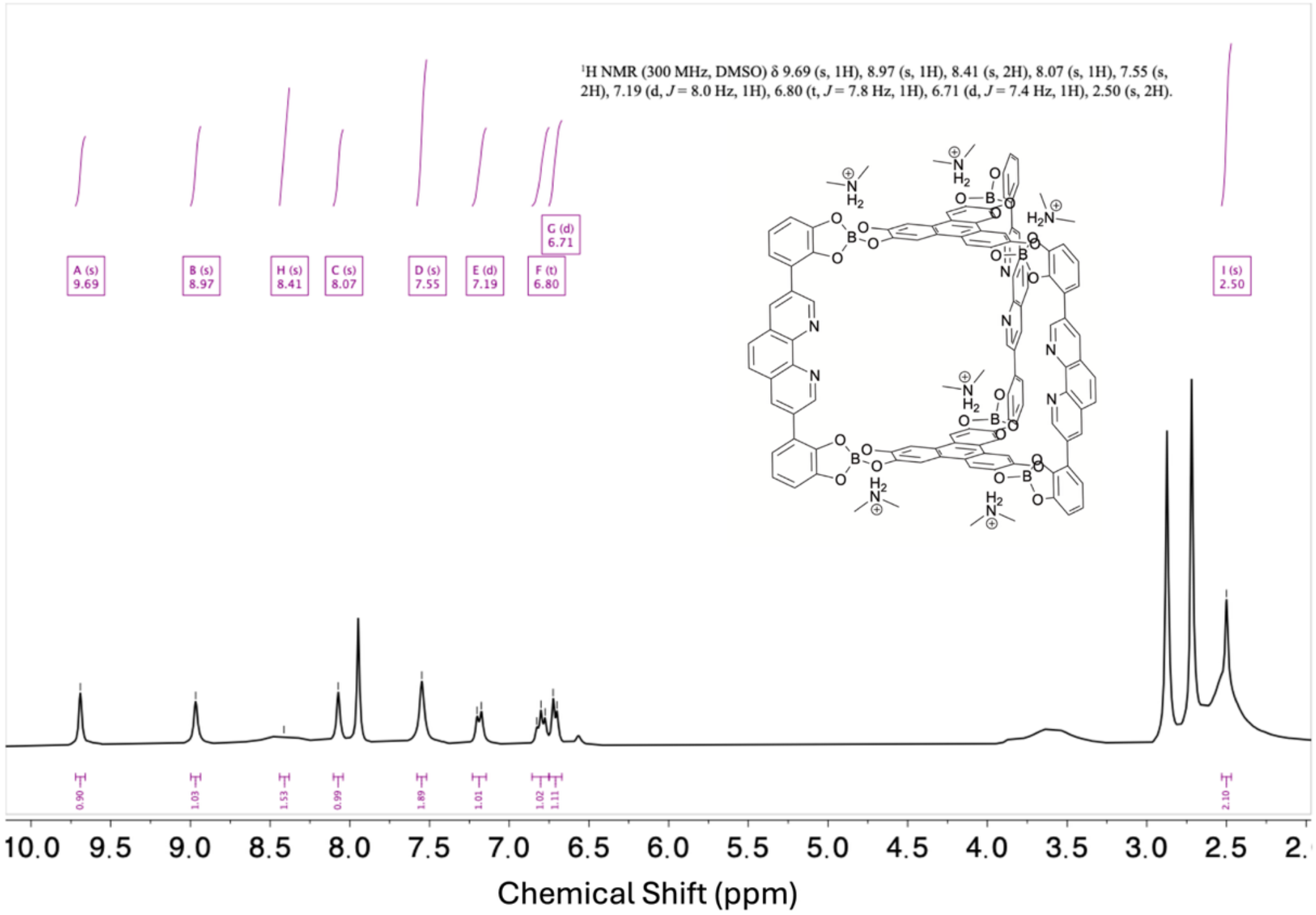

Fig. S24 ^{1}H NMR spectrum of PHEN-OMC in deuterated DMSO.

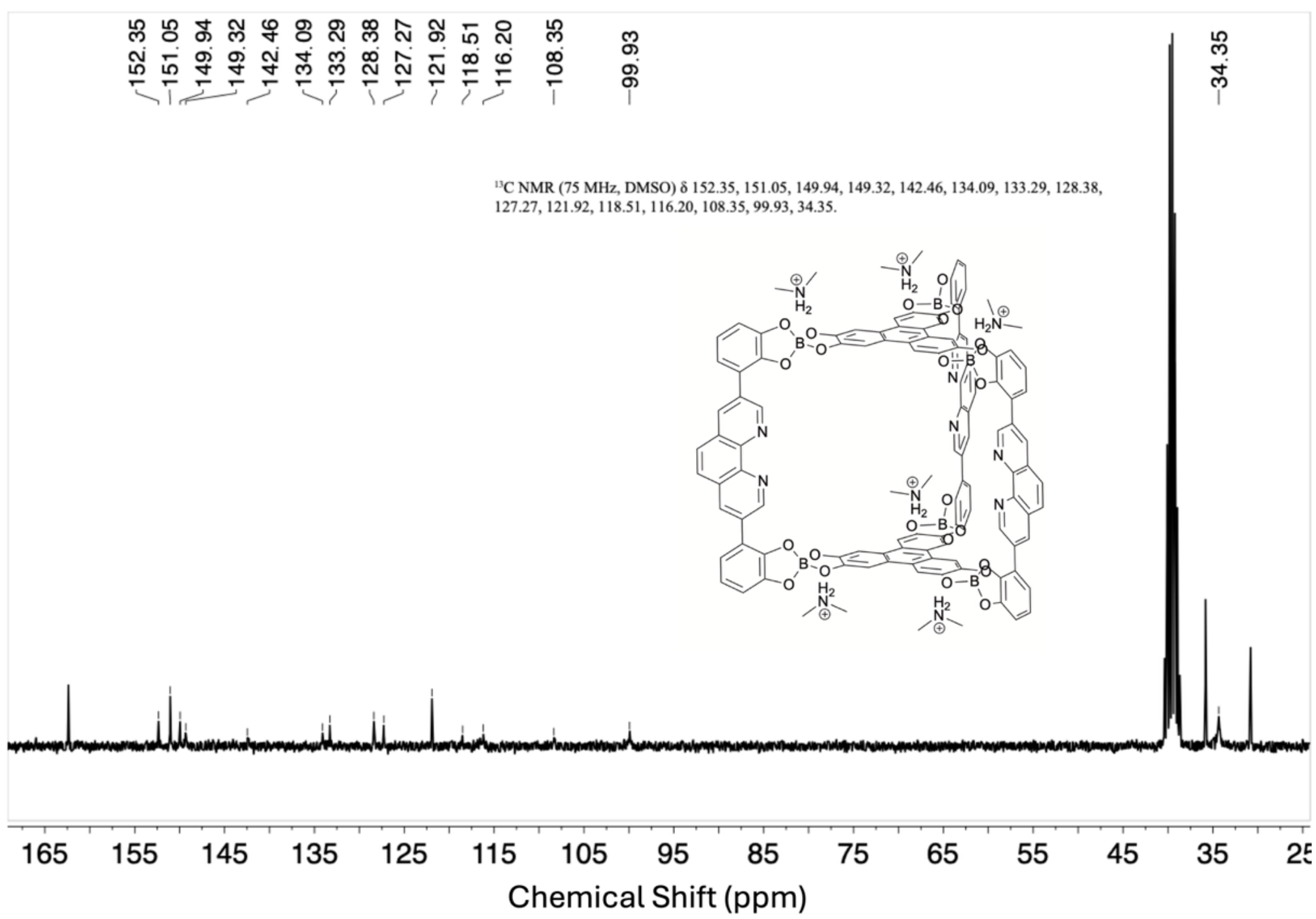

Fig. S25 ^{13}C NMR spectrum of PHEN-OMC in deuterated DMSO.

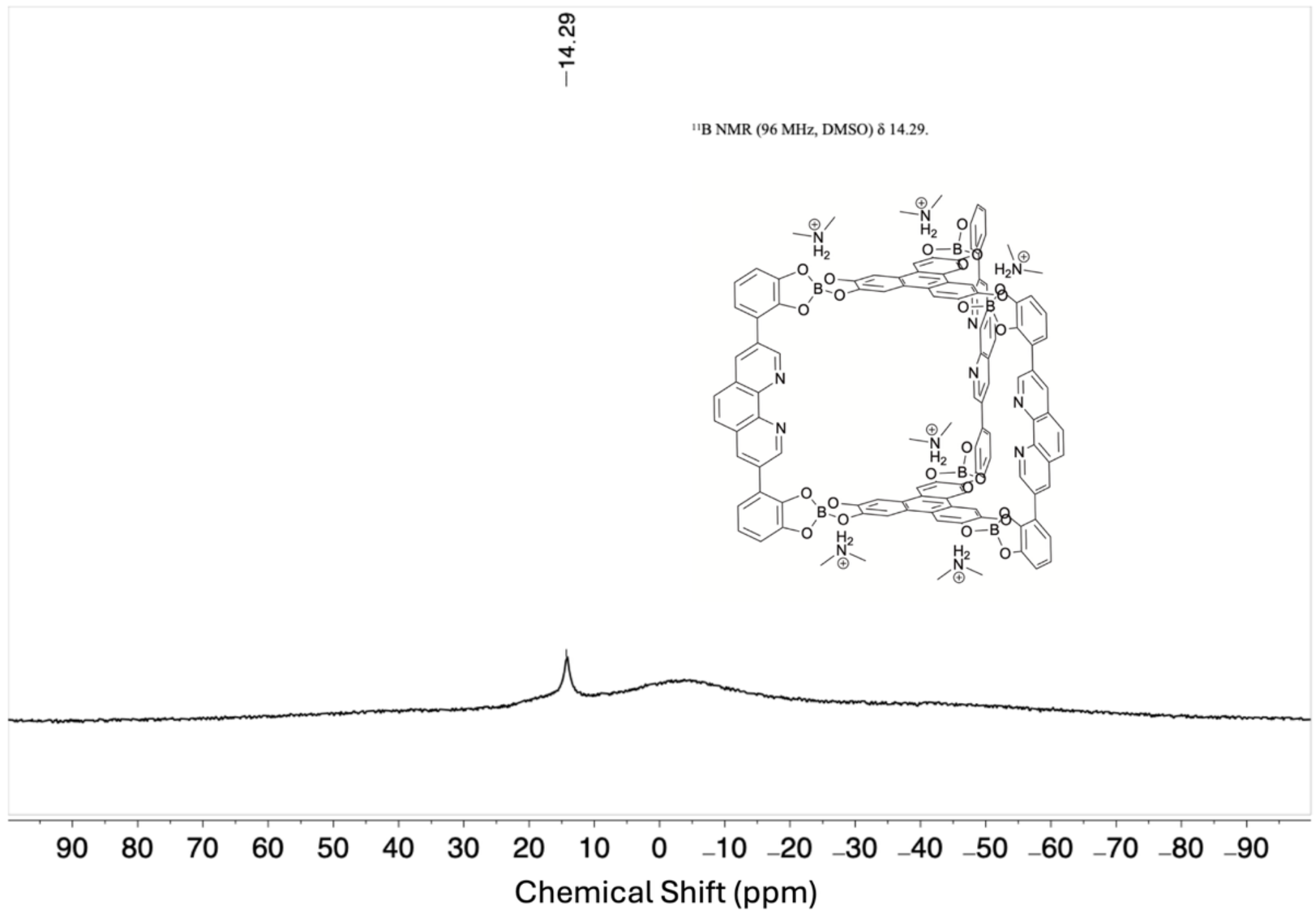

Fig. S26 ^{11}B NMR spectrum of PHEN-OMC in deuterated DMSO.

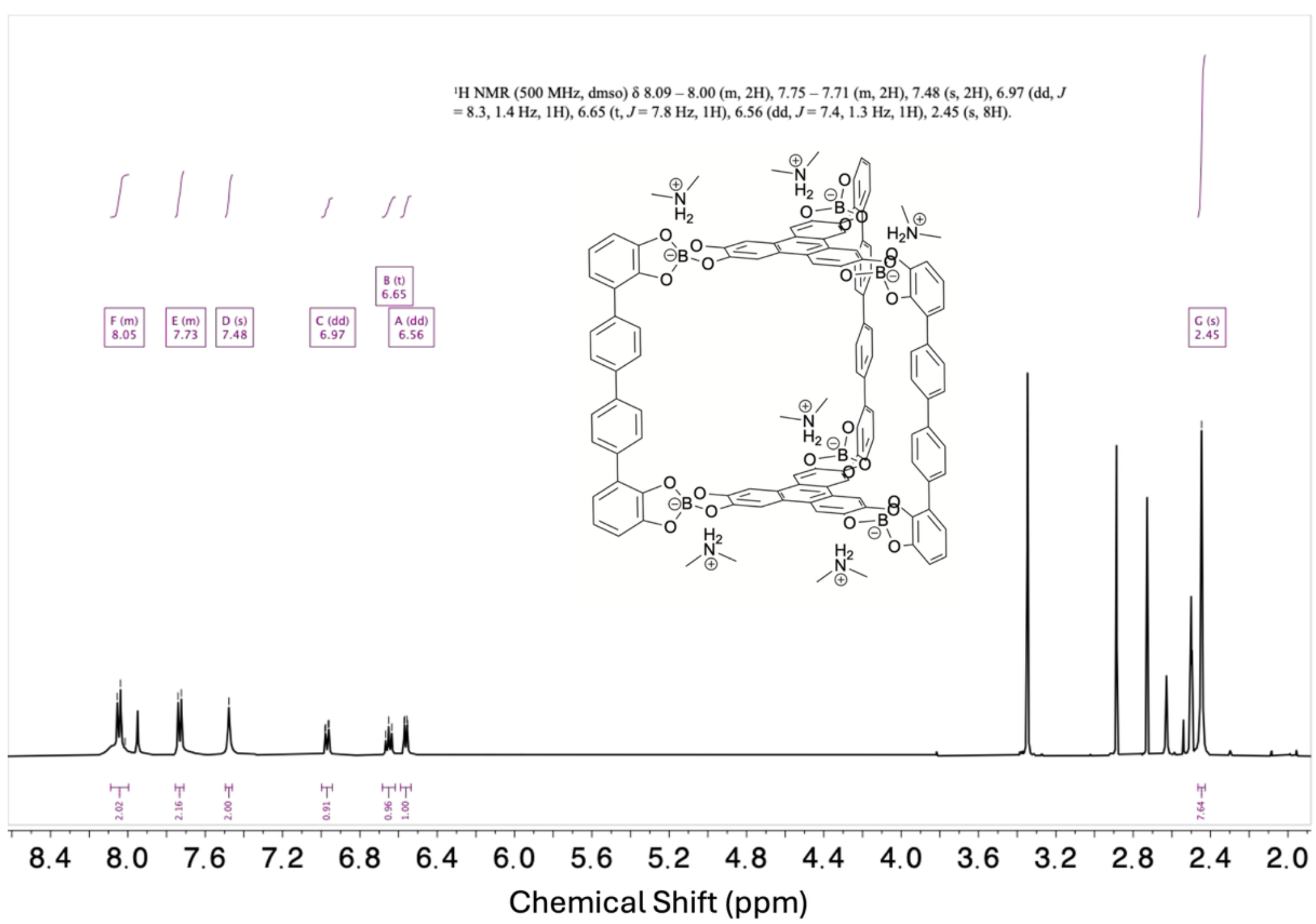

Fig. S27 ^{1}H NMR spectrum of QP-OMC in deuterated DMSO.

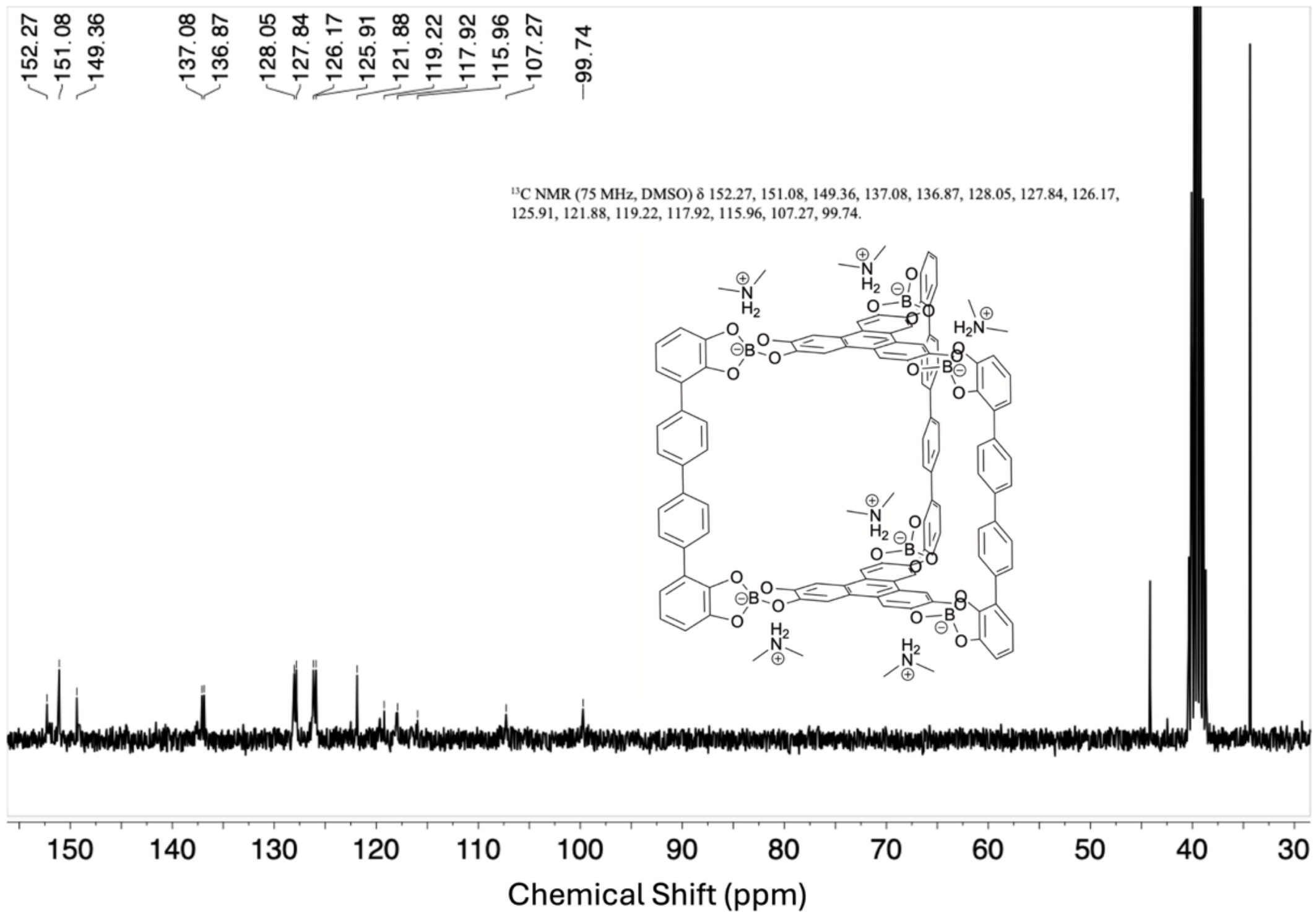

Fig. S28 ^{13}C NMR spectrum of QP-OMC in deuterated DMSO.

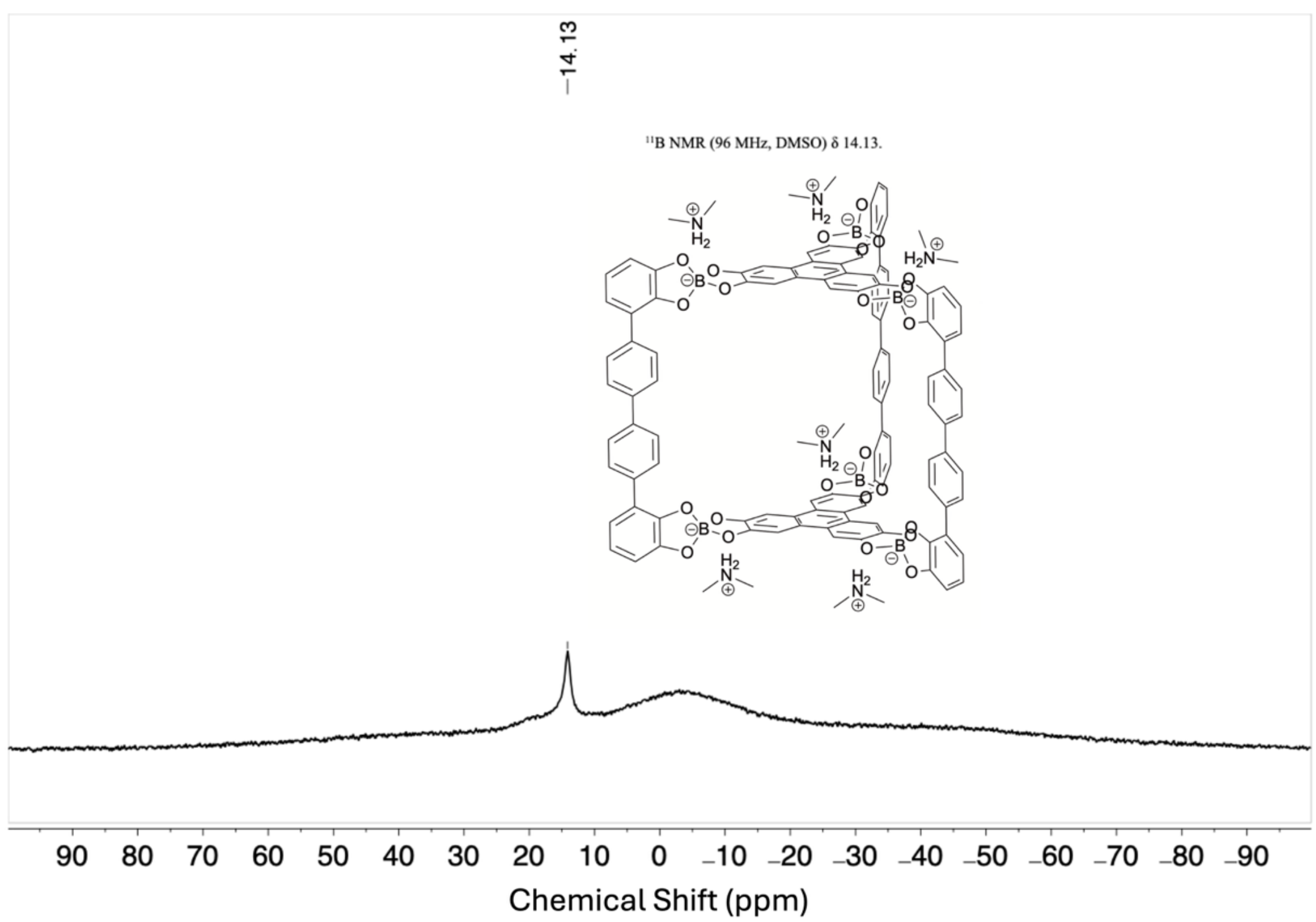

Fig. S29 ^{11}B NMR spectrum of QP-OMC in deuterated DMSO.

12. NMR Spectra of Extracted Catalysis Products

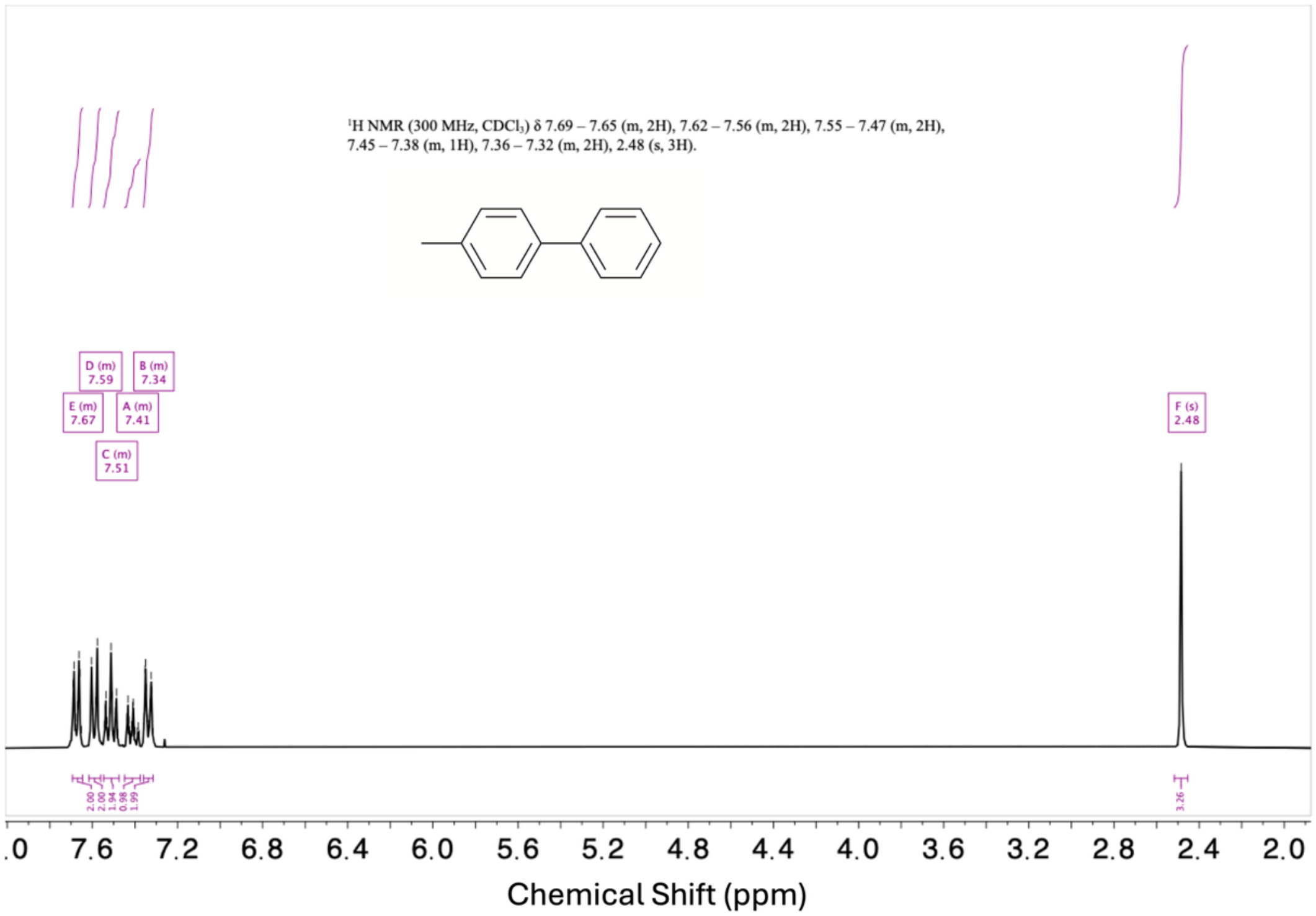

Fig. S30 ^{1}H NMR spectrum of 4-methyl-1,1'-biphenyl in deuterated chloroform.

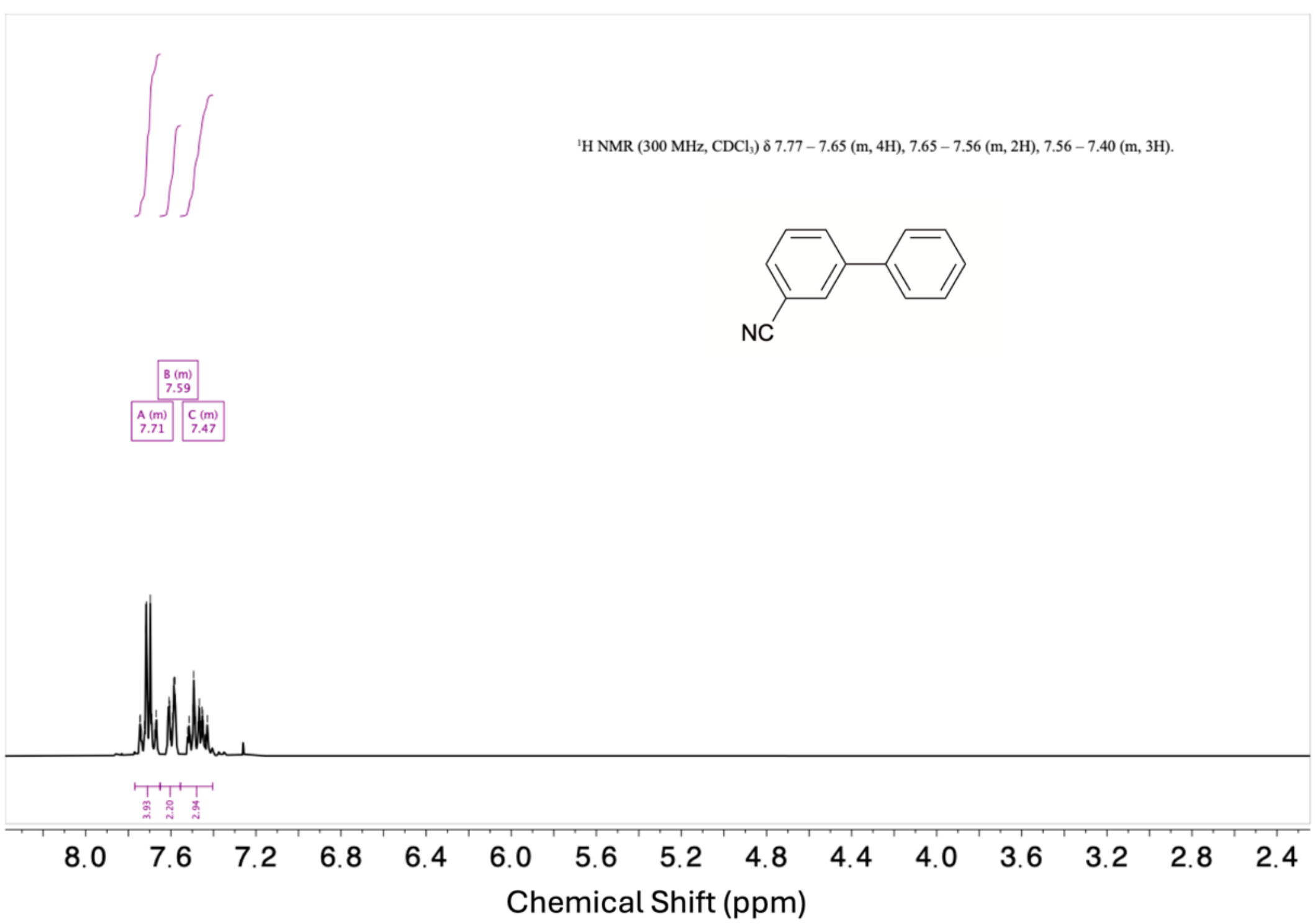

Fig. S31 ^{1}H NMR spectrum of 3-cyano-1,1'-biphenyl in deuterated chloroform.

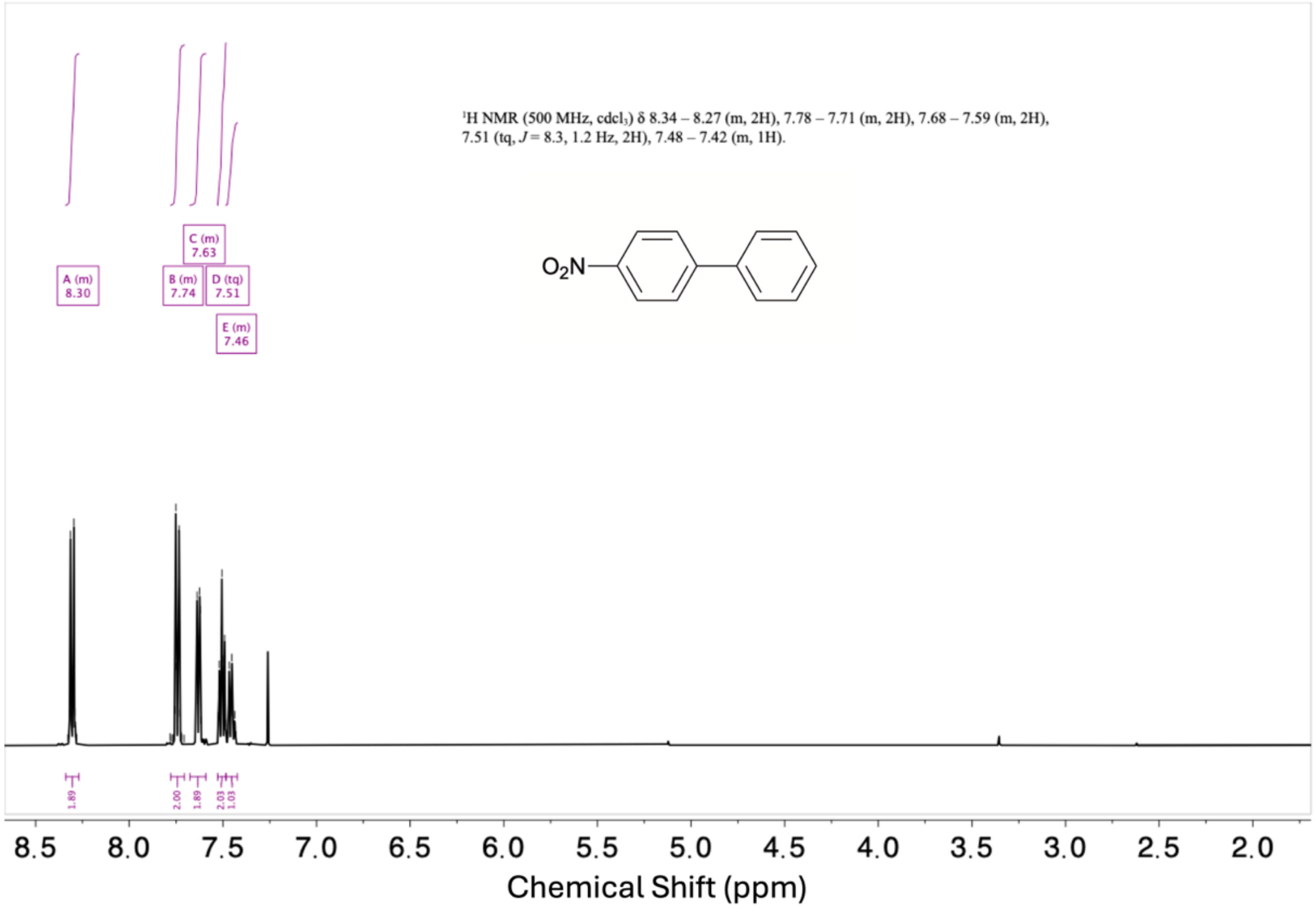

Fig. S32 ^{1}H NMR spectrum of 4-nitro-1,1′-biphenyl in deuterated chloroform.

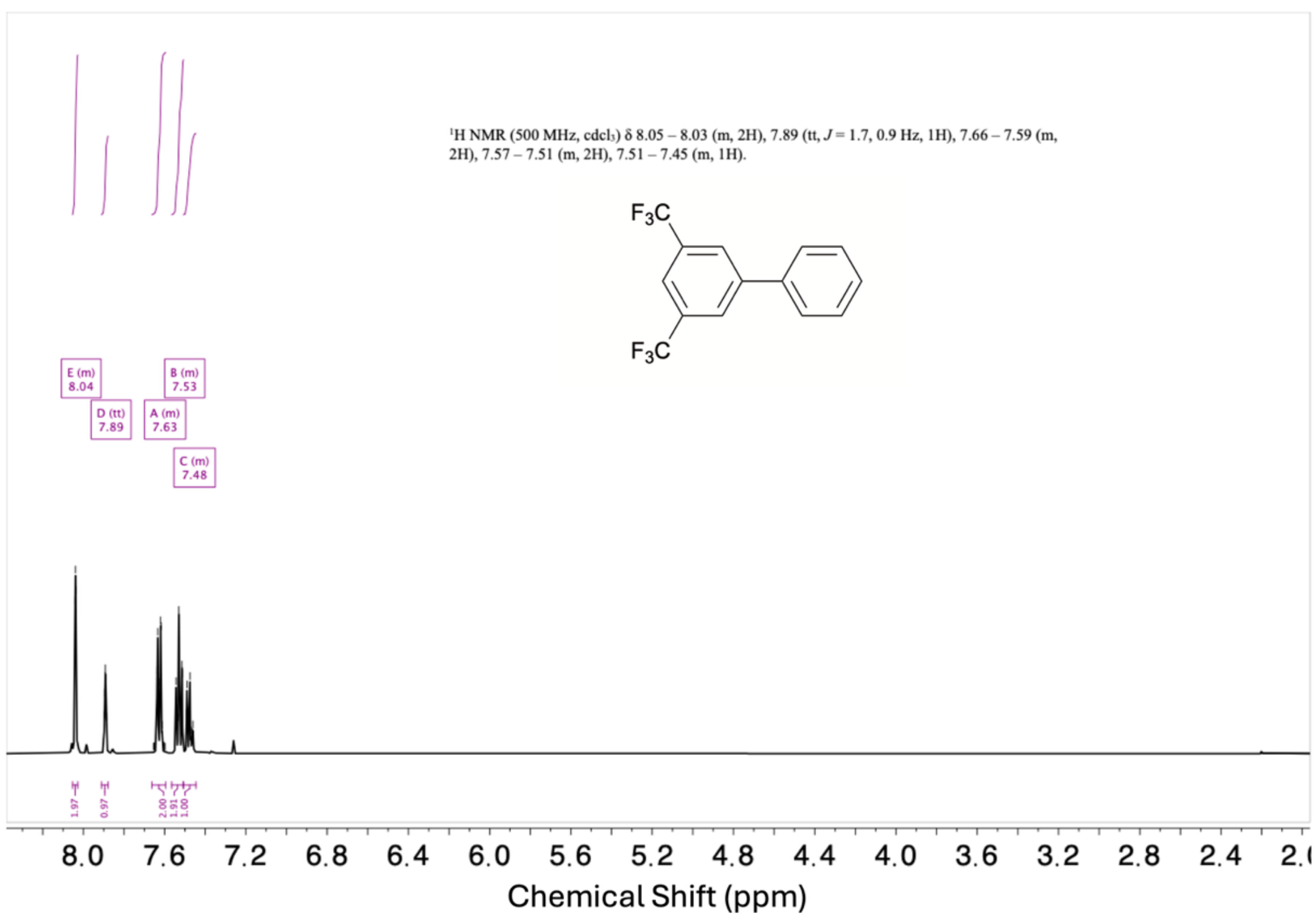

Fig. S33 ^{1}H NMR spectrum of 2,3-trifluoromethyl-1,1′-biphenyl in deuterated chloroform.

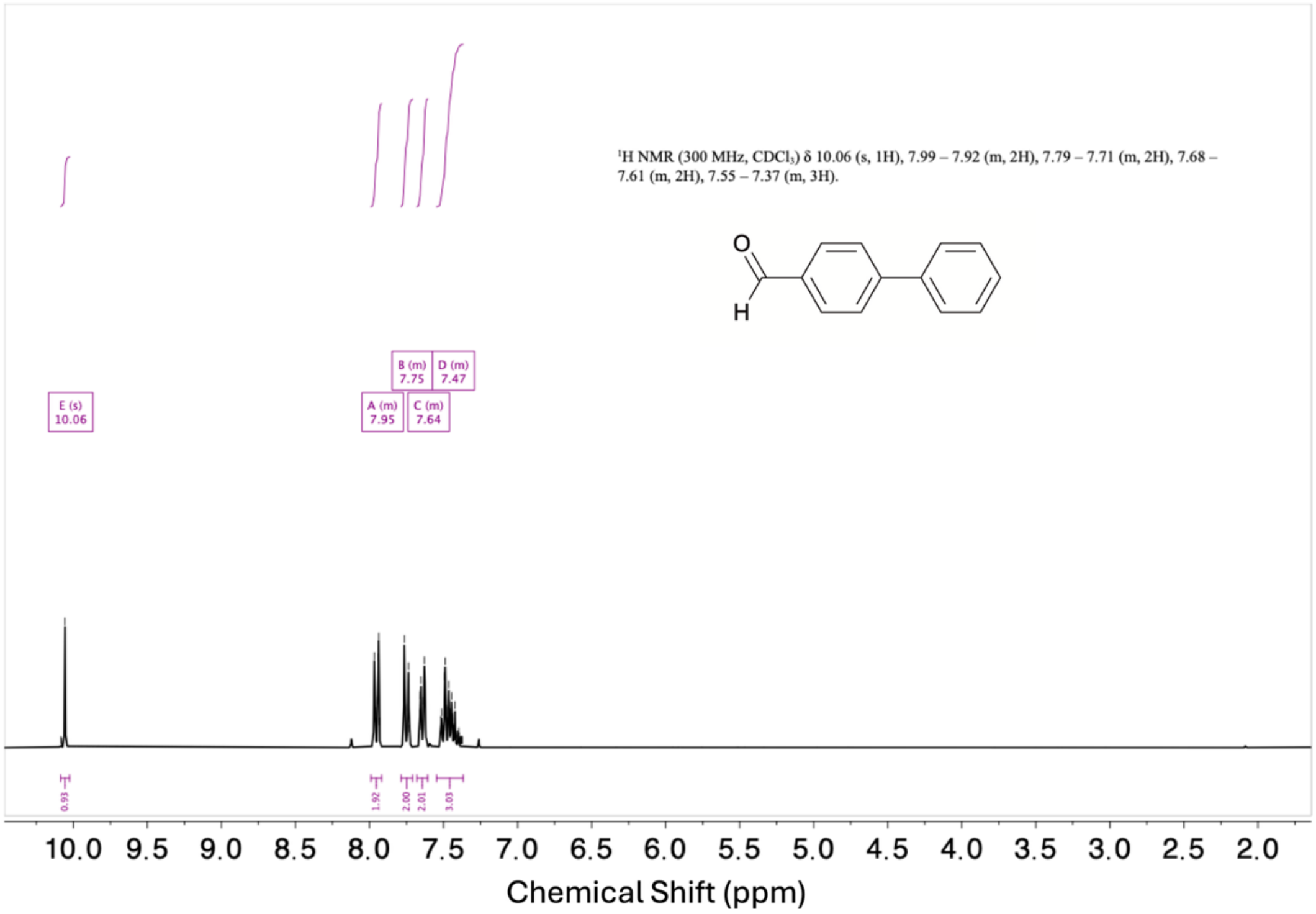

Fig. S34 ^{1}H NMR spectrum of 4-formyl-1,1′-biphenyl in deuterated chloroform.

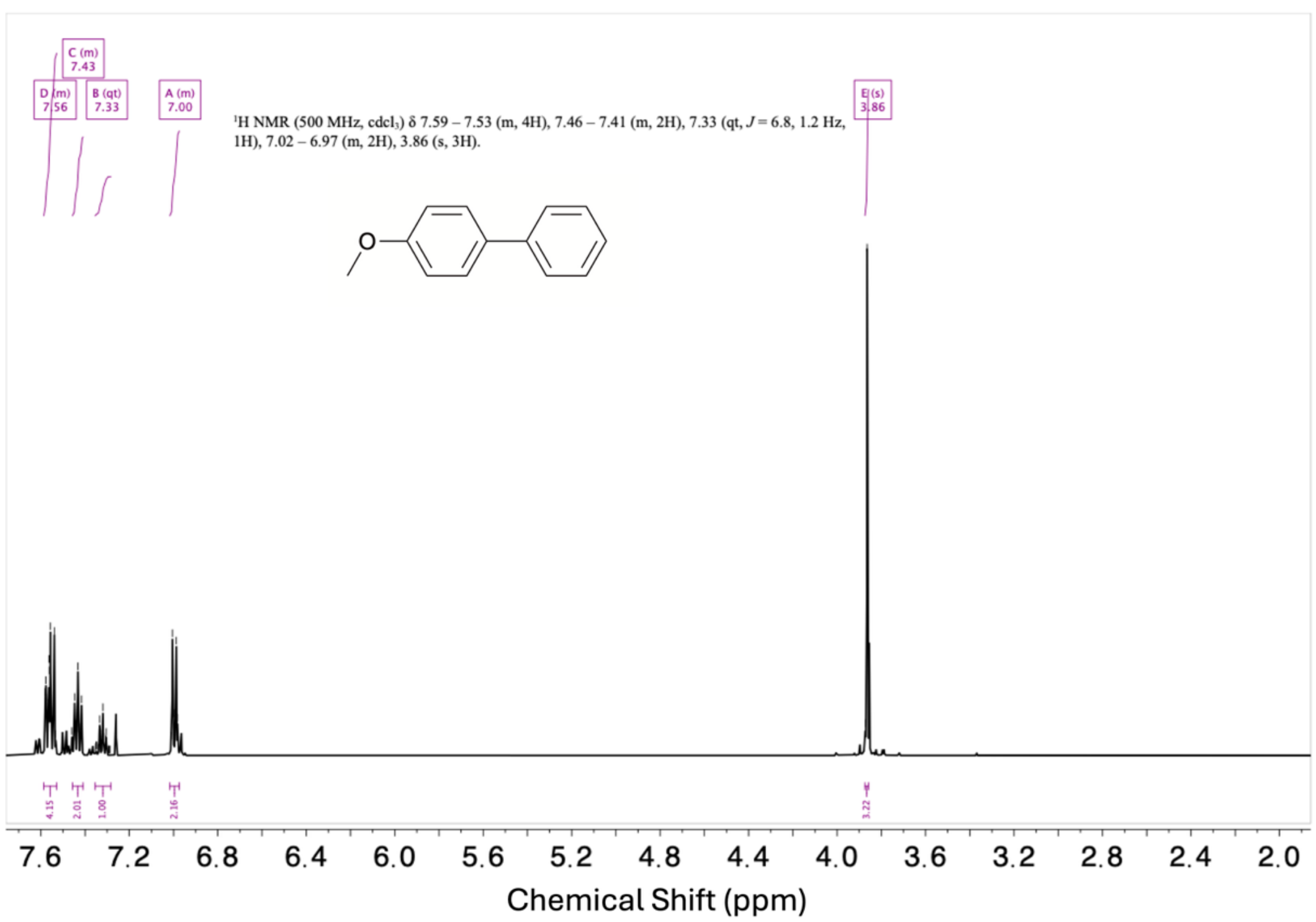

Fig. S35 ^{1}H NMR spectrum of 4-methoxy-1,1′-biphenyl in deuterated chloroform.

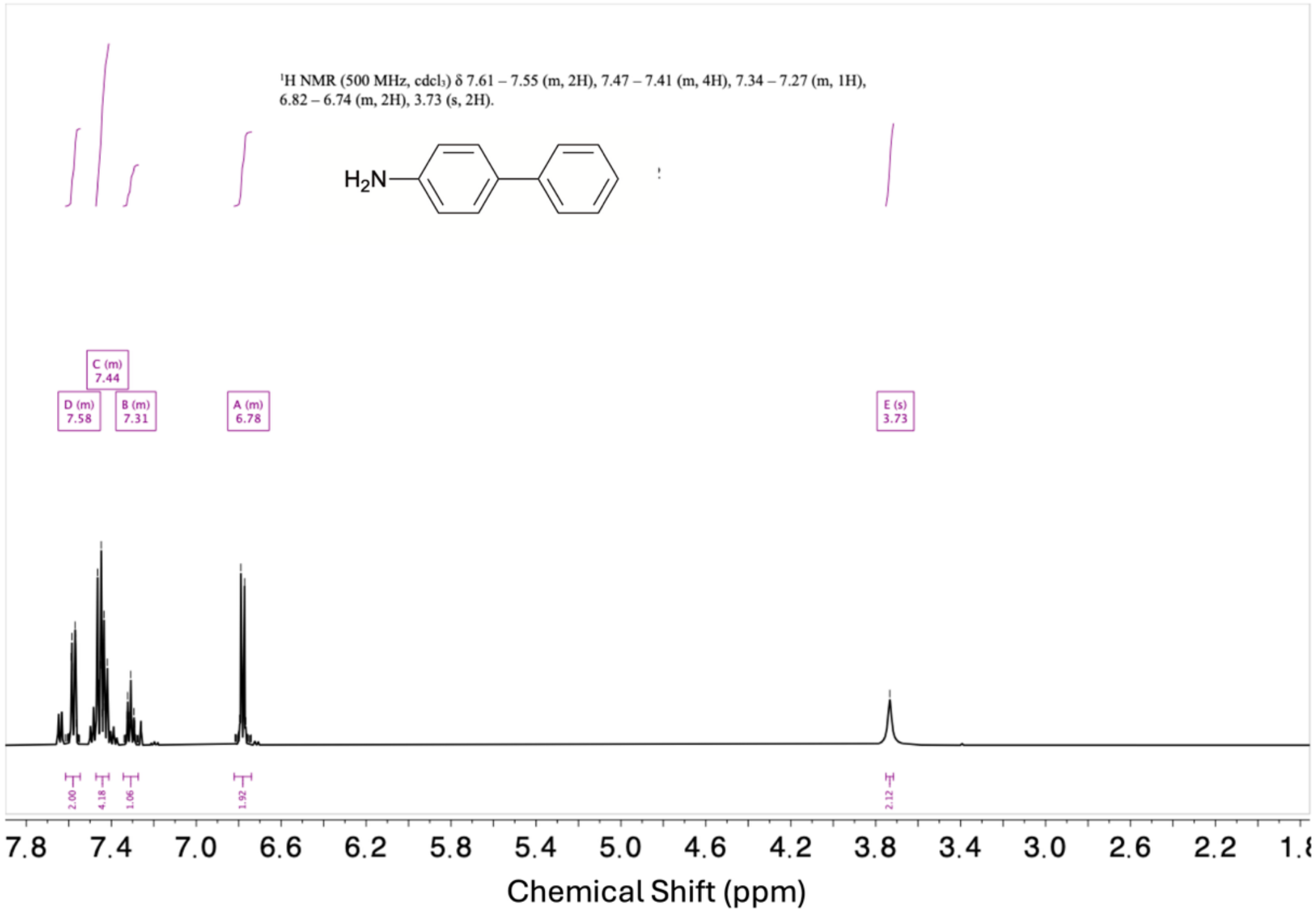

Fig. S36 ^{1}H NMR spectrum of 4-amino-1,1'-biphenyl in deuterated chloroform.

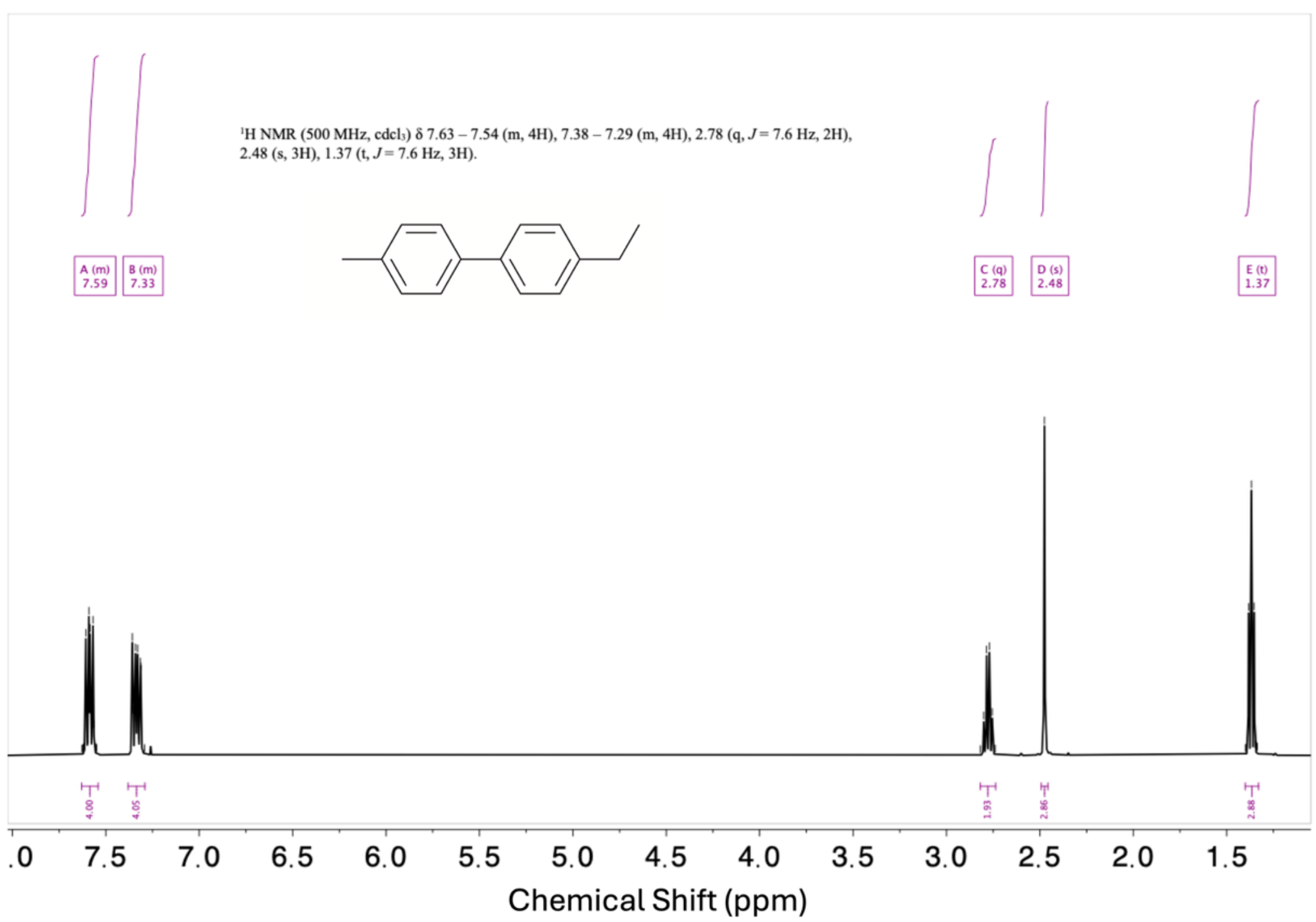

Fig. S37 ^{1}H NMR spectrum of 4-ethyl-4'-methyl-1,1'-biphenyl in deuterated chloroform.

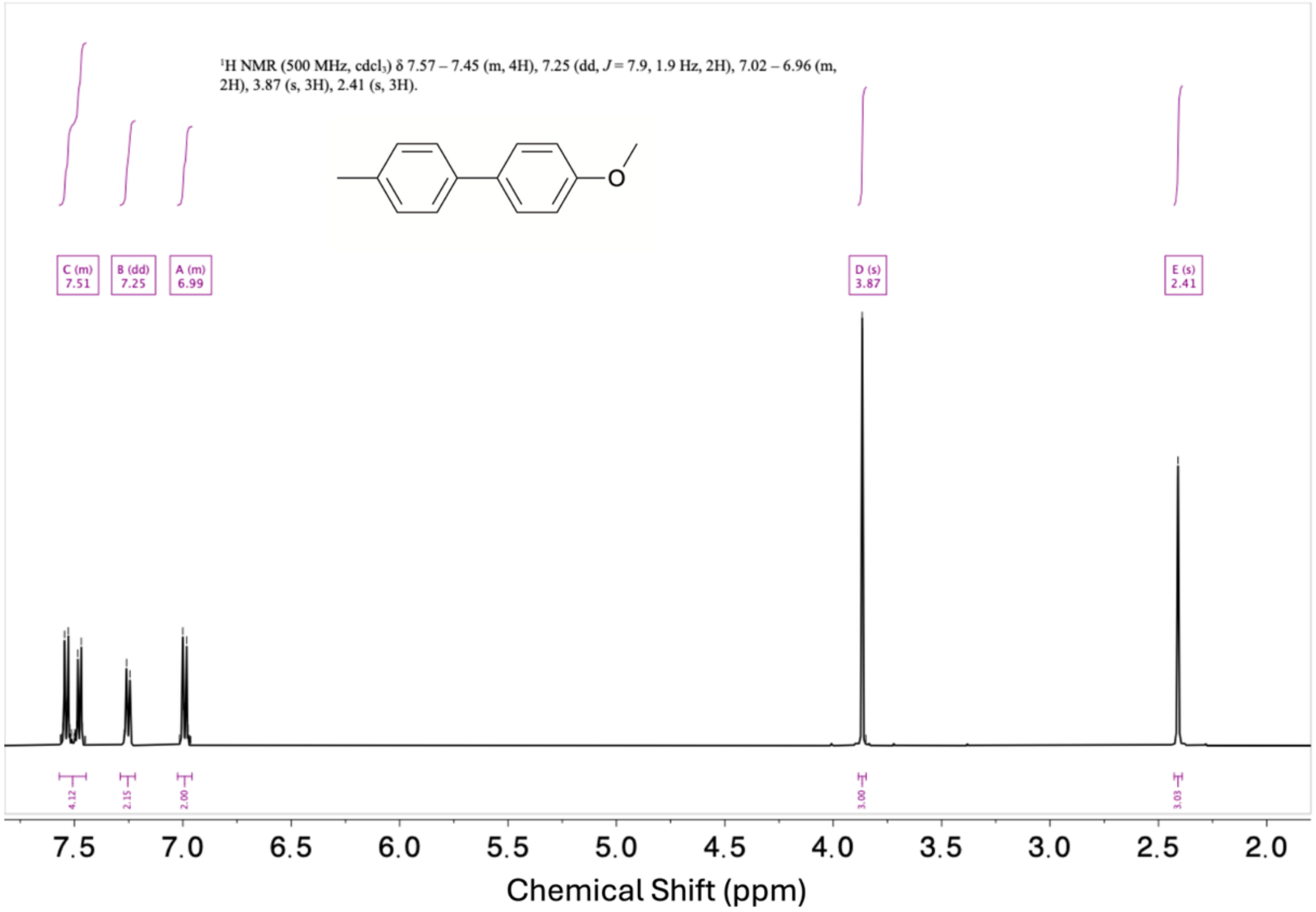

Fig. S38 ^{1}H NMR spectrum of 4-methoxy-4'-methyl-1,1'-biphenyl in deuterated chloroform.

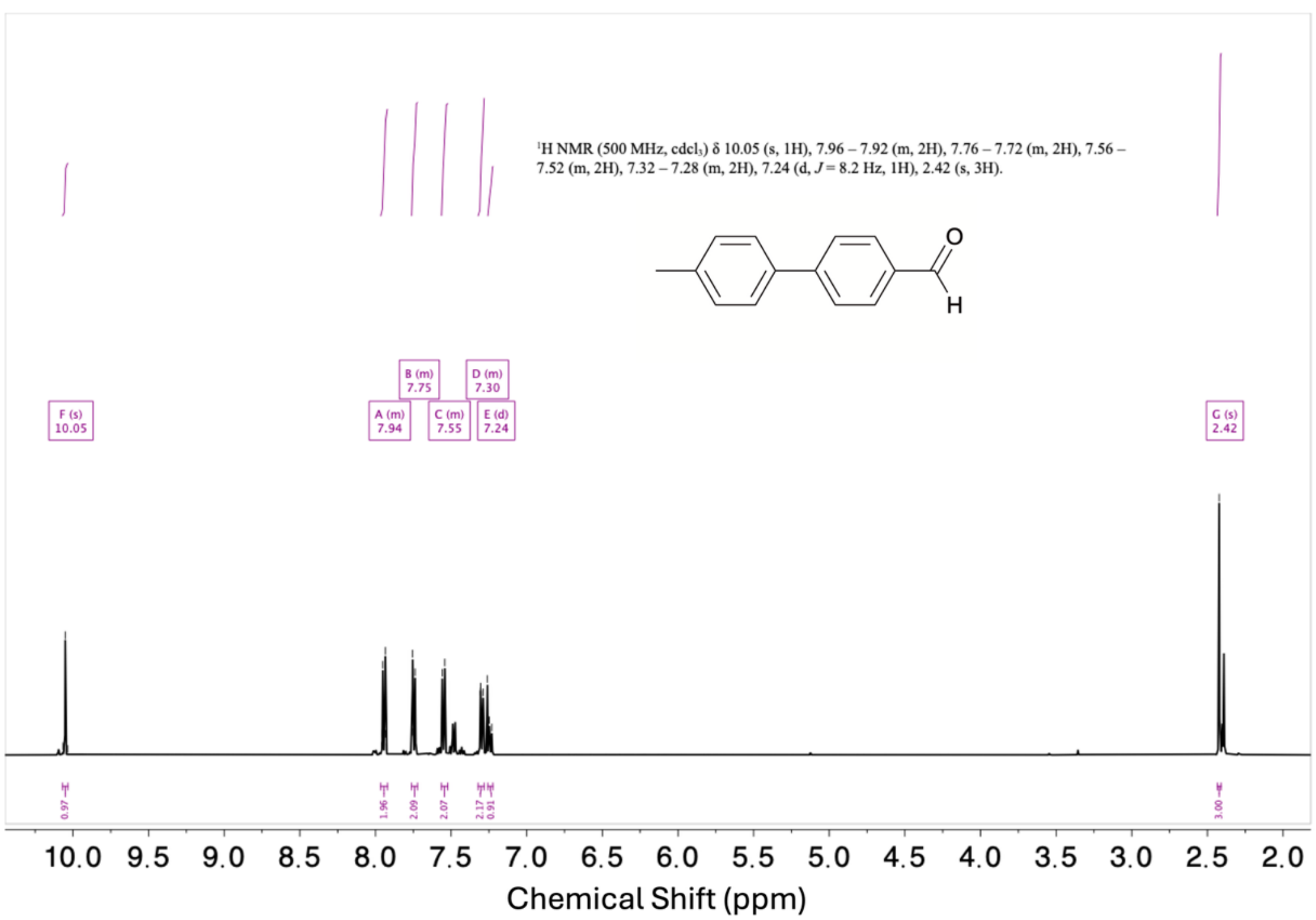

Fig. S39 ^{1}H NMR spectrum of 4-formyl-4'-methyl-1,1'-biphenyl in deuterated chloroform.

13. References

1. T. D. Kühne, M. Iannuzzi, M. Del Ben, V. V. Rybkin, P. Seewald, F. Stein, T. Laino, R. Z. Khaliullin, O. Schütt, F. Schiffmann *et al.*, *J. Chem. Phys.*, 2020, **152**, 194103.
2. G. Lippert, J. Hutter and M. Parrinello, *Mol. Phys.*, 1997, **92**, 477–488.
3. J. P. Perdew, K. Burke and M. Ernzerhof, *Phys. Rev. Lett.*, 1996, **77**, 3865–3868.
4. S. Grimme, S. Ehrlich and L. Goerigk, *J. Comput. Chem.*, 2011, **32**, 1456–1465.
5. S. Goedecker, M. Teter and J. Hutter, *Phys. Rev. B*, 1996, **54**, 1703–1710.
6. J. VandeVondele and J. Hutter, *J. Chem. Phys.*, 2007, **127**, 114105.
7. S. Nerdinger, C. Kendall, X. Cai, R. Marchart, P. Riebel, M. R. Johnson, C. F. Yin, N. Henaff, L. T. Eltis and V. Snieckus, *J. Org. Chem.*, 2007, **72**, 5960–5967.
8. F. C. Krebs, N. C. Schiødt, W. Batsberg and K. Bechgaard, *Synthesis*, 1997, **1997**, 1285–1290.
9. M. Albrecht and M. Schneider, *Synthesis*, 2000, **2000**, 1557–1560.
